# An Enhanced Educational Competition Optimizer Integrating Multiple Mechanisms for Global Optimization Problems

**DOI:** 10.3390/biomimetics10110719

**Published:** 2025-10-24

**Authors:** Na Li, Zi Miao, Sha Zhou, Haoxiang Zhou, Meng Wang, Zhenzhong Liu

**Affiliations:** 1 College of Literature, Yan’an University, Yan’an 716000, China; wxy@yau.edu.cn; 2 School of Digital Economics, Hebi Polytechnic, Hebi 458000, China; 99990425@hbzy.edu.cn; 3 School of Mathematics and Statistics, Xianyang Normal University, Xianyang 712000, China; zhousha@snnu.edu.cn; 4 Taizhou Institute of Zhejiang University, Taizhou 318000, China; zhouhaoxiang@tzizju.cn (H.Z.); wangmeng@tzizju.cn (M.W.)

**Keywords:** educational competition optimizer, regenerative population strategy, Powell mechanism, update framework, engineering constrained optimization

## Abstract

The Educational Competition Optimizer (ECO) formulates search as a three-stage didactic process—primary, secondary and tertiary learning—but the original framework suffers from scarce information exchange, sluggish late-stage convergence and an unstable exploration–exploitation ratio. We present EECO, which introduces three synergistic mechanisms: a regenerative population strategy that uses the covariance matrix of elite solutions to maintain diversity, a Powell mechanism that accelerates exploitation within promising regions, and a trend-driven update that adaptively balances exploration and exploitation. EECO was evaluated on the 29 benchmark functions of CEC-2017 and nine real-world constrained engineering problems. Results show that EECO delivers higher solution accuracy and markedly smaller standard deviations than eight recent algorithms, including EDECO, ISGTOA, APSM-jSO, LSHADE-SPACMA, EOSMA, GLSRIME, EPSCA, and ESLPSO. Across the entire experimental battery, EECO consistently occupied the first place in the Friedman hierarchy: it attained average ranks of 2.138 in 10-D, 1.438 in 30-D, 1.207 in 50-D, and 1.345 in 100-D CEC-2017 benchmarks, together with 1.722 on the nine real-world engineering problems, corroborating its superior and dimension-scalable performance. The Wilcoxon rank sum test confirms the statistical significance of these improvements. With its remarkable convergence accuracy and reliable stability, EECO emerges as a promising variant of the ECO algorithm.

## 1. Introduction

Optimization requirements are ubiquitous in science and engineering decision-making. Traditional approaches rely on deterministic mathematical programming: an explicit analytical model of objectives and constraints is first formulated, after which gradient-based or enumerative methods are employed [1]. However, when problem dimensionality increases and the objective landscape becomes multimodal, non-convex, or even discontinuous, these deterministic techniques are prone to the curse of dimensionality or entrapment in local optima [2]. Over the past two decades, metaheuristic algorithms (MAs) have emerged as a prominent alternative for tackling complex optimization models, owing to their problem-agnostic framework, lenient assumptions regarding the mathematical form of the objective, and inherent robustness to noise and uncertainty [3]. Representative applications include real-time dispatching in power systems [4,5], mission planning for unmanned aerial vehicles [6,7,8], deep and machine learning [9,10], threshold selection in image segmentation [11,12], coverage optimization in wireless sensor networks [13,14], engineering optimization problems [15,16], and resource scheduling in cloud computing [17,18,19]. In parallel or distributed computing environments, MAs demonstrate favorable scalability and consistently deliver high-quality approximate optima [20,21,22].

In the early development of metaheuristic algorithms, the research community usually categorized them into three major paradigms: evolution-based, swarm-based, and physics-based algorithms [23]. Evolutionary-based metaheuristics are exemplified by Genetic Algorithm (GA) [24], Differential Evolution (DE) [25], and Evolutionary Strategies (ES) [26]. Swarm-based algorithms constitute the largest subgroup within the metaheuristic family. Classical representatives include Particle Swarm Optimization (PSO) [27] and Ant Colony Optimization (ACO) [28]. Other algorithms include Rüppell’s Fox Optimizer (RFO) [29], Greylag Goose Optimization (GGO) [30], and Parrot Optimizer (PO) [31]. Physics-inspired algorithms trace their origins to the Simulated Annealing (SA) framework introduced by Metropolis et al. in 1953 [32]. Ongoing developments in the field have produced a growing suite of algorithms grounded in physical principles, including Mirage Search Optimization (MSO) [33], Fata Morgana Algorithm (FMA) [34], and Polar Lights Optimization (PLO) [35], thereby expanding the repertoire of this category.

**Figure 1:**
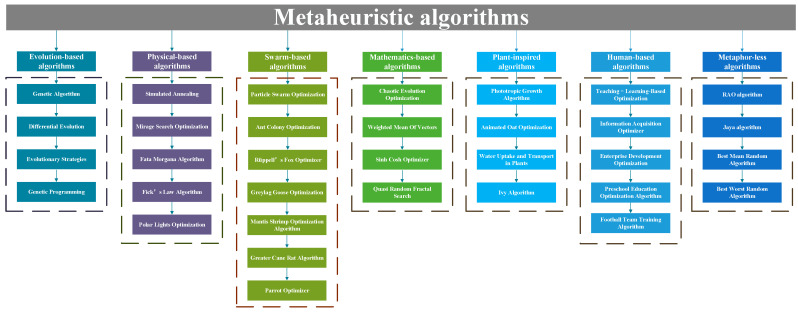
Summary of metaheuristic algorithms.

In recent years, new design inspirations continue to emerge, and mathematical mechanisms, plant behaviors, and human cognition have been successively abstracted into search strategies, resulting in mathematical-based algorithms, plant-based algorithms, and human-based algorithms. In addition, there are some algorithms that are difficult to categorize into any of the above categories, showing a more diversified development. Mathematics-based metaheuristics are grounded in rigorous principles such as analytical geometry, convex analysis, and game theory, giving rise to algorithms including Chaotic Evolution Optimization (CEO) [36] and Weighted Mean Of Vectors (WMOV) [37]. Plant-inspired algorithms constitute a recently emerging branch that leverages botanical metaphors—root foraging, phototropic branching, and seed dispersal—to design search operators; representative contributions include Phototropic Growth Algorithm (PGA) [38], Animated Oat Optimization (AOO) [39], and Water Uptake and Transport in Plants (WUTP) [40]. Human-based algorithms originated with the Teaching–Learning-Based Optimization (TLBO) [41]. Subsequent developments have introduced further human-centric variants, notably Information Acquisition Optimizer (IAO) [42], Enterprise Development Optimization (EDO) [43], and Football Team Training Algorithm (FTTA). In addition, there are metaphor-less metaheuristics, such as the RAO algorithm [44], the Jaya algorithm [45], the Best Mean Random (BMR) algorithm, and the Best–Worst Random (BWR) algorithm [46]. These seven categories of metaheuristic algorithms are summarized in Figure 1.

Drawing on the distinct learning phases experienced by students, Lian et al. (2024) introduced the Educational Competition Optimizer (ECO) [47]. The algorithm models three scholastic stages—primary, secondary, and tertiary—to guide population updates. Although ECO has demonstrated competitive performance on the CEC2021 benchmark suite, several limitations remain unaddressed. First, the absence of inter-population information exchange hampers the identification of promising regions during early search. Second, its convergence efficiency diminishes in later stages, preventing thorough exploitation of those regions. Third, the rigid sequential execution of the three scholastic phases induces an imbalance between exploration and exploitation. Collectively, these shortcomings restrict ECO’s capability to perform adequate global exploration and local refinement, rendering it susceptible to premature convergence on complex optimization landscapes [48]. To overcome ECO’s tendency to stagnate in local optima and its limited convergence accuracy, Emam et al. embedded a local escaping operator and a Gaussian-distribution perturbation strategy, which together enable the algorithm to jump out of premature convergence and refine the final solution quality [48]. To address these shortcomings, Tang et al. hybridized ECO with an estimation-of-distribution component and introduced a fitness–distance balance (FDB) criterion to filter individuals [49]. This combination significantly reinforces the global exploration capability of ECO while accelerating convergence efficiency. To enhance ECO’s ability to tackle complex, high-dimensional problems, Chen et al. introduced a jump strategy and an early-stopping mechanism that together accelerate convergence while maintaining exploration power [50]. This paper proposes an Enhanced Education Competition Optimizer (EECO) that integrates three complementary mechanisms: a regenerative population strategy guided by the covariance matrix of elite solutions to expand global coverage and enrich diversity; a Powell mechanism to intensify exploitation once promising areas are identified; and a trend-driven update framework that adaptively balances exploration and exploitation throughout the search process. In summary, the main contributions of this study are outlined as follows:

(1) This study introduces an enhanced version of ECO, referred to as EECO, which incorporates the regenerative population strategy, Powell mechanism, and trend-driven update framework.
(2) This study systematically verified the comprehensive advantages of EECO in convergence, robustness, and dimensional scalability through rigorous comparative experiments.
(3) Multiple statistical tests, such as the Wilcoxon rank sum test, the Friedman test, and the Nemenyi post hoc test, were used to analyze the data obtained from the EECO and comparison algorithms.

**Figure 2:**
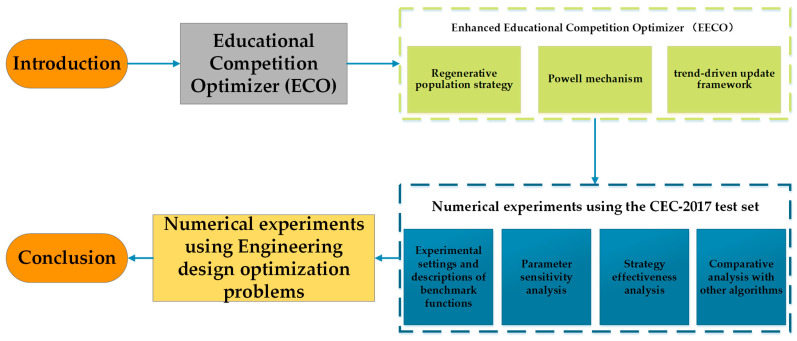
A complete outline of this work.

This paper is partially structured as follows: Section 2 provides a comprehensive background on the ECO algorithm. Section 3 elaborates on the three new mechanisms integrated into the ECO algorithm. In Section 4, a series of comparative experiments are conducted to evaluate the EECO algorithm. Section 5 describes the application of the EECO algorithm to engineering constrained optimization problems. Section 6 summarizes the chapter and suggests directions for future research. A comprehensive schematic of the paper’s organization is presented in Figure 2.

## 2. Educational Competition Optimizer

The ECO algorithm takes the competitive mechanism in the education system as a metaphor and divides the optimization process in the search process into three sequential academic stages—elementary, middle, and high school. Through adaptive search strategies, ECO effects a smooth transition from global exploration to focused exploitation. Its mathematical model is summarized as follows.

### 2.1. Population Initialization

The ECO algorithm initializes its population via the logistic chaotic map, distinguishing itself from other metaheuristics. This map emulates social disorder arising from educational deprivation. Given population size $N_{pop}$ and problem bounds lb (lower bound) and ub (upper bound), the initialization formula is expressed in Equation (1).

(1) $$X_{i}^{ini}=lb+\left(ub-lb\right)\times x_{i}$$

In Equation (1), $X_{i}^{ini}$ denotes the initial position of the $i^{th}$ agent, and $x_{i}$ represents the value of the logistic chaotic map, calculated by Equation (2).

(2) $$x_{i}=\alpha \times x_{i-1}\times \left(1-x_{i-1}\right),\;0\leq x_{0}\leq 1,i=1,2,\ldots ,N$$

where $x_{0}$ is a uniformly distributed random number in (0, 1), and α is a constant set to 4.

### 2.2. Primary School Stage

During the primary school stage, ECO partitions the population into two cohorts: the top 20% of individuals, ranked by fitness, form the school set, while the remaining 80% constitute the student set. Schools update their positions based on the mean location of their associated agents; students select the nearest school. This stage embodies initial exploration, with schools and students jointly surveying promising regions under constrained conditions, as formalized in Equations (3) and (4).

(3) $$X_{i}^{new}=X_{i}+\omega \times \left(X_{imean}-X_{i}\right)\times Levy\left(D\right)$$

(4) $$X_{i}^{new}=X_{i}+\omega \times \left(close\left(X_{i}\right)-X_{i}\right)\times randn$$

where $X_{i}$ denotes the current position of the $i^{th}$ agent. $X_{i}^{new}$ denotes its updated position. $X_{imean}$ denotes the average position of the $i^{th}$ agent, as shown in Equation (5). $Levy\left(D\right)$ is a D-dimensional vector obeying the Levy distribution, in which D is the dimension of the problem. It is defined by Equation (6). $close\left(X_{i}\right)$ indicates the location of the school closest to $X_{i}$. randn denotes a random number drawn from the standard normal distribution. ω is a variable that changes with the number of function evaluations and is obtained from Equation (7).

(5) $$X_{imean}=\frac{\sum_{j=1}^{D}X_{i,j}}{D}$$

(6) $$Levy\left(D\right)=\frac{\mu \times \sigma }{{\left|v\right|}^{2/3}},\;\mu ∼N\left(0,D\right),\;v∼N\left(0,D\right),\;\sigma ={\left(\frac{\Gamma \left(2.5\right)\times \sin\left(0.75\pi \right)}{1.5\times \Gamma \left(1.25\right)\times 2^{0.25}}\right)}^{2/3}$$

(7) $$\omega =0.1\times \ln\left(2-FEs/FEsMax\right)$$

where FEs and FEsMax denote the number of function evaluations and the maximum number of function evaluations, respectively.

### 2.3. Middle School Stage

In the middle school phase, ECO again splits the population into schools and students, now restricting schools to the top 10% of individuals by fitness while assigning the remaining 90% to the student cohort. Schools relocate by incorporating both the population mean and the best individual; students continue to select the nearest school and are additionally divided into two groups according to academic potential. These behaviors are formalized in Equations (8) and (9).

(8) $$X_{i}^{new}=X_{i}+\left(X_{best}-X_{mean}\right)\times e^{\left(\frac{FEs}{FEsMax}-1\right)}\times Levy\left(D\right)$$

(9) $$X_{i}^{new}=X_{i}-\omega \times close\left(X_{i}\right)-P\times \left(E\times \omega \times close\left(X_{i}\right)-X_{i}\right)$$

where $X_{best}$ is the best agent so far. $X_{mean}$ denotes the average position of all school agents and student agents. e denotes the natural exponential constant (Euler’s number). P denotes the patience of the student agents and is represented by Equation (10). E denotes the motivation to learn and is represented by Equation (11).

(10) $$P=4\times randn\times \left(1-FEs/FEsMax\right)$$

(11) $$E=\left\{\begin{array}{l} \frac{\pi }{P}\times \frac{FEs}{FEsMax},\;r_{1}>H\\ 1,\;r_{1}\leq H \end{array}\right.$$

where H is the judgment threshold with a value of 0.5. $r_{1}$ is a random variable uniformly distributed in [0, 1].

### 2.4. High School Stage

In the high school phase, the proportions of the two subpopulations remain identical to those in the middle school phase. School agents update their positions cautiously by integrating the average, best, and worst individuals, thereby addressing the broader needs of the student cohort. Conversely, student agents enroll in the currently top-performing school. The corresponding behaviors are formalized in Equations (12) and (13).

(12) $$X_{i}^{new}=X_{i}+\left(X_{best}-X_{mean}\right)\times randn-\left(X_{worst}-X_{mean}\right)\times randn$$

(13) $$X_{i}^{new}=X_{i}-P\times \left(E\times X_{best}-X_{i}\right)$$

where $X_{worst}$ denotes the worst-performing agent observed thus far.

### 2.5. Selection and Greed Mechanisms

In the ECO algorithm, the three strategies are selected in rotation following the increase in iterations. Specifically, when mod(t,3) is equal to 1, all agents will select the primary-stage updating method in this round of iterations. When mod(t,3) is equal to 2, all agents will select the middle-school-stage updating method in this round of iteration. When mod(t,3) is equal to 0, all agents will select the high-school-stage updating method in this round of iteration. In addition, the ECO algorithm uses a greedy mechanism to filter the agents, which means that the agents before and after updating will be compared with each other and the better one will be kept for the next iteration.

## 3. Proposed Enhanced Educational Competition Optimizer

Although the ECO algorithm shows promise as a metaheuristic algorithm, it still shows limitations in complex optimization scenarios: imbalance between exploration and exploitation, susceptibility to local extremes, and insufficient convergence accuracy. In this paper, we propose the EECO algorithm, which introduces three improvements, namely regenerative population strategy, Powell mechanism, and trend-driven update framework, to balance exploration and exploitation, facilitate information exchange, and reorganize the update framework to improve the overall performance. In this section, the structure of the EECO algorithm is described in detail, followed by pseudo-code and flowcharts, and finally its computational complexity is analyzed.

### 3.1. Regenerative Population Strategy

Metaheuristic algorithms need to balance exploration and exploitation to achieve a comprehensive scan and accurate mining of the solution space. However, the search operators of most algorithms implicitly prefer specific regions: without being guided by the objective function, the strategies or operators may prompt the population to cluster prematurely in localized regions, resulting in overexploitation and weakening the search ability for alternative solutions. The ECO algorithm, as a human-based metaheuristic algorithm, is inevitably constrained by these same problems. In order to improve its global exploration performance and enhance the population diversity and adaptability, this paper proposes a regenerative population strategy (RPS) to make the ECO algorithm more robust in complex landscapes with low total variation, ruggedness, and needle-in-a-haystack problems. After each iteration, the RPS selects a subset of individuals for regeneration, thereby fostering continued exploration of the solution space. The proportion to be regenerated is determined jointly by current population diversity and the observed improvement rate.

Population diversity (PD), denoted as τ which quantifies the dispersion of individuals across the search space and is therefore critical for global exploration, is computed by Equation (14).

(14) $$\tau =\sum_{i=1}^{N}\left\|X_{i}-X_{mean}\right\|$$

The improvement rate (IR), denoted as ς quantifies the search progress of the population and is given by Equation (15).

(15) $$\varsigma =\frac{F\left(X_{best}^{t}\right)-F\left(X_{best}^{t+1}\right)}{F\left(X_{best}^{t+1}\right)}$$

Upon obtaining PD and IR, the composite score S of the current population is expressed as the normalized weighted sum of these two indices, as shown in Equation (16).

(16) $$S=\lambda_{1}\times \frac{\tau }{\tau_{\max}}+\lambda_{2}\times \frac{\varsigma }{\varsigma_{\max}}$$

In the equation, $\tau_{\max}$ denotes the maximal PD observed so far, and $\varsigma_{\max}$ the maximal IR encountered to date. $\lambda_{1}$ and $\lambda_{2}$ are the weights assigned to the two indices. In this, both weights are set to 0.5, signifying equal importance. In the RPS, the number of individuals to be regenerated at each iteration is determined by Equation (17). Once this quantity is fixed, exactly $N_{RPS}$ distinct individuals—excluding the current best—are selected uniformly at random and re-initialized within the search space according to Equation (18).

(17) $$N_{RPS}=\left\lfloor \left(1-S\right)\times \left(N-1\right)\right\rfloor$$

(18) $$X_{i}=\mu +g_{i},g_{i}\sim N\left(0,C\right)$$

Here, the symbol $\left\lfloor \cdot \right\rfloor$ denotes the greatest integer function. μ is the weighted centroid of the top 50% fittest individuals, and C is the corresponding covariance matrix computed from this elite subset, capturing the population’s evolutionary trend. Each agent is assigned a weight θ proportional to its fitness, so that better-performing agents exert stronger influence on the inferred direction of evolution.

(19) $$C=\frac{2}{N}\times \sum_{i=1}^{0.5N}\left(X_{i}-\mu \right)\times {\left(X_{i}-\mu \right)}^{T}$$

(20) $$\mu =\frac{2}{N}\times \sum_{i=1}^{0.5N}\left(\theta_{i}\times X_{i}\right)$$

(21) $$\theta_{i}=\ln\left(0.5N+1\right)/\left(\sum_{i=1}^{0.5N}\left(\ln\frac{\left(0.5N+1\right)}{i}\right)\right)$$

The RPS dynamically adjusts the number of individuals to be regenerated according to the current population quality, thereby promoting a balanced trade-off between exploitation and exploration. Moreover, the incorporation of the covariance matrix enhances population quality and strengthens global exploration.

### 3.2. Powell Mechanism

Excessive global exploration can degrade convergence; to restore the exploitation–exploration balance of the ECO algorithm, this work employs the Powell mechanism (PM) to intensify exploitation. Powell’s method is a potent local-search technique that exploits conjugate directions to accelerate convergence. Introduced into the late phase of ECO, it strengthens the population’s local search capacity and increases the likelihood of locating the global optimum. The procedure comprises three sequential stages: basic search, acceleration search, and adjustment search. In each iteration, basic search begins from the current position and performs one-dimensional searches along the existing directions to generate a new position vector. Acceleration search computes the difference between two consecutive position vectors to obtain a direction closer to the optimum, replacing the original search direction. Finally, adjustment search substitutes one of the current directions with the connecting direction obtained during acceleration, thereby forming a renewed direction set for the next iteration. This cycle repeats until a precise solution is attained. The specific implementation of the Powell mechanism is as follows.

Step 1: Initialization. Select an initial point $\gamma^{\left(0\right)}$ and linearly independent search directions D. Prescribe a convergence tolerance Err>0 and set k=0.

Step 2: Basic search. Compute $\delta_{i}$ using Equation (22), then successively generate new base points $\gamma^{\left(1\right)}$, $\gamma^{\left(2\right)}$, …, $\gamma^{\left(D\right)}$ along the respective dimensions, as specified in Equation (23).

(22) $$F\left(\gamma^{\left(i\right)}+\delta_{i}\times d^{\left(i\right)}\right)=\min\left(F\left(\gamma^{\left(i\right)}+\delta_{i}\times d^{\left(i\right)}\right)\right)$$

(23) $$\gamma^{\left(i+1\right)}=\gamma^{\left(i\right)}+\delta_{i}\times d^{\left(i\right)},\;i=0,1,\ldots ,D-1$$

In the equation, $\delta_{i}$ denotes the search step sizes. If $\delta_{i}$ is negative, a line search is performed along the corresponding axis. If i<D−1, i is incremented by 1 and Step 2 is repeated; otherwise, Step 3 is executed.

Step 3: Acceleration search. Compute the acceleration direction $d^{\left(D\right)}=\gamma^{\left(D\right)}-\gamma^{\left(0\right)}$. If the termination criterion on $\left\|d^{\left(D\right)}\right\|<Err$ is satisfied, exit; otherwise, proceed to Step 4.

Step 4: Compute the maximum-descent index tl using Equation (24). If Equation (25) is satisfied, the search directions for the next cycle remain unchanged, in which case set $\gamma^{\left(0\right)}=\gamma^{\left(D\right)}$, k=k+1, and proceed to Step 2; otherwise, Step 5 is executed.

(24) $$F\left(\gamma^{\left(tl\right)}\right)-F\left(\gamma^{\left(tl+1\right)}\right)=\underset{0\leq i\leq D-1}{\max}\left\{F\left(\gamma^{\left(i\right)}\right)-F\left(\gamma^{\left(i+1\right)}\right)\right\}$$

(25) $$F\left(\gamma^{\left(0\right)}\right)-2\times F\left(\gamma^{\left(D\right)}\right)+F\left(2\times \gamma^{\left(D\right)}-\gamma^{\left(0\right)}\right)\geq 2\times \left(F\left(\gamma^{\left(tl\right)}\right)-F\left(\gamma^{\left(tl+1\right)}\right)\right)$$

Step 5: Adjusted search. Set $\gamma^{\left(tl+i\right)}=\gamma^{\left(tl+i+1\right)}$ to ensure the newly generated exploration directions remain linearly independent, then compute $\delta_{D}$ via Equation (22). Set $\gamma^{\left(0\right)}=\gamma^{\left(D+1\right)}=\gamma^{\left(D\right)}+\delta_{D}\times d^{\left(D\right)}$, k=k+1, and proceed to Step 2. The PM method is used to further excavate promising regions, so PM is performed when FEs>a×FEsMax. The parameter a will be determined in subsequent experiments.

### 3.3. Trend-Driven Updating Framework

ECO’s original search framework sequentially executes the elementary, middle, and high school strategies in strict cyclic order, without regard to the current population’s actual needs. This blind rotation hampers overall performance. To remedy this, we introduce a trend-driven updating framework, denoted as TUF. The TUF logs whether each agent’s previously chosen phase succeeded; success triggers reuse, while failure prompts a random switch to one of the remaining two phases. Compared with the fixed rotation, A accelerates convergence and raises optimization efficiency by favoring historically successful strategies, thereby guiding the population toward more promising regions and making better use of limited computational resources.

### 3.4. The Structure of EECO and Its Time Complexity

**Figure 3:**
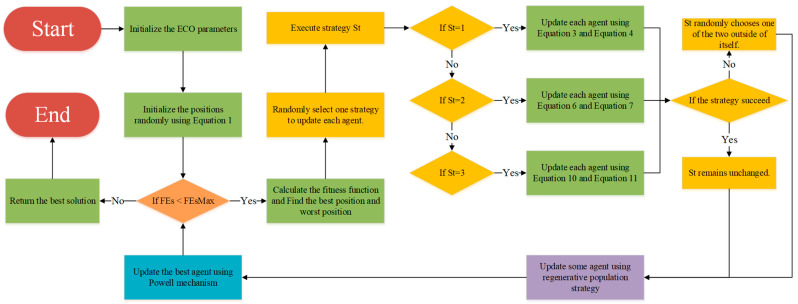
Flowchart of the EECO algorithm.

This subsection presents the detailed workflow of the proposed EECO algorithm. The algorithm first generates the initial population using a logistic chaotic map. A trend-driven updating framework then dynamically selects the appropriate stage, replacing the former iteration-based schedule. Regenerative population strategy regenerates a subset of agents to enhance global exploration, while the Powell mechanism is activated in the later phase to intensify local exploitation of promising regions. The detailed flowchart of the EECO algorithm can be found in Figure 3, and the pseudo-code is illustrated in Algorithm 1.
**Algorithm 1**: Pseudo-code of EECO algorithm1: Initialize the ECO parameters2: Initialize the population *X* using Equation (1)3: **While** *FEs* < *FEsMax*4: Calculate the fitness function5: Find the best position and worst position6: Randomly select one strategy to update each agent7: Execute strategy according to the value of St8: **For**
*i* = 1: *N* **do**9:      **If** St = 1 **Then //** Stage 1: Primary school10:      Update each agent using Equations (3) and (4)11:    **End if**12:    **If** St = 2 **Then //** Stage 2: Middle school13:      Update each agent using Equations (8) and (9)14:    **End if**15:    **If** St = 3 **Then //** Stage 3: High school16:      Update each agent using Equations (12) and (13)17:    **End if**18: **End for**
19: Update the St using trend-driven updating framework // **TDUF**20: *FEs* = *FEs* + *N*21: Update some agent using regenerative population strategy // **RPS**22: **If** FEs > 0.9 × FEsMax **Then**
23: Update the best agent using Powell mechanism // **PM**24: *FEs* = *FEs* + *mxit*^2^25: **End if**26: **End while**
27: Return the best solution

This paper integrates three improvement mechanisms based on the basic ECO algorithm, so it is necessary to analyze the time complexity of the proposed EECO algorithm. According to the literature [47], the time complexity of the ECO algorithm consists of three parts: population initialization, fitness computation, and position update strategy. Assuming that the number of populations is N, the problem dimension is D, and the number of iterations is T, then the time complexity of the ECO algorithm is $O\left(N\times D+T\times N\times D+T\times N\times \log N\right)$. For the EECO algorithm, there is no change in the initialization phase and the three update strategies, so there is no increase in time complexity for this part. Assuming that the RPS replaces $N_{1}$ agents, the time complexity of this part is $O\left(T\times N_{1}\times D+T\times N_{1}\times \log N_{1}\right)$. The time complexity of the PM is $O\left(T\times mxit^{2}\right)$, where A denotes that it is executed mxit times. The TDUF changes the selection framework of the original strategies, and it does not involve additional location updates and adaptation calculations, so there is no increase in time complexity. In conclusion, the time complexity of the EECO algorithm is $O\left(N\times D+T\times \left(N+N_{1}\right)\times D+T\times \left(N\times \log N+N_{1}\times \log N_{1}\right)+T\times mxit^{2}\right)$.

## 4. Numerical Experiments Using the CEC-2017 Test Set

In this section, we comprehensively evaluate the performance of the EECO algorithm on the CEC-2017 test set. Section 4.1 describes the experimental setup and details of the CEC-2017 test set. A parameter sensitivity analysis is performed in Section 4.2 to determine the optimal parameter settings for the EECO algorithm. Section 4.3 provides the results of the ablation experiments. In Section 4.4, we examine the structural bias of both the EECO and ECO algorithms. In Section 4.5, a comprehensive comparison between the EECO algorithm and several advanced algorithms is presented.

### 4.1. Experimental Settings and Descriptions of Benchmark Functions

All experiments were conducted on a 2.50 GHz AMD R9-7945HX CPU with 32 GB RAM and an RTX 4060 GPU, under Windows 11 and MATLAB 2023a. For every benchmark test function, the maximum number of function evaluations was set to 1000D, and each algorithm was run independently 30 times to mitigate stochastic variability.

Benchmark functions are indispensable for evaluating algorithmic performance and provide a standardized platform for comparing diverse optimization methods. To comprehensively assess the capabilities of the proposed EECO algorithm, we adopt the CEC-2017 test suite at dimensions 10, 30, 50, and 100. Increasing dimensionality entails a rapid proliferation of local optima, thereby imposing a more stringent test on global search ability. The CEC-2017 suite comprises 29 functions categorized into four classes: unimodal (F1–F2), multimodal (F3–F9), hybrid (F10–F19), and composition (F20–F29). Unimodal functions possess a single global optimum and no local optima, making them suitable for gauging exploitation intensity. Multimodal functions contain numerous local optima and are primarily used to evaluate an algorithm’s capacity to locate the global optimum and to escape local basins. Hybrid and composition functions emulate highly intricate continuous landscapes, thereby assessing the combined ability of an algorithm to perform both local refinement and global exploration. Detailed specifications of all functions are provided in Table 1. The complete mathematical formulation is available in Reference [51].

During the experimental section, the best value (Best), the average value (Ave), the standard deviation (Std), and the ranking (Rank) obtained by all the algorithms on each of the CEC-2017 functions were fully documented. Given that detailed tables would significantly increase the length of the main text, the raw data have been transferred to the Appendix A. In the main section, we dissect these results primarily using the Wilcoxon rank sum test, the Friedman test, and the Nemenyi post hoc test. The Wilcoxon rank sum test is used to pairwise compare the results of two algorithms over the full range of test functions to determine which performs better in a statistically significant way, while the Friedman test is used for the entire experimental group to comprehensively assess whether the differences between all algorithms at the overall level are significant. If the Friedman test shows significant differences, the Nemenyi post hoc test further utilizes a critical difference plot to pinpoint specific gaps between the algorithms. It should be emphasized that all statistical tests set the significance level α to 0.05 to ensure the reliability of the conclusions.

### 4.2. Parameter Sensitivity Analysis

#### 4.2.1. The Analysis of Parameter N

**Figure 4:**
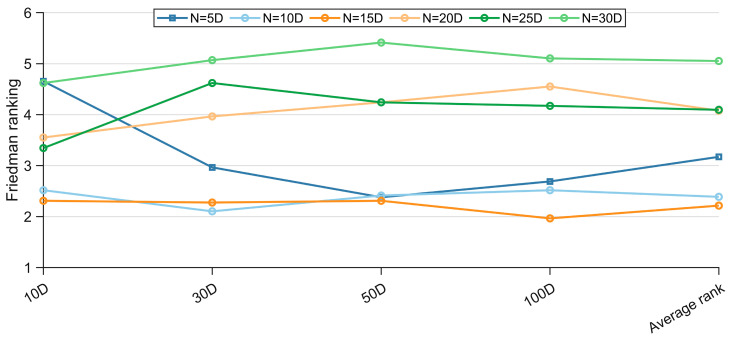
Friedman rankings of the EECO with different population sizes.

Appropriate population sizing is a decisive factor in unlocking the full potential of metaheuristic algorithms. Operating under a fixed budget for function evaluations, these optimizers must deliver high-quality solutions within limited resources; consequently, identifying a parsimonious yet sufficient population size becomes critical. If the size is too large, the computational resources will be unnecessarily consumed in the early stage; if the size is too small, it is difficult to cover the entire solution space, and both will compromise the performance of the algorithm. Moreover, the proposed RPS relies on the covariance matrix of the elite sub-population to infer evolutionary directions. If the number of individuals is insufficient, the sample noise will overwhelm the true distribution, leading to overfitting of the covariance matrix, and the algorithm is prone to falling into local extremes or generating oscillations, with a consequent weakening of the global exploration capability. Hence, RPS imposes its sensitivity window on population magnitude. Under a uniform cap on function evaluations, this section systematically examines the performance of the EECO algorithm across population sizes of 5D, 10D, 15D, 20D, 25D, and 30D, accounting for problem dimensions ranging from 10D to 100D. Comprehensive results on the CEC-2017 test suite are tabulated in Table A1, Table A2, Table A3 and Table A4 of Appendix A, while Figure 4 depicts the corresponding Friedman average rankings, revealing the impact of population scaling on algorithmic efficacy.

As Figure 4 illustrates, the performance of the EECO algorithm first deteriorates and then improves as the population size increases, confirming that both undersized and oversized populations are detrimental. Overall, the EECO algorithm attains its best results with a population of 15D, yielding an average Friedman rank of 2.215. Moreover, the ranking curves across different dimensions exhibit a consistent pattern, validating the dimension-scaled population sizing adopted herein. Consequently, all subsequent experiments employ a population size of 15D to fully exploit the capabilities of the EECO algorithm.

#### 4.2.2. The Analysis of Parameter a

**Figure 5:**
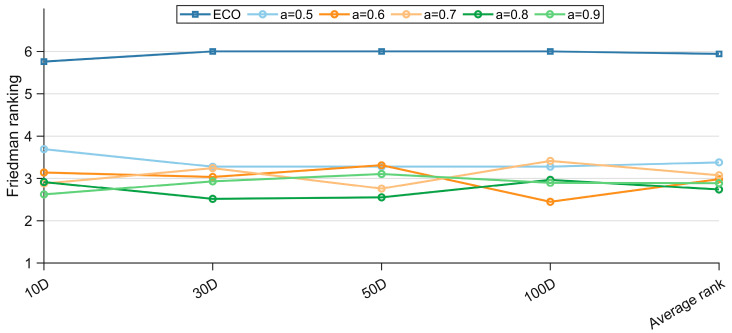
Friedman rankings of the EECO with different parameters a.

This work introduces the PM method only in the later search phase to perform high-precision, fast-converging local refinement around the incumbent solutions. Nevertheless, PM’s reliance on conjugate-direction searches renders it less effective on high-dimensional, ill-conditioned, or non-convex problems: its cost grows rapidly with dimensionality, and as a purely local method, it lacks any mechanism for escaping local optima once trapped. Consequently, PM is not employed throughout the entire optimization run. Algorithm 1 activates it only when a predefined progress indicator signals that exploitation is warranted. To determine the most advantageous activation point, we examine the EECO under various threshold values on the CEC-2017 benchmark suite; the resulting statistics are consolidated in Table A5, Table A6, Table A7 and Table A8 of Appendix A, and the corresponding Friedman average ranks are visualized in Figure 5 to illustrate how the timing of PM engagement influences overall algorithmic performance.

Figure 5 reveals that the later the PM is activated, the better the EECO performs. This observation aligns with our analysis: premature invocation of the Powell mechanism elevates computational overhead and hampers overall efficiency, whereas deferring its use safeguards exploratory breadth. Consequently, the activation threshold a is fixed at 0.8, enabling PM only during the final 20% of the search budget to conduct intensified exploitation of promising basins.

### 4.3. Strategy Effectiveness Analysis

The EECO algorithm augments the original ECO by integrating three complementary mechanisms: a regenerative population strategy (RPS), the Powell mechanism (PM), and a trend-driven update framework (TDUF). To quantify the individual contribution of each component, we conduct a systematic ablation study using six EECO variants whose configurations are summarized in Table 2. In this table, the first row lists the variant labels, and the first column enumerates the adopted strategies; “Yes” indicates inclusion, whereas “No” denotes exclusion of the corresponding mechanism. Such experiments play a pivotal role in verifying the robustness and dependability of research findings. By selectively excluding specific components or factors and observing the resulting changes in performance, researchers can better isolate the effects of each mechanism, thus eliminating alternative explanations. This process helps to clarify the individual contribution of each component, ensuring that the conclusions drawn are both valid and reproducible.

**Figure 6:**
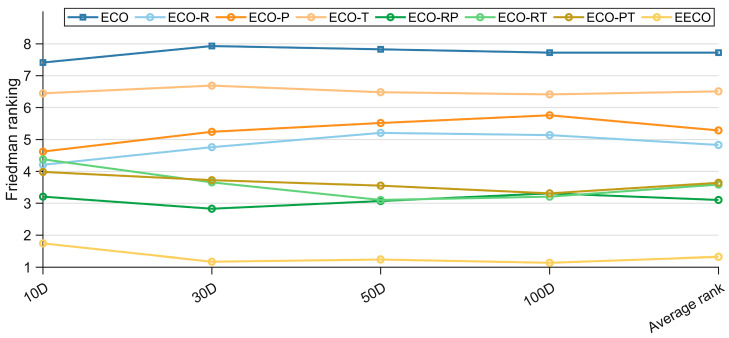
Friedman rankings of EECO with different strategies.

The ablation-study results for the EECO algorithm are consolidated in Table A9, Table A10, Table A11 and Table A12 of Appendix A. Table 3 summarizes the Friedman test results for EECO against its ablated variants, and the corresponding ranking is visually presented in Figure 6. A statistically significant difference exists between EECO and the compared variants, as indicated by the *p*-values in the final column. In light of these Friedman outcomes, the following conclusions can be drawn. (1) Across four-dimensional settings, the fully integrated EECO—employing all three enhancement strategies—achieves the best overall performance, attaining mean Friedman ranks of 1.741, 1.172, 1.241, and 1.138, respectively. (2) When ECO is compared with its single-strategy variants, all three improvements prove individually effective. (3) Among the variants that omit exactly one component, the rank ordering indicates that the RPS contributes most to EECO’s performance, followed by the PM, while the TDUF yields the smallest incremental gain. (4) A dimension-wise comparison reveals that TDUF is largely insensitive to increasing dimensionality, demonstrating strong scalability; conversely, RPS and PM confer greater benefits in lower-dimensional problem instances.

**Figure 7:**
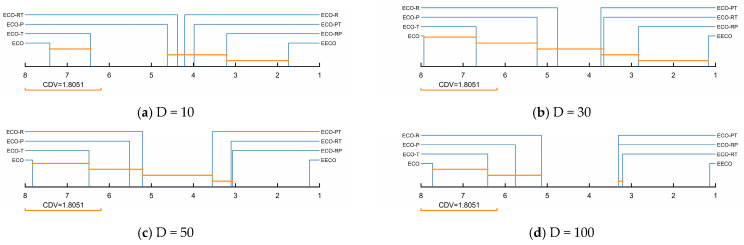
Nemenyi post hoc test of EECO with different strategies.

To quantify the individual contributions of the three strategies to ECO, a Nemenyi post hoc test was conducted, with the results shown in Figure 7. This test extends the Friedman finding of an overall difference by computing the critical difference value (CDV) via Equation (26), where M denotes the number of algorithms and K the number of benchmark functions. According to Nemenyi’s criterion, any pair of algorithms whose average ranks differ by less than CDV are not significantly different. Figure 7 reveals that EECO is not significantly different from ECO-RT at 10D and 30D, indicating that TDUF provides limited improvement in low-dimensional cases. Similarly, ECO-T does not differ significantly from ECO across all four-dimensional settings, implying that TDUF’s enhancement, while beneficial, is modest. Finally, the three two-strategy variants of ECO exhibit no significant pairwise differences, confirming that the strategies act synergistically rather than restrictively.

(26) $$CDV=q_{a}\times \sqrt{\frac{K\left(K+1\right)}{6M}}$$

Moreover, this section assesses the scalability of EECO by contrasting its performance with that of the basic ECO across multiple dimensional settings of the CEC-2017 benchmark. Scalability analysis is instrumental in understanding how an evolutionary algorithm adapts to increasing problem size and complexity. By systematically varying dimensionality, we evaluate the algorithm’s ability to maintain both efficiency and solution quality under escalating computational demands. The tests examine computational cost, execution time, and solution accuracy, thereby offering detailed insights into the practical feasibility and inherent limitations when tackling large-scale optimization tasks. Such evaluation is crucial for validating real-world applicability, as scalable algorithms are better suited for the high-dimensional, complex problems ubiquitous in industrial and engineering domains. Summarized in Table 3, the results reveal that EECO consistently outperforms ECO across all tested dimensions, demonstrating its robustness and adaptability in large-scale optimization scenarios. Table 4 presents the Wilcoxon rank sum test results between EECO, its ablated variants, and the baseline ECO, where the symbols “+/=/−” indicate the number of functions on which EECO or its variants outperform, match, or underperform ECO. The data reveal that neither RPS nor TDUF degrades ECO’s performance on any function, whereas PM alone occasionally worsens the outcome. Once PM is coupled with either RPS or TDUF, no further inferior results are observed, confirming that RPS and TDUF possess sufficient transferability to compensate for PM’s deficiencies. Consequently, all three proposed enhancement mechanisms are validated as effective.

### 4.4. Structural Bias Analysis of EECO

Structural bias is a common flaw in metaheuristic algorithms, referring to the tendency of an algorithm to favor solutions in specific regions of the search space [52]. In this subsection we investigate the structural bias exhibited by the EECO and ECO algorithms using the center–offset method (CO). Specifically, we conduct the experiment on the Griewank function and shift its global optimum according to Equation (27). The function dimensions are 30 and 100.

(27) $$f\left(x,c\right)=1+\frac{1}{4000}\sum_{j=1}^{D}{\left(x_{j}-c_{j}\right)}^{2}-\prod_{j=1}^{D}\cos\left(\frac{x_{j}-c_{j}}{\sqrt{j}}\right)$$

where $c=\left(c_{1},c_{2},\ldots ,c_{D}\right)$ represents the coordinate of the new center. The results of 30 independent runs on this function for both the EECO and ECO algorithms are reported in Table 5. According to Table 5, the ECO algorithm attains its highest accuracy when $c=\left(100,100,\ldots ,100\right)$, indicating a pronounced preference for boundary regions. In contrast, the EECO algorithm achieves the best results when $c=\left(0,0,\ldots ,0\right)$ and $c=\left(100,100,\ldots ,100\right)$, and performs comparably well in the remaining three cases. Overall, EECO exhibits no evident structural bias.

### 4.5. Comparative Analysis with Other Algorithms

This subsection benchmarks EECO against seven advanced algorithms—EDECO, ISGTOA [53], APSM-jSO [54], LSHADE-SPACMA [55], EOSMA [56], GLSRIME [57], EPSCA [58], and ESLPSO [59]—on the CEC-2017 test suite. To ensure impartiality, all competing algorithms are configured exactly as reported in their original publications, with parameter values listed in Table 6; any remaining experimental settings follow the specifications given in Section 4.1. Comprehensive evaluation is undertaken via the Wilcoxon rank sum test, Friedman test, Nemenyi post hoc test, convergence analysis, and robustness analysis. All raw numerical results are compiled in Table A13, Table A14, Table A15 and Table A16 of Appendix A.

Table 7 summarizes the Wilcoxon rank sum test results between EECO and the competing state-of-the-art algorithms, where “+” denotes that EECO significantly outperforms the rival, “−” indicates the opposite, and “=” signifies no statistically significant difference. Across all pairwise comparisons, EECO accumulates far more “+” than “−” or “=”, evidencing its consistent superiority across the benchmark functions and thereby corroborating its robustness and efficiency in tackling complex optimization tasks. The detailed analysis of the Wilcoxon rank sum test is presented below.

For 10D, EECO is superior (inferior) to EDECO, ISGTOA, APSM-jSO, LSHADE-SPACMA, EOSMA, GLSRIME, EPSCA, and ESLPSO on 25(0), 25(0), 23(2), 23(2), 23(2), 23(1), 21(0), and 24(1) test functions. That is, the EECO algorithm is dominant in at least 21 functions when compared to different algorithms when solving CEC-2017 test functions with 10D.

For 30D, EECO is superior (inferior) to EDECO, ISGTOA, APSM-jSO, LSHADE-SPACMA, EOSMA, GLSRIME, EPSCA, and ESLPSO on 29(0), 29(0), 26(3), 26(2), 29(0), 28(0), 25(2), and 26(2) test functions. That is, the EECO algorithm is dominant in at least 25 functions when compared to different algorithms when solving CEC-2017 test functions with 30D.

For 50D, EECO is superior (inferior) to EDECO, ISGTOA, APSM-jSO, LSHADE-SPACMA, EOSMA, GLSRIME, EPSCA, and ESLPSO on 29(0), 29(0), 27(2), 27(2), 29(0), 29(0), 23(0), and 27(1) test functions. That is, the EECO algorithm is dominant in at least 23 functions when compared to different algorithms when solving the CEC-2017 test functions with 50D.

For 100D, EECO is superior (inferior) to EDECO, ISGTOA, APSM-jSO, LSHADE-SPACMA, EOSMA, GLSRIME, EPSCA, and ESLPSO on 29(0), 28(0), 24(2), 24(2), 29(0), 27(0), 21(0), and 24(2) test functions. That is, the EECO algorithm is dominant in at least 21 functions when compared to different algorithms when solving CEC-2017 test functions with 100D.

**Figure 8:**
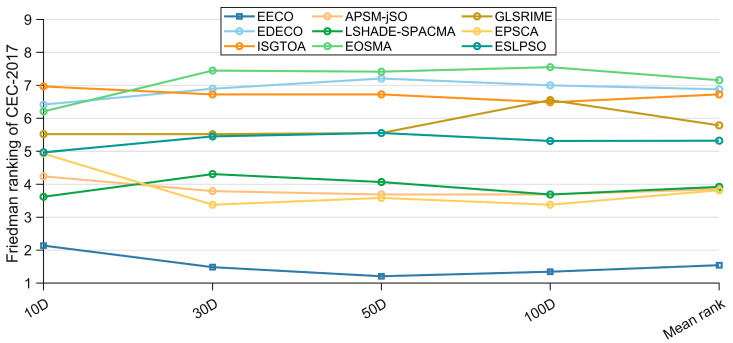
Friedman rankings of EECO and other competing algorithms.

**Figure 9:**
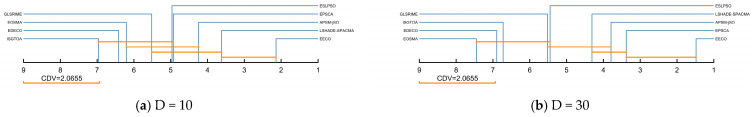

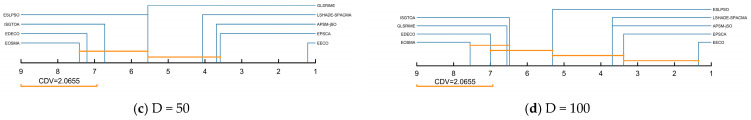
Nemenyi post hoc test of EECO and other competing algorithms.

The Friedman test results for EECO against competing algorithms on the CEC-2017 benchmark are depicted in Figure 8. Across all dimensional settings, EECO consistently secures the first position, attaining mean ranks of 2.138, 1.483, 1.207, and 1.345 for 10D, 30D, 50D, and 100D, respectively. The *p*-values reported in the last column of Table 8 confirm the presence of statistically significant differences among the algorithms. To quantify the magnitude of these differences, a Nemenyi post hoc analysis—described in Section 4.3—is applied, with the outcomes illustrated in Figure 9. On 10D functions, no significant difference is detected between EECO and LSHADE-SPACMA; likewise, EECO and EPSCA are statistically indistinguishable on 50-D and 100-D functions. In all remaining pairwise comparisons, the CDV segments do not connect EECO to any competing algorithm, indicating that EECO is significantly superior to those counterparts.

**Figure 10:**
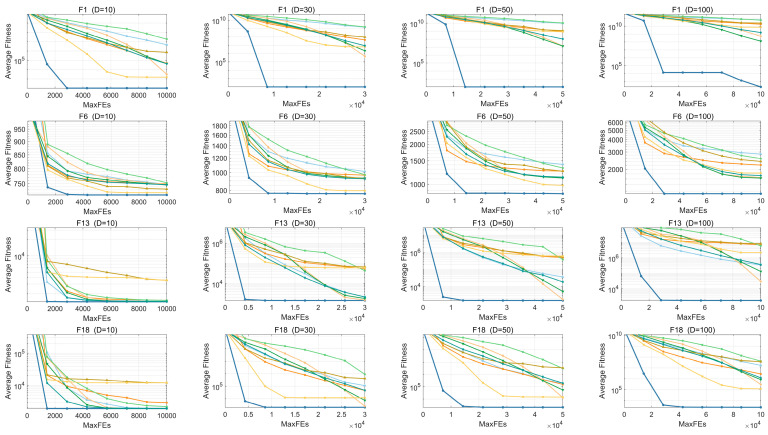

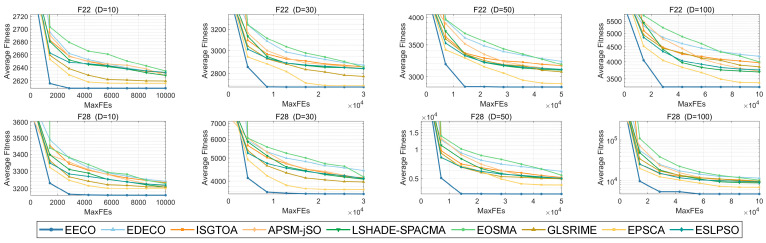
Convergence curves of EECO and other competing algorithms.

Figure 10 illustrates the convergence profiles of EECO alongside the competing algorithms. Convergence curves are indispensable for tracking the optimization trajectory, as they allow direct inspection of both convergence speed and final accuracy, thereby exposing common pitfalls such as premature stagnation or oscillatory behavior. By visualizing the evolution of fitness values, researchers can appraise the efficiency of each method and make informed parameter adjustments to enhance performance. These plots also serve as diagnostic instruments for assessing how well an algorithm adapts to varying problem complexities, rendering them an integral component of algorithmic design and performance evaluation. In this subsection, convergence curves are provided for six representative test functions—unimodal F1, multimodal F6, hybrid F13 and F18, and composite F22 and F28—where the x-axis denotes the number of fitness evaluations and the y-axis presents the corresponding fitness values.

The results indicate that the EECO algorithm exhibits clear advantages in these functions, achieving fast convergence and maintaining the lowest objective values among the compared algorithms. Even in the more challenging hybrid and combinatorial functions, the EECO algorithm consistently delivers competitive or superior optimization results, reflecting its robustness across a wide range of problem environments. The excellent convergence efficiency of the EECO algorithm on unimodal functions is attributed to the further exploitation of the optimal solution by the PM strategy. The RPS creatively resets part of the population, which improves the quality of the population and facilitates the finding of more promising regions, which in turn enhances the exploration capability of the EECO algorithm. This is the reason why the EECO algorithm performs well on multimodal functions. The TDUF allows each individual to choose a strategy that favors them based on the search process, achieving a balance between exploitation and exploration. Hybrid and composite functions examine the balance of an algorithm, and TDUF improves the performance of the EECO algorithm on complex functions. The covariance matrix of RPS fits the direction of population evolution better, which also enhances the performance of the EECO algorithm on hybrid and composite functions.

**Figure 11:**
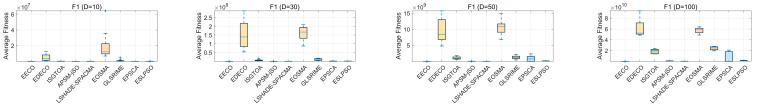

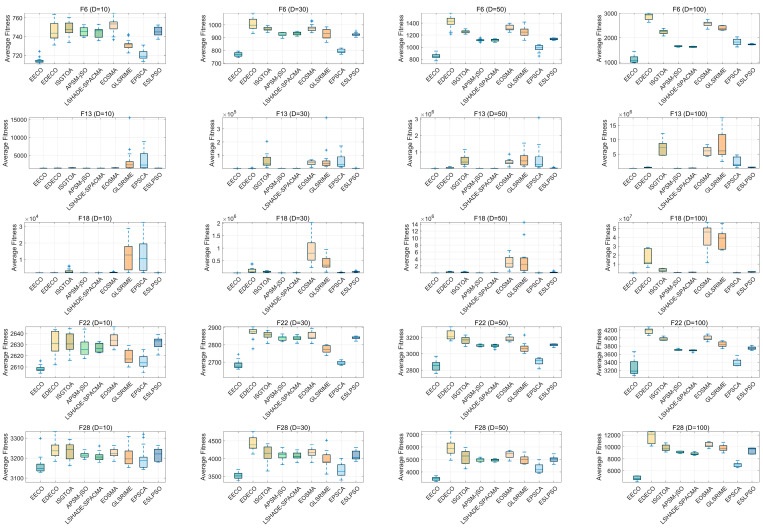
Boxplots of EECO and other competing algorithms.

Figure 11 provides the distribution of solutions provided by the EECO algorithms and the comparison algorithms in the face of the CEC-2017 test set, represented by a box plot. The box plots can show median, interquartile range (IQR), and outliers simultaneously, reflecting the concentration trend, dispersion, and extreme performance of each algorithm. The outliers and box lengths quickly identify whether an algorithm is experiencing performance fluctuations or crashes, and are more resistant to outlier interference than the mean alone. In the box plot, the lower and narrower the box as a whole indicates that the algorithm converges with high accuracy and stability. In this subsection, box plots are drawn for the six test functions (unimodal F1, multimodal F6, hybrid F13 and F18, and composite F22 and F28) where the x-axis denotes each algorithm and the y-axis denotes the corresponding fitness values. It is evident from Figure 11 that the EECO algorithm shows more robustness and stability compared to its competitors. Figure 8 clearly reveals that EECO exhibits superior robustness and stability relative to its competitors, demonstrating that the proposed enhancements bolster performance without compromising reliability. The EECO algorithm shows less fluctuation in different box plots which validates its stable performance.

Additionally, to examine the impact of different population sizes, we compare the EECO algorithm with the competing algorithms on 30-D functions in this subsection. The population sizes of the competing algorithms are set according to their original papers, and the results are presented in Table 9.

According to Table 9, EECO achieves the best Friedman rank of 1.414, whereas the original ECO algorithm ranks last with a Friedman score of 8.241. Regardless of whether the same population size or the size recommended in the original literature is used, EECO’s advantage remains clear. The Wilcoxon rank sum test shows that EECO’s performance gap versus EDECO and GLSRIME is unchanged, its margin over APSM-jSO and LSHADE-SPACMA has widened, and its lead over ISGTOA, EOSMA, EPSCA, and ESLPSO has narrowed slightly.

Collectively, the experimental evidence demonstrates that EECO comprehensively surpasses the compared methods on the CEC-2017 test suite, thereby exhibiting high reliability and exceptional adaptability in addressing global optimization problems.

## 5. Numerical Experiments Using Constrained Engineering Optimization Problems

This section evaluates EECO’s capability to address real-world engineering challenges by applying it to ten well-established constrained optimization problems, detailed in Table 10. Since metaheuristic algorithms were initially designed to solve unconstrained optimization problems, it is necessary to transform constrained optimization problems into unconstrained optimization problems. A common constraint handling method is the penalty function method. Specifically, during fitness evaluation, any candidate solution that violates one or more constraints is penalized by adding a penalty term to its objective value, thereby ensuring that it is progressively discarded owing to its elevated fitness in subsequent iterations.

**Figure 12:**
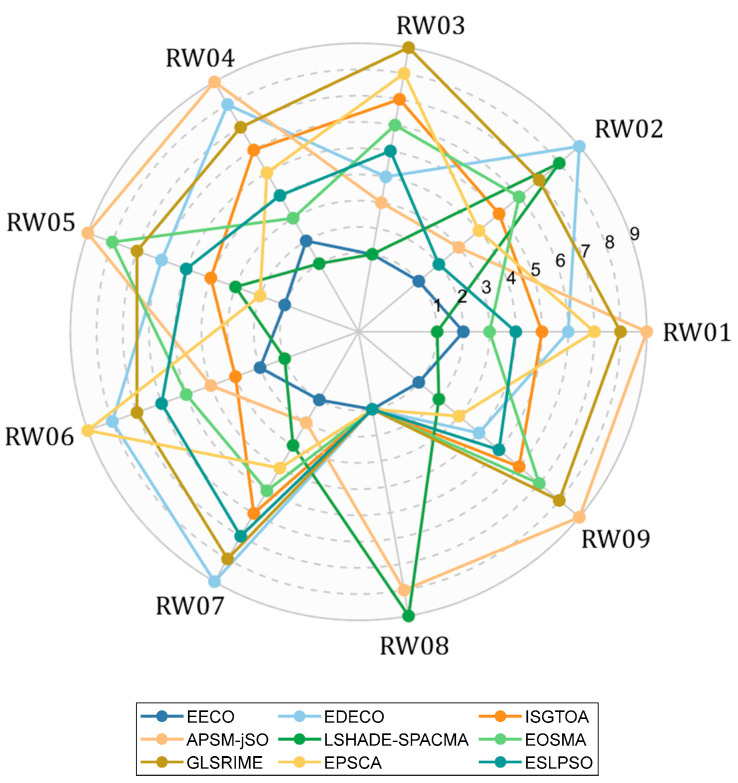
Algorithm performance sorting radar chart of EECO and other competing algorithms.

Table 11 provides a comprehensive comparison between EECO and the competing algorithms, detailing each method’s Friedman rank and the outcomes of Wilcoxon pairwise contrasts with EECO. Figure 12 complements this by presenting a radar chart of the rankings across all functions; the area enclosed by each algorithm’s polygon inversely quantifies its relative performance, with smaller areas denoting superior efficacy. According to Table 11, the EECO algorithm stands out with a Friedman ranking of 1.722. In Figure 12, the EECO algorithm encloses the smallest surface, reflecting its robustness and efficiency on engineering constrained optimization problems. The number of “+/=/−” reveals the superior performance of the EECO algorithm compared to other algorithms on the 10 engineering constraint optimization problems. Although individual algorithms outperform the EECO algorithm on some problems, most of the results show the strong competitiveness of the EECO algorithm. In conclusion, the performance of the EECO algorithm in 10 engineering constrained optimization problems is good, and these results further validate the broad application prospects of the EECO algorithm in various engineering and scientific real-world problems, making it one of the core optimization tools in the field.

## 6. Conclusions

In this work, we propose an enhanced variant of the Educational Competition Optimizer—termed EECO—which augments the original ECO algorithm by synergistically integrating three mechanisms: a regenerative population strategy, the Powell mechanism, and a trend-driven update framework, thereby substantially strengthening its search capability. The regenerative population strategy elevates population quality and markedly enhances the global exploration capability of the ECO algorithm. The Powell mechanism refines the local search procedure, contributing to further gains in convergence efficiency. The trend-driven update framework dynamically balances exploitation and exploration throughout the search process. To evaluate the effectiveness of the proposed EECO, comprehensive and extensive experiments were conducted. First, optimal parameter settings were systematically investigated on the CEC-2017 benchmark. Second, ablation studies confirmed that each enhancement contributes positively to performance and that the algorithm scales well. Comparative assessments against a suite of state-of-the-art metaheuristics further corroborate EECO’s superior efficacy. Finally, successful application to real-world constrained engineering problems underscores its practical utility. Despite its demonstrated strengths, the EECO algorithm still exhibits limitations that warrant further investigation. First, the parameter space examined in this study is restricted; more extensive and fine-grained parameter analyses are required. Second, the Powell mechanism incurs additional computational expense in high-dimensional problems, necessitating a more sophisticated activation criterion than the current iteration-based trigger. Finally, the strategy-selection mechanism itself remains amenable to refinement to mitigate mis-selections induced by premature convergence.

Looking ahead, we will pursue three complementary lines of research. First, we will develop advanced regenerative-population strategies that replace the current random selection of individuals for regeneration with adaptive, quality-aware rules—an extension expected to further strengthen algorithmic performance. Second, we will engineer a binary variant of the EECO algorithm to address a broad class of discrete optimization problems. Thirdly, we will investigate the theoretical convergence properties of the EECO algorithm, rather than relying solely on convergence curves [60]. Finally, we will integrate EECO with cutting-edge techniques from artificial intelligence, such as reinforcement-learning-based selection of search mechanisms [61], large-language-model-assisted design of improved search operators [62], and the incorporation of fuzzy-set theory for handling uncertainty [63].

## Figures and Tables

**Table 1:** Detailed description of CEC2017 test functions.

Category	No.	Function Name	Fmin
Unimodal functions	F1	Shifted and Rotated Bent Cigar Function	100
	F2	Shifted and Rotated Zakharov Function	300
Multimodal functions	F3	Shifted and Rotated Rosenbrock’s Function	400
	F4	Shifted and Rotated Rastrigin’s Function	500
	F5	Shifted and Rotated Expanded Schaffer’s Function	600
	F6	Shifted and Rotated Lunacek Bi_Rastrigin Function	700
	F7	Shifted and Rotated Non-Continuous Rastrigin’s Function	800
	F8	Shifted and Rotated Levy Function	900
	F9	Shifted and Rotated Schwefel’s Function	1000
Hybrid functions	F10	Hybrid Function 1 (N = 3)	1100
	F11	Hybrid Function 2 (N = 3)	1200
	F12	Hybrid Function 3 (N = 3)	1300
	F13	Hybrid Function 4 (N = 4)	1400
	F14	Hybrid Function 5 (N = 4)	1500
	F15	Hybrid Function 6 (N = 4)	1600
	F16	Hybrid Function 6 (N = 5)	1700
	F17	Hybrid Function 6 (N = 5)	1800
	F18	Hybrid Function 6 (N = 5)	1900
	F19	Hybrid Function 6 (N = 6)	2000
Composition functions	F20	Composition Function 1 (N = 3)	2100
	F21	Composition Function 2 (N = 3)	2200
	F22	Composition Function 3 (N = 4)	2300
	F23	Composition Function 4 (N = 4)	2400
	F24	Composition Function 5 (N = 5)	2500
	F25	Composition Function 6 (N = 5)	2600
	F26	Composition Function 7 (N = 6)	2700
	F27	Composition Function 8 (N = 6)	2800
	F28	Composition Function 9 (N = 3)	2900
	F29	Composition Function 10 (N = 3)	3000
Search Range:${\left[-100,100\right]}^{D}$; Dimension: 10/30/50/100			

**Table 2:** Details of EECO variants with different strategies.

Algorithm	ECO-R	ECO-P	ECO-T	ECO-RP	ECO-RT	ECO-PT	EECO
RPS	Y	N	N	Y	Y	N	Y
PM	N	Y	N	Y	N	Y	Y
TDUF	N	N	Y	N	Y	Y	Y

**Table 3:** Friedman test results obtained by EECO with different strategies.

Test Suite	Dimension	ECO	ECO-R	ECO-P	ECO-T	ECO-RP	ECO-RT	ECO-PT	EECO	*p*-Value
CEC-2017	10	7.414	4.207	4.621	6.448	3.207	4.379	3.983	1.741	5.80E-20
	30	7.931	4.759	5.241	6.690	2.828	3.655	3.724	1.172	1.79E-30
	50	7.828	5.207	5.517	6.483	3.069	3.103	3.552	1.241	3.74E-30
	100	7.724	5.138	5.759	6.414	3.310	3.207	3.310	1.138	5.96E-30
Mean rank		7.724	4.828	5.284	6.509	3.103	3.586	3.642	1.323	N/A
Overall rank		8	5	6	7	2	3	4	1	N/A

**Table 4:** Wilcoxon rank sum test results of EECO with different strategies.

vs. ECO +/=/-	CEC-2017 Test Suite			
	10D	30D	50D	100D
ECO-R	23/6/0	28/1/0	24/5/0	20/9/0
ECO-P	25/2/2	26/2/1	22/6/1	20/9/0
ECO-T	12/17/0	23/6/0	21/8/0	17/12/0
ECO-RP	24/5/0	28/1/0	26/3/0	24/5/0
ECO-RT	21/8/0	27/2/0	28/1/0	24/5/0
ECO-PT	22/7/0	28/1/0	28/1/0	26/3/0
EECO	28/1/0	29/0/0	29/0/0	28/1/0

**Table 5:** Experimental result of CO.

CO	Index	EECO		ECO	
		D = 30	D = 100	D = 30	D = 100
c= (0, 0, …,0)	Best	0.0000E+00	0.0000E+00	6.6613E-16	0.0000E+00
	Mean	0.0000E+00	0.0000E+00	2.8759E-02	8.5974E-03
	Std	0.0000E+00	0.0000E+00	8.8467E-02	3.2676E-02
	Rank	1	1	2	2
c= (25, 25, …, 25)	Best	0.0000E+00	0.0000E+00	1.2579E-01	5.9336E-02
	Mean	3.2016E-03	2.7909E-03	4.8354E-01	3.3116E-01
	Std	8.7753E-03	7.5325E-03	2.4722E-01	1.9795E-01
	Rank	1	1	2	2
c= (50, 50, …, 50)	Best	0.0000E+00	0.0000E+00	1.3246E-02	8.6688E-02
	Mean	1.2322E-03	3.2029E-03	4.3004E-01	2.8875E-01
	Std	3.2837E-03	9.3339E-03	2.3363E-01	1.5740E-01
	Rank	1	1	2	2
c= (75, 75, …, 75)	Best	0.0000E+00	0.0000E+00	3.7604E-03	6.4040E-02
	Mean	3.2031E-03	2.4632E-03	3.5626E-01	4.0825E-01
	Std	7.6592E-03	5.1712E-03	2.3738E-01	1.9091E-01
	Rank	1	1	2	2
c= (100, 100, …, 100)	Best	0.0000E+00	0.0000E+00	0.0000E+00	0.0000E+00
	Mean	0.0000E+00	0.0000E+00	0.0000E+00	0.0000E+00
	Std	0.0000E+00	0.0000E+00	0.0000E+00	0.0000E+00
	Rank	1	1	1	1

**Table 6:** Parameter settings of EECO and other algorithms.

**Algorithm**	**Parameter Settings**
EECO	$H=0.5,G_{1}=0.2,G_{2}=0.1,a=0.8$
ECO	$H=0.5,G_{1}=0.2,G_{2}=0.1$
EDECO	$H=0.5,G_{1}=0.2,G_{2}=0.1,\alpha =10,\beta =0.4$
ISGTOA	Ar=2N
APSM-jSO	k=3,F=0.3,cr=0.8,h=6,a=1.3
LSHADE-SPACMA	p=0.11,a=1.4,m=5,f=0.5
EOSMA	$a_{1}=2,a_{2}=1,GP=0.5,z=0.6,q=0.2$
GLSRIME	w=5,Cmax=250,α=30
EPSCA	a=2,β=0.5,c=0.5,d=1.2
ESLPSO	CR=0.5,F=0.5,c=0.1,M=N

**Table 7:** Wilcoxon rank sum test results of EECO and other competing algorithms.

EECO vs. +/=/-	CEC-2017 Test Suite			
	10D	30D	50D	100D
EDECO	25/4/0	29/0/0	29/0/0	29/0/0
ISGTOA	25/4/0	29/0/0	29/0/0	28/1/0
APSM-jSO	23/4/2	26/0/3	27/0/2	24/3/2
LSHADE-SPACMA	23/4/2	26/1/2	27/0/2	24/3/2
EOSMA	23/4/2	29/0/0	29/0/0	29/0/0
GLSRIME	23/5/1	28/1/0	29/0/0	27/2/0
EPSCA	21/8/0	25/2/2	23/6/0	21/8/0
ESLPSO	24/4/1	26/1/2	27/1/1	24/3/2

**Table 8:** Friedman test results of EECO and other competing algorithms.

Test Suite	Dimension	EECO	EDECO	ISGTOA	APSM-jSO	LSHADE-SPACMA	EOSMA	GLSRIME	EPSCA	ESLPSO	*p*-Value
CEC-2017	10	2.138	6.414	6.966	4.241	3.621	6.207	5.517	4.931	4.966	3.71E-12
	30	1.483	6.897	6.724	3.793	4.310	7.448	5.517	3.379	5.448	2.38E-21
	50	1.207	7.207	6.724	3.690	4.069	7.414	5.552	3.586	5.552	5.62E-24
	100	1.345	7.000	6.483	3.690	3.690	7.552	6.552	3.379	5.310	4.35E-25
Mean rank		1.543	6.879	6.724	3.853	3.922	7.155	5.784	3.819	5.319	N/A
Overall rank		1	8	7	3	4	9	6	2	5	N/A

**Table 9:** Experiments comparing EECO with other competing algorithms using corresponding population size.

No.	Index	EECO	ECO	EDECO	ISGTOA	APSM-jSO	LSHADE-SPACMA	EOSMA	GLSRIME	EPSCA	ESLPSO
F1	Best	1.0002E+02	1.0666E+07	5.3039E+08	1.1882E+02	8.5243E+06	1.0086E+07	3.1477E+07	3.2824E+05	1.0000E+02	1.8355E+02
	Avg	3.4633E+02	1.5081E+08	1.5110E+09	2.7454E+03	1.4707E+07	1.3942E+07	4.5786E+07	7.0851E+05	1.0000E+02	7.2423E+03
	Std	9.5038E+02	1.7324E+08	7.3613E+08	3.2596E+03	4.2702E+06	2.0713E+06	1.4076E+07	4.0758E+05	1.0205E-09	7.3898E+03
	Rank	2	9	10	3	7	6	8	5	1	4
F2	Best	3.0000E+02	3.1406E+04	1.2279E+04	2.0085E+04	5.2335E+03	4.0713E+03	3.0281E+04	5.7146E+03	3.0000E+02	3.4836E+02
	Avg	3.0000E+02	4.5059E+04	2.3419E+04	3.3115E+04	1.1271E+04	3.1166E+04	4.1500E+04	1.6717E+04	3.0000E+02	2.3970E+03
	Std	4.7476E-05	1.0953E+04	8.0982E+03	8.5638E+03	3.4608E+03	2.4188E+04	7.4724E+03	7.4384E+03	6.7928E-03	2.3566E+03
	Rank	1	10	6	8	4	7	9	5	2	3
F3	Best	4.0000E+02	5.0544E+02	5.9254E+02	4.6679E+02	4.9713E+02	4.9279E+02	4.9771E+02	4.7852E+02	4.0026E+02	4.6876E+02
	Avg	4.0159E+02	5.5680E+02	6.9028E+02	5.0745E+02	5.1675E+02	4.9829E+02	5.3460E+02	5.2175E+02	4.6569E+02	4.9488E+02
	Std	2.0216E+00	2.9201E+01	7.6803E+01	2.4864E+01	1.1539E+01	3.9403E+00	1.5220E+01	3.6552E+01	4.7785E+01	1.6021E+01
	Rank	1	9	10	5	6	4	8	7	2	3
F4	Best	5.2587E+02	6.2510E+02	6.4144E+02	5.6966E+02	6.7318E+02	6.6488E+02	5.9708E+02	5.3707E+02	5.5373E+02	5.3781E+02
	Avg	5.4391E+02	6.9282E+02	7.1821E+02	5.9683E+02	6.9967E+02	6.9119E+02	6.2902E+02	5.8326E+02	6.0351E+02	5.6481E+02
	Std	1.1072E+01	4.2995E+01	3.1051E+01	2.1147E+01	1.5795E+01	9.5705E+00	1.4125E+01	2.5505E+01	3.4847E+01	2.3486E+01
	Rank	1	8	10	4	9	7	6	3	5	2
F5	Best	6.0083E+02	6.3012E+02	6.1833E+02	6.0153E+02	6.0339E+02	6.0431E+02	6.0251E+02	6.0335E+02	6.0025E+02	6.0001E+02
	Avg	6.0263E+02	6.4769E+02	6.3018E+02	6.0811E+02	6.0450E+02	6.0492E+02	6.0482E+02	6.0923E+02	6.1175E+02	6.0079E+02
	Std	1.4591E+00	1.0315E+01	7.1418E+00	4.7916E+00	7.0646E-01	3.5636E-01	1.4715E+00	3.3493E+00	1.4140E+01	1.1799E+00
	Rank	2	10	9	6	3	5	4	7	8	1
F6	Best	7.5799E+02	9.0473E+02	9.3163E+02	8.0197E+02	9.2221E+02	8.8672E+02	8.5339E+02	8.0653E+02	7.9249E+02	7.7706E+02
	Avg	7.8412E+02	1.0923E+03	1.0130E+03	8.3930E+02	9.3361E+02	9.3261E+02	8.8058E+02	8.4485E+02	8.5611E+02	8.0191E+02
	Std	2.0643E+01	1.2298E+02	4.9454E+01	3.2468E+01	8.7529E+00	1.6240E+01	1.5305E+01	2.2496E+01	7.2626E+01	1.7981E+01
	Rank	1	10	9	3	8	7	6	4	5	2
F7	Best	8.1691E+02	8.9381E+02	9.0226E+02	8.4975E+02	9.8314E+02	9.6879E+02	8.8151E+02	8.5115E+02	8.4490E+02	8.3781E+02
	Avg	8.3854E+02	9.4892E+02	9.8250E+02	8.8300E+02	9.9831E+02	9.9988E+02	9.1234E+02	8.8550E+02	8.9363E+02	8.6245E+02
	Std	1.5512E+01	2.6660E+01	3.4335E+01	1.7928E+01	9.9401E+00	1.2985E+01	1.9471E+01	1.7335E+01	3.1775E+01	2.0222E+01
	Rank	1	7	8	3	9	10	6	4	5	2
F8	Best	9.1509E+02	3.4198E+03	1.8289E+03	1.0029E+03	9.2447E+02	9.2019E+02	9.3383E+02	1.0287E+03	9.5510E+02	9.0709E+02
	Avg	9.6117E+02	4.9803E+03	3.7534E+03	1.3566E+03	9.3527E+02	9.5019E+02	1.0403E+03	2.8395E+03	2.4155E+03	9.6526E+02
	Std	4.7488E+01	1.2327E+03	1.4570E+03	3.7236E+02	9.9517E+00	1.2270E+01	7.6847E+01	1.7226E+03	1.4781E+03	5.9133E+01
	Rank	3	10	9	6	1	2	5	8	7	4
F9	Best	2.8773E+03	4.5867E+03	6.5101E+03	4.4384E+03	8.6932E+03	7.8571E+03	6.8543E+03	3.8793E+03	4.0528E+03	3.8480E+03
	Avg	4.7690E+03	5.7015E+03	7.4975E+03	7.0771E+03	8.8536E+03	8.5292E+03	7.3926E+03	4.5379E+03	5.1015E+03	4.9839E+03
	Std	7.7122E+02	5.5244E+02	6.6318E+02	1.2329E+03	1.2304E+02	3.7494E+02	2.5974E+02	5.5227E+02	7.6133E+02	6.6182E+02
	Rank	2	5	8	6	10	9	7	1	4	3
F10	Best	1.1289E+03	1.2634E+03	1.2920E+03	1.1614E+03	1.2516E+03	1.2617E+03	1.2885E+03	1.2132E+03	1.1119E+03	1145.830
	Avg	1.1809E+03	1.4149E+03	1.4184E+03	1.2295E+03	1.2954E+03	1.2994E+03	1.3285E+03	1.2898E+03	1.1881E+03	1.2416E+03
	Std	5.2312E+01	1.3138E+02	8.9162E+01	4.8233E+01	2.3060E+01	2.4760E+01	2.4552E+01	5.0144E+01	4.8642E+01	51.81430709
	Rank	1	9	10	3	6	7	8	5	2	4
F11	Best	2.1217E+03	2.2676E+06	7.8723E+06	3.3165E+04	8.1508E+05	1.0060E+06	1.9866E+06	1.5908E+06	2.6860E+03	20253.4945
	Avg	3.4859E+03	8.7382E+06	3.5060E+07	4.8977E+05	2.8398E+06	2.0884E+06	4.4211E+06	5.7738E+06	2.2353E+04	184248.2761
	Std	1.5356E+03	4.3976E+06	2.4954E+07	4.2222E+05	1.0363E+06	4.8450E+05	1.9355E+06	4.2418E+06	2.7735E+04	137801.6784
	Rank	1	9	10	4	6	5	7	8	2	3
F12	Best	1.4342E+03	1.5840E+04	6.8309E+04	1.5642E+03	1.0454E+05	1.9293E+05	9.3801E+04	8.9797E+03	1.3293E+03	1922.892453
	Avg	1.9688E+03	1.5862E+05	1.7408E+05	1.4528E+04	3.7133E+05	4.9712E+05	2.8988E+05	3.9260E+04	1.3987E+03	16527.31732
	Std	4.1827E+02	3.1803E+05	6.6446E+04	1.5802E+04	1.3717E+05	2.0112E+05	1.1831E+05	2.6510E+04	1.0609E+02	19690.11727
	Rank	2	6	7	3	9	10	8	5	1	4
F13	Best	1.4788E+03	2.7886E+03	1.6480E+03	1.6203E+03	1.7979E+03	2.1512E+03	1.5739E+03	7.6255E+03	1.5117E+03	1524.451052
	Avg	1.5135E+03	5.3163E+04	2.1678E+03	3.2955E+03	1.9475E+03	2.8751E+03	2.5317E+03	4.2317E+04	8.6166E+04	2105.770277
	Std	5.3282E+01	6.3146E+04	1.0257E+03	3.3811E+03	1.3626E+02	6.2659E+02	7.9175E+02	2.8415E+04	2.2787E+05	1268.647702
	Rank	1	9	4	7	2	6	5	8	10	3
F14	Best	1.5815E+03	2.2874E+03	1.1776E+04	1.6442E+03	1.6452E+04	3.5940E+04	1.8829E+04	3.6940E+03	1.5094E+03	1762.681814
	Avg	1.7570E+03	1.4454E+04	2.9877E+04	8.3079E+03	3.5583E+04	6.3446E+04	4.2947E+04	1.5308E+04	1.5630E+03	7615.436605
	Std	8.2357E+01	1.1454E+04	1.2842E+04	8.3423E+03	1.2685E+04	2.1543E+04	1.6884E+04	1.2291E+04	4.6956E+01	10029.45177
	Rank	2	5	7	4	8	10	9	6	1	3
F15	Best	1.6169E+03	2.3499E+03	2.3269E+03	2.0244E+03	3.0114E+03	3.0242E+03	2.2652E+03	2.2036E+03	1.9875E+03	1902.168898
	Avg	2.0055E+03	2.8809E+03	3.1450E+03	2.4159E+03	3.4310E+03	3.2715E+03	2.6994E+03	2.6655E+03	2.6732E+03	2421.05883
	Std	2.3294E+02	3.4811E+02	2.9881E+02	2.3151E+02	2.0254E+02	1.5112E+02	2.4123E+02	2.3800E+02	4.1118E+02	319.4655107
	Rank	1	7	8	2	10	9	6	4	5	3
F16	Best	1.7666E+03	1.8397E+03	2.0277E+03	1.8101E+03	2.2746E+03	2.0383E+03	1.8866E+03	1.8106E+03	1.7515E+03	1776.714515
	Avg	1.8163E+03	2.2241E+03	2.2589E+03	2.0566E+03	2.4474E+03	2.3388E+03	2.0499E+03	2.1582E+03	2.2758E+03	1989.755998
	Std	3.4497E+01	2.8303E+02	1.5197E+02	1.4830E+02	7.4347E+01	1.3313E+02	9.9962E+01	2.4964E+02	2.9003E+02	146.1895127
	Rank	1	6	7	4	10	9	3	5	8	2
F17	Best	1.8504E+03	4.5316E+04	2.2311E+04	2.4691E+04	2.6405E+04	3.1821E+04	7.7773E+04	1.4108E+05	3.3309E+03	23394.46988
	Avg	2.0062E+03	7.7655E+05	8.2441E+04	1.3067E+05	4.0943E+04	6.6430E+04	1.2170E+05	5.9382E+05	6.9747E+04	85853.49497
	Std	9.5558E+01	1.3497E+06	4.2900E+04	1.9036E+05	1.1788E+04	2.1851E+04	3.4056E+04	3.6511E+05	6.7148E+04	72715.98745
	Rank	1	10	5	8	2	3	7	9	4	6
F18	Best	1.9656E+03	3.0807E+03	8.8543E+03	2.1070E+03	1.4125E+04	1.5740E+04	1.2324E+04	3.3755E+03	1.9115E+03	2056.546423
	Avg	2.0277E+03	1.4517E+04	1.1076E+05	9.5544E+03	2.8552E+04	4.7673E+04	4.8710E+04	1.6240E+04	1.9767E+03	8211.842954
	Std	4.5265E+01	1.2393E+04	1.1407E+05	7.3783E+03	1.0754E+04	2.1625E+04	2.6514E+04	1.3260E+04	1.0889E+02	8545.087761
	Rank	2	5	10	4	7	8	9	6	1	3
F19	Best	2.0409E+03	2.3207E+03	2.3527E+03	2.1026E+03	2.6670E+03	2.6476E+03	2.2792E+03	2.2145E+03	2.0717E+03	2063.273556
	Avg	2.2514E+03	2.6051E+03	2.6169E+03	2.3633E+03	2.8630E+03	2.8472E+03	2.4410E+03	2.5035E+03	2.4774E+03	2342.110457
	Std	1.5766E+02	1.5900E+02	1.4438E+02	1.8792E+02	1.1100E+02	8.0219E+01	9.6795E+01	2.1337E+02	2.2810E+02	198.9296812
	Rank	1	7	8	3	10	9	4	6	5	2
F20	Best	2.3110E+03	2.2275E+03	2.4240E+03	2.3508E+03	2.4849E+03	2.4592E+03	2.3883E+03	2.3612E+03	2.3512E+03	2343.067091
	Avg	2.3397E+03	2.4474E+03	2.4809E+03	2.3722E+03	2.4995E+03	2.4919E+03	2.4102E+03	2.4096E+03	2.3841E+03	2358.622466
	Std	1.6298E+01	8.4701E+01	2.9743E+01	1.5172E+01	7.9571E+00	1.3082E+01	1.4954E+01	2.8969E+01	2.2133E+01	11.6527675
	Rank	1	7	8	3	10	9	6	5	4	2
F21	Best	2.3000E+03	2.3412E+03	2.6673E+03	2.3000E+03	2.3304E+03	2.3257E+03	2.3274E+03	2.3059E+03	2.3025E+03	2300
	Avg	2.5296E+03	3.7563E+03	6.1441E+03	2.6134E+03	2.3403E+03	2.3315E+03	2.3409E+03	5.2254E+03	6.2178E+03	3351.609735
	Std	8.8529E+02	2.3015E+03	3.0103E+03	1.2087E+03	5.5704E+00	4.8164E+00	7.6173E+00	1.5905E+03	1.7721E+03	1818.548534
	Rank	4	7	9	5	2	1	3	8	10	6
F22	Best	2.6757E+03	2.7639E+03	2.7771E+03	2.7017E+03	2.8356E+03	2.8313E+03	2.7430E+03	2.6965E+03	2.7038E+03	2685.358794
	Avg	2.6987E+03	2.8736E+03	2.8699E+03	2.7518E+03	2.8587E+03	2.8516E+03	2.7712E+03	2.7699E+03	2.7382E+03	2714.712244
	Std	1.7032E+01	5.8767E+01	3.3452E+01	3.2589E+01	1.1832E+01	9.3416E+00	1.4915E+01	4.6313E+01	2.6602E+01	24.31337661
	Rank	1	10	9	4	8	7	6	5	3	2
F23	Best	2.8463E+03	2.9565E+03	2.9641E+03	2.8666E+03	2.9957E+03	2.9683E+03	2.8739E+03	2.8882E+03	2.8704E+03	2854.535314
	Avg	2.8840E+03	3.0611E+03	3.0319E+03	2.9007E+03	3.0187E+03	3.0110E+03	2.9244E+03	2.9229E+03	2.9246E+03	2890.809262
	Std	3.5917E+01	7.9901E+01	3.7227E+01	2.7112E+01	1.0942E+01	1.2909E+01	1.9006E+01	1.7063E+01	4.9960E+01	35.53460198
	Rank	1	10	9	3	8	7	5	4	6	2
F24	Best	2.8753E+03	2.9186E+03	2.9742E+03	2.8873E+03	2.8923E+03	2.8892E+03	2.8951E+03	2.8878E+03	2.8835E+03	2883.492632
	Avg	2.8798E+03	2.9744E+03	3.0436E+03	2.9038E+03	2.9010E+03	2.8920E+03	2.9127E+03	2.9107E+03	2.8881E+03	2890.814913
	Std	5.2929E+00	3.3228E+01	4.8366E+01	1.9004E+01	4.8665E+00	1.8164E+00	1.4488E+01	2.4058E+01	1.0481E+01	11.16570171
	Rank	1	9	10	6	5	4	8	7	2	3
F25	Best	2.8000E+03	3.7762E+03	3.8738E+03	2.8009E+03	5.3167E+03	5.1984E+03	3.6112E+03	2.9033E+03	4.3040E+03	3886.286574
	Avg	4.1081E+03	5.7975E+03	5.4866E+03	4.5041E+03	5.5753E+03	5.5068E+03	4.2135E+03	4.8421E+03	4.7132E+03	4326.449876
	Std	4.4718E+02	7.6867E+02	6.8761E+02	7.8022E+02	1.2903E+02	1.3799E+02	4.5937E+02	8.7589E+02	4.1669E+02	339.4984132
	Rank	1	10	7	4	9	8	2	6	5	3
F26	Best	3.1296E+03	3.2097E+03	3.2424E+03	3.2113E+03	3.2261E+03	3.2221E+03	3.2217E+03	3.2205E+03	3.1983E+03	3203.44455
	Avg	3.1818E+03	3.2676E+03	3.2904E+03	3.2342E+03	3.2381E+03	3.2301E+03	3.2410E+03	3.2449E+03	3.2287E+03	3225.80584
	Std	1.8643E+01	3.1587E+01	4.4358E+01	1.6999E+01	6.9512E+00	3.7903E+00	1.1642E+01	2.3599E+01	1.2545E+01	15.60638933
	Rank	1	9	10	5	6	4	7	8	3	2
F27	Best	3.1000E+03	3.2530E+03	3.3173E+03	3.2234E+03	3.2516E+03	3.2389E+03	3.2297E+03	3.2230E+03	3.1000E+03	3196.515686
	Avg	3.1658E+03	3.3715E+03	3.4898E+03	3.2521E+03	3.2704E+03	3.2527E+03	3.2666E+03	3.2556E+03	3.1618E+03	3227.114407
	Std	6.6245E+01	4.9180E+01	9.1542E+01	1.7315E+01	1.3263E+01	1.0587E+01	2.0342E+01	2.0105E+01	6.9239E+01	25.1513953
	Rank	2	9	10	4	8	5	7	6	1	3
F28	Best	3.3637E+03	3.6163E+03	4.1280E+03	3.4132E+03	4.0225E+03	3.8991E+03	3.7793E+03	3.7224E+03	3.5801E+03	3408.451426
	Avg	3.5718E+03	4.4258E+03	4.4271E+03	3.7060E+03	4.2215E+03	4.0720E+03	3.9616E+03	3.9641E+03	3.8797E+03	3712.883355
	Std	1.5403E+02	4.1655E+02	1.9062E+02	1.3982E+02	1.1022E+02	9.8138E+01	1.1614E+02	1.9860E+02	2.4113E+02	198.7913483
	Rank	1	9	10	2	8	7	5	6	4	3
F29	Best	3.4220E+03	3.6234E+04	1.6772E+05	5.7725E+03	1.1301E+05	1.0762E+05	9.2716E+04	3.7939E+04	5.3648E+03	5296.087599
	Avg	7.0266E+03	3.2390E+05	1.4152E+06	1.0034E+04	2.1310E+05	1.9048E+05	4.3292E+05	2.4792E+05	7.4678E+03	8673.580642
	Std	3.8217E+03	2.6737E+05	1.2694E+06	3.2542E+03	5.8247E+04	5.1902E+04	2.1164E+05	2.3098E+05	1.8547E+03	2537.319778
	Rank	1	8	10	4	6	5	9	7	2	3
Friedman Rank		1.414	8.241	8.517	4.345	6.793	6.552	6.310	5.793	4.069	2.966
+/=/−		N/A	29/0/0	29/0/0	27/2/0	27/1/1	27/1/1	27/1/1	28/1/0	21/4/4	21/7/1

**Table 10:** Details of real-world constrained engineering optimization problems.

Problem	Name	D
RW01	Tension/compression spring design problem	3
RW02	Pressure vessel design problem	4
RW03	Three-bar truss design problem	2
RW04	Welded beam design problem	4
RW05	Speed reducer design problem	7
RW06	Gear train design problem	4
RW07	Cantilever beam design problem	5
RW08	Multiple disk clutch brake design problem	5
RW09	Step-cone pulley problem	5

**Table 11:** Experiments comparing EECO with other competing algorithms at constrained engineering optimization problems.

No.	Index	EECO	EDECO	ISGTOA	APSM-jSO	LSHADE-SPACMA	EOSMA	GLSRIME	EPSCA	ESLPSO
RW1	Best	1.2666E-02	1.2756E-02	1.2787E-02	1.7773E-02	1.2666E-02	1.2672E-02	1.3113E-02	1.2666E-02	1.2720E-02
	Mean	1.2771E-02	1.3704E-02	1.3109E-02	1.7775E-02	1.2684E-02	1.2869E-02	1.6981E-02	1.3944E-02	1.2882E-02
	Std	2.0705E-04	9.8361E-04	3.8124E-04	2.9736E-06	2.7940E-05	2.2313E-04	2.3095E-03	1.8742E-03	2.3411E-04
	Rank	2	6	5	9	1	3	8	7	4
RW2	Best	5.8504E+03	6.3582E+03	6.0758E+03	6.2707E+03	6.4653E+03	6.2087E+03	6.2595E+03	5.8728E+03	5.8770E+03
	Mean	6.0591E+03	8.6606E+03	6.6352E+03	6.4423E+03	7.5240E+03	6.8430E+03	7.1244E+03	6.4951E+03	6.3556E+03
	Std	2.3813E+02	1.2218E+03	4.8391E+02	2.7207E+02	1.8974E+03	3.6300E+02	8.8165E+02	6.2261E+02	3.7441E+02
	Rank	1	9	5	3	8	6	7	4	2
RW3	Best	2.6389E+02	2.6389E+02	2.6389E+02	2.6389E+02	2.6389E+02	2.6389E+02	2.6389E+02	2.6389E+02	2.6389E+02
	Mean	2.6389E+02	2.6389E+02	2.6391E+02	2.6389E+02	2.6389E+02	2.6390E+02	2.6466E+02	2.6393E+02	2.6389E+02
	Std	5.9918E-14	6.7365E-03	1.3135E-02	7.8658E-10	6.8317E-14	1.4456E-02	9.0553E-01	4.5358E-02	4.9570E-03
	Rank	1	4	7	3	1	6	9	8	5
RW4	Best	1.6928E+00	1.7288E+00	1.7370E+00	1.0890E+15	1.6928E+00	1.7030E+00	1.7213E+00	1.6999E+00	1.7152E+00
	Mean	1.6928E+00	2.0392E+00	1.7792E+00	1.0890E+15	1.6928E+00	1.7489E+00	1.9902E+00	1.7726E+00	1.7668E+00
	Std	6.7949E-05	2.2470E-01	3.8263E-02	8.2773E+04	6.4020E-06	3.7259E-02	1.7265E-01	1.2312E-01	7.8868E-02
	Rank	2	8	6	9	1	3	7	5	4
RW5	Best	2.8732E+03	2.9960E+03	2.9937E+03	1.2708E+07	2.9936E+03	3.0033E+03	2.9976E+03	2.9936E+03	2.9947E+03
	Mean	2.8734E+03	3.0031E+03	2.9940E+03	1.2708E+07	2.9936E+03	3.0133E+03	3.0105E+03	2.9936E+03	2.9962E+03
	Std	1.0284E-01	4.0047E+00	3.0598E-01	1.4755E-01	1.9172E-03	5.3050E+00	1.5672E+01	5.5850E-04	1.1368E+00
	Rank	1	6	4	9	3	8	7	2	5
RW6	Best	2.7009E-12	2.7009E-12	2.7009E-12	2.7009E-12	2.7009E-12	2.3078E-11	2.3078E-11	2.3078E-11	2.7009E-12
	Mean	5.0620E-10	5.2116E-09	5.8190E-10	7.9145E-10	2.7299E-10	1.1105E-09	4.2618E-09	8.0077E-09	1.1875E-09
	Std	1.0261E-09	1.2218E-08	7.2954E-10	9.0042E-10	3.8346E-10	6.3158E-10	6.0422E-09	1.3910E-08	7.9503E-10
	Rank	2	8	3	4	1	5	7	9	6
RW7	Best	1.3400E+00	1.3524E+00	1.3431E+00	1.3400E+00	1.3400E+00	1.3459E+00	1.3526E+00	1.3401E+00	1.3520E+00
	Mean	1.3400E+00	1.5506E+00	1.3635E+00	1.3400E+00	1.3405E+00	1.3536E+00	1.4338E+00	1.3430E+00	1.3756E+00
	Std	6.7764E-08	2.2988E-01	1.5331E-02	3.3567E-05	4.5479E-04	6.5782E-03	8.8386E-02	3.7741E-03	2.5766E-02
	Rank	1	9	6	2	3	5	8	4	7
RW8	Best	3.9247E+08	3.9247E+08	3.9247E+08	1.3400E+09	1.3510E+09	3.9247E+08	3.9247E+08	3.9247E+08	3.9247E+08
	Mean	3.9247E+08	3.9247E+08	3.9247E+08	1.3647E+09	1.3747E+09	3.9247E+08	3.9247E+08	3.9247E+08	3.9247E+08
	Std	6.2829E-08	6.2829E-08	6.2829E-08	7.8338E+07	7.4639E+07	6.2829E-08	6.2829E-08	6.2829E-08	6.2829E-08
	Rank	1	1	1	8	9	1	1	1	1
RW9	Best	1.6085E+01	1.6304E+01	1.6754E+01	1.0098E+11	1.6089E+01	1.6849E+01	1.6306E+01	1.6111E+01	1.6141E+01
	Mean	1.6086E+01	1.6687E+01	1.7097E+01	1.0098E+11	1.6101E+01	1.7377E+01	1.7506E+01	1.6516E+01	1.6769E+01
	Std	1.0423E-04	3.3065E-01	2.6577E-01	1.1988E+02	6.6970E-03	3.3127E-01	1.4142E+00	1.7645E-01	4.6407E-01
	Rank	1	4	6	9	2	7	8	3	5
Friedman Rank		1.722	6.444	5.111	6.222	3.278	5.222	7.222	5.111	4.667
+/=/−		N/A	7/2/0	7/2/0	8/1/0	5/3/1	7/2/0	8/1/0	6/3/0	6/3/0

## Data Availability

The data is provided within the manuscript.

## Appendix A

**Table A1:** Experiments comparing EECO with different population sizes (10D).

No.	Index	N = 5D	N = 10D	N = 15D	N = 20D	N = 25D	N = 30D
F1	Best	4.8595E+04	1.0763E+02	9.4699E+02	3.9914E+03	1.9278E+04	8.4037E+03
	Avg	1.9123E+06	6.1492E+03	1.7517E+04	4.8399E+05	4.9668E+05	7.9832E+06
	Std	4.3461E+06	7.6330E+03	4.2886E+04	7.7810E+05	5.5473E+05	9.6802E+06
	Rank	5	1	2	3	4	6
F2	Best	3.0242E+02	3.0000E+02	3.0001E+02	3.0024E+02	3.0190E+02	3.0086E+02
	Avg	3.0229E+03	4.0358E+02	3.0347E+02	7.9393E+02	3.5153E+02	3.9217E+02
	Std	3.7665E+03	2.3704E+02	1.0456E+01	1.8443E+03	1.2587E+02	1.4431E+02
	Rank	6	4	1	5	2	3
F3	Best	4.0058E+02	4.0180E+02	4.0346E+02	4.0486E+02	4.0285E+02	4.0504E+02
	Avg	4.1157E+02	4.0516E+02	4.0503E+02	4.0560E+02	4.0605E+02	4.0717E+02
	Std	2.4411E+01	1.8355E+00	9.7697E-01	6.1410E-01	1.3397E+00	2.2543E+00
	Rank	6	2	1	3	4	5
F4	Best	5.0612E+02	5.0301E+02	5.1582E+02	5.1504E+02	5.0594E+02	5.1237E+02
	Avg	5.2157E+02	5.1216E+02	5.2575E+02	5.2907E+02	5.2476E+02	5.2828E+02
	Std	8.6368E+00	6.4400E+00	5.6800E+00	7.4053E+00	7.5196E+00	9.2499E+00
	Rank	2	1	4	6	3	5
F5	Best	6.0295E+02	6.0001E+02	6.0018E+02	6.0081E+02	6.0061E+02	6.0202E+02
	Avg	6.0909E+02	6.0202E+02	6.0043E+02	6.0183E+02	6.0218E+02	6.0454E+02
	Std	4.5711E+00	3.3227E+00	3.6358E-01	7.7587E-01	1.2081E+00	1.6609E+00
	Rank	6	3	1	2	4	5
F6	Best	7.1924E+02	7.1620E+02	7.2143E+02	7.3322E+02	7.2249E+02	7.2952E+02
	Avg	7.3863E+02	7.2902E+02	7.3799E+02	7.4284E+02	7.4257E+02	7.4279E+02
	Std	1.2719E+01	1.0369E+01	7.6167E+00	5.5781E+00	8.9606E+00	1.0005E+01
	Rank	3	1	2	6	4	5
F7	Best	8.0340E+02	8.0510E+02	8.1230E+02	8.1695E+02	8.0941E+02	8.2126E+02
	Avg	8.1352E+02	8.1147E+02	8.2517E+02	8.2651E+02	8.2581E+02	8.3387E+02
	Std	6.1569E+00	5.0650E+00	5.5161E+00	7.0147E+00	9.7016E+00	8.8162E+00
	Rank	2	1	3	5	4	6
F8	Best	9.1818E+02	9.0000E+02	9.0000E+02	9.0030E+02	9.0003E+02	9.0150E+02
	Avg	1.0713E+03	9.0940E+02	9.0073E+02	9.1313E+02	9.0675E+02	9.0810E+02
	Std	1.5316E+02	2.3728E+01	1.8366E+00	4.6510E+01	6.5648E+00	9.6271E+00
	Rank	6	4	1	5	2	3
F9	Best	1.4081E+03	1.6475E+03	1.7746E+03	2.1070E+03	1.9598E+03	1.5376E+03
	Avg	2.1563E+03	2.1775E+03	2.2668E+03	2.4329E+03	2.4066E+03	2.2734E+03
	Std	4.1512E+02	3.3064E+02	2.8930E+02	2.5572E+02	2.8808E+02	3.0560E+02
	Rank	1	2	3	6	5	4
F10	Best	1.1071E+03	1.1048E+03	1.1035E+03	1.1069E+03	1.1068E+03	1.1069E+03
	Avg	1.1681E+03	1.1226E+03	1.1155E+03	1.1132E+03	1.1257E+03	1.1241E+03
	Std	5.9867E+01	1.3765E+01	1.2798E+01	4.7963E+00	4.2826E+01	1.3995E+01
	Rank	6	3	2	1	5	4
F11	Best	2.3525E+03	1.3422E+03	1.5929E+03	2.8385E+03	3.6268E+03	3.6692E+03
	Avg	1.6594E+06	5.8179E+03	4.5646E+05	1.6254E+05	2.1813E+04	1.2329E+05
	Std	5.4052E+06	8.1754E+03	1.7416E+06	5.6720E+05	1.9068E+04	2.0884E+05
	Rank	6	1	5	4	2	3
F12	Best	1.3787E+03	1.3516E+03	1.3372E+03	1.3406E+03	1.3669E+03	1.6952E+03
	Avg	2.4121E+03	1.6045E+03	1.5285E+03	1.5196E+03	1.7626E+03	3.2664E+03
	Std	1.1576E+03	3.5796E+02	2.2942E+02	2.1092E+02	2.6638E+02	2.9867E+03
	Rank	5	3	2	1	4	6
F13	Best	1.4230E+03	1.4041E+03	1.4164E+03	1.4192E+03	1.4225E+03	1.4245E+03
	Avg	1.4371E+03	1.4261E+03	1.4281E+03	1.4287E+03	1.4290E+03	1.4385E+03
	Std	1.0260E+01	9.9050E+00	6.9769E+00	3.9787E+00	4.6537E+00	7.2611E+00
	Rank	5	1	2	3	4	6
F14	Best	1.5063E+03	1.5002E+03	1.5032E+03	1.5091E+03	1.5095E+03	1.5089E+03
	Avg	1.6518E+03	1.5285E+03	1.5204E+03	1.5216E+03	1.5234E+03	1.5526E+03
	Std	1.5990E+02	1.7736E+01	1.8269E+01	1.0545E+01	9.5723E+00	4.2394E+01
	Rank	6	4	1	2	3	5
F15	Best	1.6023E+03	1.6049E+03	1.6176E+03	1.6545E+03	1.6148E+03	1.6136E+03
	Avg	1.7508E+03	1.6427E+03	1.6565E+03	1.7184E+03	1.6937E+03	1.7261E+03
	Std	2.6290E+02	5.7058E+01	3.1351E+01	5.3756E+01	7.0774E+01	7.2044E+01
	Rank	6	1	2	4	3	5
F16	Best	1.7261E+03	1.7287E+03	1.7485E+03	1.7472E+03	1.7590E+03	1.7683E+03
	Avg	1.7576E+03	1.7640E+03	1.7701E+03	1.7858E+03	1.7780E+03	1.7929E+03
	Std	4.0995E+01	1.8687E+01	1.4271E+01	2.1839E+01	1.4121E+01	2.0678E+01
	Rank	1	2	3	5	4	6
F17	Best	1.8450E+03	1.8279E+03	1.8271E+03	1.8555E+03	1.9132E+03	1.9449E+03
	Avg	6.4087E+03	1.8997E+03	1.8840E+03	1.9201E+03	2.0905E+03	3.1689E+03
	Std	7.0102E+03	8.1512E+01	8.6589E+01	7.1315E+01	1.4029E+02	2.0460E+03
	Rank	6	2	1	3	4	5
F18	Best	1.9064E+03	1.9025E+03	1.9040E+03	1.9053E+03	1.9059E+03	1.9071E+03
	Avg	2.0149E+03	1.9076E+03	1.9061E+03	1.9073E+03	1.9097E+03	1.9189E+03
	Std	3.4349E+02	5.3305E+00	2.0609E+00	1.3595E+00	2.3615E+00	9.3895E+00
	Rank	6	3	1	2	4	5
F19	Best	2.0227E+03	2.0264E+03	2.0591E+03	2.0740E+03	2.0266E+03	2.0700E+03
	Avg	2.0862E+03	2.0718E+03	2.0955E+03	2.1293E+03	2.1215E+03	2.1232E+03
	Std	4.6870E+01	2.9628E+01	3.5544E+01	3.6273E+01	4.2822E+01	4.0521E+01
	Rank	2	1	3	6	4	5
F20	Best	2.2062E+03	2.2056E+03	2.2066E+03	2.2044E+03	2.2089E+03	2.2059E+03
	Avg	2.2872E+03	2.3081E+03	2.2943E+03	2.2911E+03	2.2939E+03	2.2802E+03
	Std	4.8298E+01	2.9635E+01	4.1245E+01	5.7039E+01	4.7961E+01	5.2861E+01
	Rank	2	6	5	3	4	1
F21	Best	2.2431E+03	2.3000E+03	2.2121E+03	2.2265E+03	2.2157E+03	2.2172E+03
	Avg	2.3085E+03	2.3028E+03	2.2846E+03	2.3037E+03	2.2876E+03	2.3099E+03
	Std	2.0642E+01	3.3049E+00	3.3724E+01	2.1425E+01	3.5405E+01	2.7207E+01
	Rank	5	3	1	4	2	6
F22	Best	2.6059E+03	2.6053E+03	2.6074E+03	2.6160E+03	2.6156E+03	2.6234E+03
	Avg	2.6319E+03	2.6192E+03	2.6225E+03	2.6286E+03	2.6301E+03	2.6329E+03
	Std	2.5433E+01	2.4610E+01	1.2546E+01	7.2161E+00	1.4344E+01	7.2166E+00
	Rank	5	1	2	3	4	6
F23	Best	2.7347E+03	2.7295E+03	2.6808E+03	2.5246E+03	2.6111E+03	2.5780E+03
	Avg	2.7513E+03	2.7385E+03	2.7449E+03	2.7381E+03	2.7373E+03	2.7449E+03
	Std	1.8318E+01	7.4868E+00	1.8709E+01	6.0590E+01	4.4556E+01	5.0939E+01
	Rank	6	3	4	2	1	5
F24	Best	2.8984E+03	2.8978E+03	2.8977E+03	2.8980E+03	2.8990E+03	2.8983E+03
	Avg	2.9514E+03	2.9346E+03	2.9286E+03	2.9213E+03	2.9340E+03	2.9360E+03
	Std	4.2770E+01	2.0191E+01	3.5185E+01	3.5084E+01	1.9936E+01	1.9592E+01
	Rank	6	4	2	1	3	5
F25	Best	2.9572E+03	2.9000E+03	2.9002E+03	2.9027E+03	2.9024E+03	2.9113E+03
	Avg	3.1784E+03	2.9908E+03	2.9230E+03	2.9129E+03	2.9270E+03	2.9430E+03
	Std	1.2692E+02	1.0853E+02	3.9557E+01	1.2724E+01	2.8823E+01	2.6207E+01
	Rank	6	5	2	1	3	4
F26	Best	3.0909E+03	3.0894E+03	3.0894E+03	3.0909E+03	3.0911E+03	3.0929E+03
	Avg	3.1142E+03	3.1005E+03	3.0991E+03	3.1041E+03	3.0970E+03	3.0994E+03
	Std	2.6483E+01	1.5908E+01	1.6515E+01	2.4439E+01	4.1964E+00	7.7354E+00
	Rank	6	4	2	5	1	3
F27	Best	3.2654E+03	3.1778E+03	3.1687E+03	3.1550E+03	3.1859E+03	3.3073E+03
	Avg	3.4302E+03	3.3868E+03	3.3706E+03	3.3722E+03	3.3821E+03	3.4074E+03
	Std	6.8223E+01	8.1829E+01	9.8072E+01	8.3396E+01	6.6498E+01	3.0778E+01
	Rank	6	4	1	2	3	5
F28	Best	3.1360E+03	3.1509E+03	3.1556E+03	3.1782E+03	3.1633E+03	3.1534E+03
	Avg	3.2112E+03	3.1934E+03	3.2025E+03	3.2137E+03	3.2209E+03	3.2122E+03
	Std	6.0581E+01	3.3077E+01	3.2331E+01	2.1876E+01	3.3264E+01	3.2422E+01
	Rank	3	1	2	5	6	4
F29	Best	6.2676E+03	3.6511E+03	3.7610E+03	3.7521E+03	3.6571E+03	4.0333E+03
	Avg	4.1484E+05	3.2335E+05	6.3152E+05	5.8273E+05	2.8142E+05	3.4140E+05
	Std	3.5198E+05	4.9806E+05	1.0372E+06	1.1270E+06	4.6953E+05	5.1143E+05
	Rank	4	2	6	5	1	3

**Table A2:** Experiments comparing EECO with different population sizes (30D).

No.	Index	N = 5D	N = 10D	N = 15D	N = 20D	N = 25D	N = 30D
F1	Best	1.5604E+08	1.3040E+08	2.8527E+07	8.0355E+07	2.2776E+07	1.8340E+08
	Avg	5.8038E+08	8.7446E+08	1.0271E+09	1.5151E+09	1.1717E+09	1.0333E+09
	Std	5.0604E+08	9.6859E+08	1.7790E+09	3.7248E+09	1.5368E+09	8.7883E+08
	Rank	1	2	3	6	5	4
F2	Best	1.1317E+04	1.4841E+03	1.9051E+03	2.7909E+03	2.4060E+03	4.3986E+03
	Avg	2.3386E+04	1.0503E+04	6.8564E+03	7.6498E+03	1.3168E+04	2.0606E+04
	Std	7.4206E+03	6.1297E+03	4.0857E+03	3.4909E+03	9.7080E+03	1.1551E+04
	Rank	6	3	1	2	4	5
F3	Best	5.4119E+02	5.1888E+02	4.9059E+02	5.1834E+02	5.3091E+02	5.7505E+02
	Avg	6.1301E+02	5.8922E+02	6.0134E+02	6.6104E+02	5.7188E+02	6.3798E+02
	Std	4.6609E+01	5.0844E+01	1.6387E+02	3.3517E+02	2.2214E+01	4.7859E+01
	Rank	4	2	3	6	1	5
F4	Best	5.4283E+02	5.8542E+02	6.4876E+02	6.4931E+02	5.6765E+02	6.0920E+02
	Avg	6.1443E+02	6.3281E+02	6.8004E+02	6.9432E+02	6.7135E+02	6.9280E+02
	Std	7.9029E+01	3.0437E+01	1.9930E+01	3.1447E+01	4.2186E+01	3.6702E+01
	Rank	1	2	4	6	3	5
F5	Best	6.1392E+02	6.0406E+02	6.0684E+02	6.0732E+02	6.0933E+02	6.1060E+02
	Avg	6.2299E+02	6.1523E+02	6.1264E+02	6.1410E+02	6.1672E+02	6.1775E+02
	Std	5.0377E+00	8.1328E+00	5.4919E+00	4.0379E+00	5.5846E+00	4.3634E+00
	Rank	6	3	1	2	4	5
F6	Best	8.5511E+02	8.8794E+02	8.8462E+02	9.0581E+02	8.4331E+02	8.4672E+02
	Avg	9.9101E+02	9.7510E+02	9.2254E+02	9.4390E+02	9.5705E+02	9.5618E+02
	Std	6.8207E+01	8.3866E+01	2.8961E+01	1.7563E+01	4.3010E+01	7.8214E+01
	Rank	6	5	1	2	4	3
F7	Best	8.4591E+02	8.6841E+02	9.3928E+02	9.6960E+02	9.3160E+02	8.8010E+02
	Avg	8.8813E+02	9.3524E+02	9.8227E+02	9.9258E+02	9.9147E+02	9.8928E+02
	Std	2.5532E+01	3.9806E+01	2.3951E+01	1.6915E+01	2.9661E+01	3.6585E+01
	Rank	1	2	3	6	5	4
F8	Best	1.6977E+03	9.2417E+02	9.7128E+02	1.0698E+03	9.6668E+02	1.0217E+03
	Avg	2.5489E+03	1.5250E+03	1.2863E+03	1.5792E+03	1.4166E+03	1.6933E+03
	Std	6.2017E+02	5.3642E+02	2.7043E+02	9.1685E+02	2.9582E+02	6.9480E+02
	Rank	6	3	1	4	2	5
F9	Best	5.3758E+03	5.2068E+03	7.0759E+03	6.8100E+03	6.0362E+03	6.6660E+03
	Avg	7.0225E+03	7.6316E+03	8.0889E+03	8.1154E+03	8.1760E+03	7.9800E+03
	Std	8.6244E+02	1.0318E+03	5.5973E+02	6.6878E+02	8.0857E+02	6.8917E+02
	Rank	1	2	4	5	6	3
F10	Best	1.2490E+03	1.1509E+03	1.2037E+03	1.2156E+03	1.2694E+03	1.3064E+03
	Avg	1.3188E+03	1.2248E+03	1.2827E+03	1.3098E+03	1.3486E+03	1.4151E+03
	Std	6.7948E+01	5.2918E+01	1.0807E+02	4.4154E+01	6.3011E+01	8.5768E+01
	Rank	4	1	2	3	5	6
F11	Best	3.0107E+06	4.6772E+04	3.5878E+05	1.4575E+06	3.9047E+06	1.9433E+06
	Avg	1.0593E+07	9.9443E+06	7.7752E+06	1.0803E+07	2.0711E+07	2.7741E+07
	Std	6.7296E+06	2.1709E+07	1.5554E+07	7.0419E+06	1.7790E+07	2.5011E+07
	Rank	3	2	1	4	5	6
F12	Best	1.7214E+04	7.9647E+03	2.4240E+04	5.0770E+04	2.6545E+04	4.0316E+04
	Avg	5.4048E+04	2.1485E+04	5.4911E+04	2.7292E+05	2.8330E+05	7.4577E+05
	Std	2.7645E+04	8.1477E+03	2.0264E+04	2.6970E+05	2.7192E+05	1.4089E+06
	Rank	2	1	3	4	5	6
F13	Best	1.4808E+03	1.4743E+03	1.5180E+03	1.5915E+03	1.6267E+03	1.6892E+03
	Avg	2.0138E+03	1.5108E+03	1.5740E+03	1.7817E+03	1.9262E+03	3.9942E+03
	Std	1.6235E+03	2.5961E+01	3.4393E+01	1.4430E+02	2.1465E+02	3.0659E+03
	Rank	5	1	2	3	4	6
F14	Best	2.7911E+03	1.9056E+03	2.1998E+03	1.3694E+04	2.2146E+04	2.2301E+04
	Avg	7.9350E+03	7.1264E+03	8.8400E+03	3.6136E+04	5.2094E+04	2.4587E+05
	Std	5.0792E+03	1.1887E+04	8.6126E+03	1.9553E+04	2.4432E+04	5.6188E+05
	Rank	2	1	3	4	5	6
F15	Best	1.8317E+03	2.0407E+03	2.4629E+03	2.6047E+03	2.6467E+03	2.2475E+03
	Avg	2.1995E+03	2.4978E+03	2.9451E+03	3.0294E+03	3.1000E+03	2.9671E+03
	Std	1.9949E+02	3.0874E+02	2.8682E+02	2.2533E+02	2.7303E+02	3.3334E+02
	Rank	1	2	3	5	6	4
F16	Best	1.7740E+03	1.8379E+03	1.8650E+03	1.9674E+03	1.8358E+03	1.8888E+03
	Avg	1.8564E+03	1.9268E+03	2.1045E+03	2.1453E+03	2.1486E+03	2.1424E+03
	Std	8.2958E+01	3.8987E+01	1.8707E+02	1.2063E+02	1.5134E+02	1.9424E+02
	Rank	1	2	3	5	6	4
F17	Best	5.9045E+03	2.0245E+03	2.3376E+03	3.8728E+03	2.2364E+04	6.1198E+04
	Avg	1.5561E+05	8.5094E+03	5.4309E+03	1.7540E+04	8.9240E+04	1.3365E+05
	Std	4.7033E+05	9.7862E+03	3.2327E+03	9.5388E+03	1.1937E+05	1.1395E+05
	Rank	6	2	1	3	4	5
F18	Best	2.0851E+03	1.9795E+03	2.2630E+03	3.7107E+03	2.0000E+04	6.0360E+03
	Avg	1.3088E+04	5.4627E+03	3.7883E+03	2.9988E+04	1.4128E+05	2.5832E+05
	Std	1.2913E+04	9.4141E+03	1.7637E+03	3.7110E+04	2.5631E+05	2.8918E+05
	Rank	3	2	1	4	5	6
F19	Best	2.0986E+03	2.1835E+03	2.3893E+03	2.4547E+03	2.3678E+03	2.3740E+03
	Avg	2.2323E+03	2.4538E+03	2.6138E+03	2.6426E+03	2.6682E+03	2.6826E+03
	Std	1.1902E+02	1.8509E+02	1.2031E+02	1.3946E+02	1.7310E+02	1.3974E+02
	Rank	1	2	3	4	5	6
F20	Best	2.3508E+03	2.3851E+03	2.3928E+03	2.4391E+03	2.3831E+03	2.3854E+03
	Avg	2.3800E+03	2.4509E+03	2.4557E+03	2.4746E+03	2.4768E+03	2.4716E+03
	Std	2.1028E+01	3.6727E+01	2.8925E+01	2.0134E+01	3.5183E+01	3.8136E+01
	Rank	1	2	3	5	6	4
F21	Best	2.5081E+03	2.4322E+03	2.4519E+03	2.3823E+03	2.6968E+03	2.6734E+03
	Avg	3.3833E+03	2.9802E+03	3.7365E+03	3.5237E+03	5.6600E+03	4.8598E+03
	Std	1.3608E+03	4.8885E+02	1.4409E+03	1.7393E+03	2.3060E+03	2.8346E+03
	Rank	2	1	4	3	6	5
F22	Best	2.7286E+03	2.7267E+03	2.7939E+03	2.8104E+03	2.7720E+03	2.7831E+03
	Avg	2.7927E+03	2.8003E+03	2.8377E+03	2.8542E+03	2.8638E+03	2.8540E+03
	Std	7.3466E+01	8.7791E+01	2.8745E+01	3.2981E+01	3.6222E+01	3.4499E+01
	Rank	1	2	3	5	6	4
F23	Best	2.8550E+03	2.8849E+03	2.9748E+03	2.9894E+03	2.8951E+03	2.9191E+03
	Avg	2.9284E+03	2.9820E+03	3.0171E+03	3.0391E+03	3.0465E+03	3.0710E+03
	Std	6.1813E+01	9.4234E+01	4.6858E+01	6.5024E+01	7.3058E+01	9.9490E+01
	Rank	1	2	3	4	5	6
F24	Best	2.9071E+03	2.9027E+03	2.9100E+03	2.9039E+03	2.9194E+03	2.9204E+03
	Avg	2.9996E+03	2.9605E+03	2.9345E+03	2.9589E+03	2.9643E+03	2.9796E+03
	Std	1.3307E+02	5.2971E+01	4.7959E+01	6.9244E+01	4.2420E+01	6.4235E+01
	Rank	6	3	1	2	4	5
F25	Best	3.9323E+03	3.4467E+03	3.3116E+03	3.2177E+03	3.7531E+03	3.9780E+03
	Avg	5.0752E+03	4.3811E+03	5.2204E+03	5.5065E+03	5.3894E+03	5.8562E+03
	Std	8.4249E+02	4.2678E+02	7.6924E+02	1.1569E+03	8.0678E+02	1.1979E+03
	Rank	2	1	3	5	4	6
F26	Best	3.2091E+03	3.2122E+03	3.2233E+03	3.2225E+03	3.2484E+03	3.2339E+03
	Avg	3.2535E+03	3.2563E+03	3.2488E+03	3.2629E+03	3.2798E+03	3.3005E+03
	Std	2.4776E+01	3.1958E+01	2.5436E+01	2.9647E+01	3.0476E+01	3.5567E+01
	Rank	2	3	1	4	5	6
F27	Best	3.2944E+03	3.2924E+03	3.2752E+03	3.2582E+03	3.3172E+03	3.2883E+03
	Avg	3.4422E+03	3.3908E+03	3.3592E+03	3.3613E+03	3.4108E+03	3.4209E+03
	Std	1.6191E+02	6.6476E+01	8.3898E+01	6.2604E+01	9.2250E+01	6.9247E+01
	Rank	6	3	1	2	4	5
F28	Best	3.4629E+03	3.6460E+03	3.6488E+03	3.6903E+03	3.9916E+03	3.9825E+03
	Avg	3.9013E+03	3.9208E+03	4.0779E+03	4.0993E+03	4.1891E+03	4.2964E+03
	Std	4.2733E+02	2.5566E+02	3.8359E+02	2.3003E+02	1.5830E+02	2.1895E+02
	Rank	1	2	3	4	5	6
F29	Best	4.1986E+04	1.1403E+04	3.0712E+04	1.7256E+05	1.6194E+05	2.4405E+05
	Avg	5.7659E+05	3.4796E+05	1.3357E+05	4.4427E+05	8.6013E+05	1.9325E+06
	Std	1.0967E+06	8.0472E+05	1.9060E+05	2.3675E+05	8.2743E+05	1.4268E+06
	Rank	4	2	1	3	5	6

**Table A3:** Experiments comparing EECO with different population sizes (50D).

No.	Index	N = 5D	N = 10D	N = 15D	N = 20D	N = 25D	N = 30D
F1	Best	1.0464E+09	3.0916E+09	3.0551E+09	3.4913E+09	2.9233E+09	3.5045E+09
	Avg	5.3003E+09	1.0807E+10	7.7470E+09	8.0086E+09	6.5863E+09	1.1258E+10
	Std	3.3646E+09	1.1003E+10	4.5052E+09	4.0377E+09	3.7751E+09	1.4812E+10
	Rank	1	5	3	4	2	6
F2	Best	9.3818E+03	1.5159E+04	3.7782E+04	3.1256E+04	2.9531E+04	3.6406E+04
	Avg	2.4396E+04	3.1732E+04	6.3519E+04	4.6336E+04	6.1068E+04	7.0945E+04
	Std	1.0754E+04	7.5513E+03	1.0586E+04	1.3716E+04	1.8353E+04	2.5185E+04
	Rank	1	2	5	3	4	6
F3	Best	6.7676E+02	7.6014E+02	9.2967E+02	8.8521E+02	8.1978E+02	9.1823E+02
	Avg	1.0692E+03	1.1221E+03	1.2453E+03	1.4739E+03	1.3279E+03	2.2201E+03
	Std	6.1460E+02	3.8068E+02	3.3289E+02	8.4955E+02	6.0864E+02	1.7009E+03
	Rank	1	2	3	5	4	6
F4	Best	7.9446E+02	7.6121E+02	6.6515E+02	8.5128E+02	7.8145E+02	7.8113E+02
	Avg	8.8007E+02	8.5906E+02	7.2696E+02	9.0908E+02	9.1426E+02	8.8905E+02
	Std	4.2654E+01	3.8750E+01	4.0949E+01	3.5782E+01	5.6573E+01	5.7904E+01
	Rank	3	2	1	5	6	4
F5	Best	6.1612E+02	6.1310E+02	6.2326E+02	6.2206E+02	6.2159E+02	6.2574E+02
	Avg	6.2510E+02	6.2827E+02	6.3251E+02	6.3263E+02	6.3366E+02	6.3677E+02
	Std	4.1335E+00	1.2796E+01	6.3796E+00	9.7574E+00	8.5906E+00	1.0287E+01
	Rank	1	2	3	4	5	6
F6	Best	1.0281E+03	1.1373E+03	1.1504E+03	1.0759E+03	1.0770E+03	9.3922E+02
	Avg	1.2091E+03	1.2487E+03	1.2743E+03	1.2334E+03	1.2226E+03	1.2671E+03
	Std	1.2049E+02	9.7427E+01	7.8379E+01	9.3537E+01	7.2183E+01	1.6243E+02
	Rank	1	4	6	3	2	5
F7	Best	1.0680E+03	1.0602E+03	9.2196E+02	1.1466E+03	1.1100E+03	9.7816E+02
	Avg	1.1722E+03	1.1593E+03	1.0525E+03	1.2239E+03	1.2077E+03	1.2231E+03
	Std	5.0224E+01	5.8248E+01	7.7442E+01	6.8215E+01	4.8648E+01	8.7720E+01
	Rank	3	2	1	6	4	5
F8	Best	3.4203E+03	3.3504E+03	4.3891E+03	3.2046E+03	2.9019E+03	2.2207E+03
	Avg	6.0036E+03	5.3840E+03	1.1629E+04	6.3592E+03	6.0796E+03	6.4834E+03
	Std	3.4545E+03	2.3850E+03	3.8736E+03	4.3122E+03	3.0466E+03	2.8936E+03
	Rank	2	1	6	4	3	5
F9	Best	9.2837E+03	9.3511E+03	9.0986E+03	1.3835E+04	1.1737E+04	8.2585E+03
	Avg	1.3580E+04	1.2659E+04	1.2253E+04	1.4623E+04	1.3777E+04	1.3716E+04
	Std	1.5399E+03	1.3701E+03	1.7768E+03	5.3864E+02	1.1344E+03	2.0576E+03
	Rank	3	2	1	6	5	4
F10	Best	1.3938E+03	1.3416E+03	1.5074E+03	1.4966E+03	1.3672E+03	1.4306E+03
	Avg	1.4900E+03	1.7548E+03	1.6914E+03	1.7216E+03	1.6999E+03	1.7913E+03
	Std	6.1770E+01	5.3185E+02	1.8264E+02	3.5154E+02	1.8644E+02	1.9036E+02
	Rank	1	5	2	4	3	6
F11	Best	9.2151E+06	2.0131E+07	2.6027E+07	1.2509E+07	7.2572E+07	9.8213E+07
	Avg	4.5971E+07	1.3835E+08	1.2548E+08	1.7365E+08	2.2453E+08	8.4313E+08
	Std	2.4595E+07	1.7825E+08	1.1190E+08	1.2774E+08	1.0895E+08	1.9636E+09
	Rank	1	3	2	4	5	6
F12	Best	5.3806E+04	1.6819E+04	2.6321E+04	2.7742E+05	1.1725E+05	7.0723E+04
	Avg	2.1842E+05	5.2744E+04	1.7059E+05	5.6583E+06	5.5632E+06	1.4207E+07
	Std	1.5405E+05	2.2185E+04	4.2624E+05	6.5812E+06	6.0665E+06	1.9591E+07
	Rank	3	1	2	5	4	6
F13	Best	1.6787E+03	1.6482E+03	1.6296E+03	1.9081E+03	4.8425E+03	4.0291E+03
	Avg	2.3773E+03	1.0836E+04	7.6438E+03	7.3343E+03	3.9322E+04	6.1703E+04
	Std	1.5488E+03	3.5310E+04	1.2179E+04	5.3973E+03	3.3461E+04	6.0806E+04
	Rank	1	4	3	2	5	6
F14	Best	4.6681E+03	3.0440E+03	2.8158E+03	1.7606E+04	1.4419E+04	8.3681E+03
	Avg	2.7817E+04	6.8582E+03	1.1374E+04	7.0331E+05	6.2676E+05	6.0336E+05
	Std	2.2540E+04	3.4194E+03	7.5199E+03	7.9233E+05	9.6788E+05	1.5888E+06
	Rank	3	1	2	6	5	4
F15	Best	3.3297E+03	2.3292E+03	2.1006E+03	3.1098E+03	3.3113E+03	4.2440E+03
	Avg	4.0126E+03	3.2417E+03	2.5834E+03	4.3022E+03	4.2646E+03	4.6942E+03
	Std	4.7291E+02	6.0733E+02	2.6591E+02	4.7459E+02	6.2150E+02	3.2460E+02
	Rank	3	2	1	5	4	6
F16	Best	2.8939E+03	2.5164E+03	2.1418E+03	2.7366E+03	2.9775E+03	3.1205E+03
	Avg	3.4015E+03	3.2039E+03	2.5893E+03	3.6512E+03	3.6385E+03	3.8370E+03
	Std	2.7264E+02	3.7580E+02	2.3182E+02	3.8490E+02	2.8816E+02	3.4040E+02
	Rank	3	2	1	5	4	6
F17	Best	9.8506E+03	3.3850E+03	1.2409E+04	4.6311E+04	6.4508E+04	7.8028E+04
	Avg	3.2867E+04	1.2764E+06	2.0844E+05	1.2440E+05	2.5216E+05	3.7391E+05
	Std	1.9536E+04	4.3526E+06	3.7748E+05	5.0930E+04	1.5727E+05	1.8865E+05
	Rank	1	6	3	2	4	5
F18	Best	1.1956E+04	2.1591E+03	5.8497E+03	3.0800E+04	3.0345E+04	2.6560E+04
	Avg	3.5456E+04	1.9431E+04	1.6560E+04	2.2830E+05	3.9427E+05	4.4979E+05
	Std	3.9304E+04	4.4321E+04	8.2289E+03	2.3759E+05	5.9353E+05	7.5544E+05
	Rank	3	2	1	4	5	6
F19	Best	3.3973E+03	2.6651E+03	2.1819E+03	3.3636E+03	2.9016E+03	3.0525E+03
	Avg	3.6828E+03	3.3172E+03	2.5859E+03	3.7605E+03	3.5540E+03	3.6115E+03
	Std	1.7216E+02	3.0118E+02	3.0101E+02	2.0677E+02	4.0841E+02	3.7600E+02
	Rank	5	2	1	6	3	4
F20	Best	2.4986E+03	2.5207E+03	2.4348E+03	2.4586E+03	2.5492E+03	2.5202E+03
	Avg	2.6460E+03	2.5923E+03	2.5007E+03	2.6611E+03	2.6698E+03	2.6835E+03
	Std	4.9408E+01	3.8341E+01	3.8190E+01	6.5735E+01	5.1278E+01	5.8277E+01
	Rank	3	2	1	4	5	6
F21	Best	3.6946E+03	1.2033E+04	9.1883E+03	1.4486E+04	1.3422E+04	1.2342E+04
	Avg	1.4801E+04	1.4784E+04	1.2171E+04	1.5646E+04	1.5295E+04	1.5147E+04
	Std	3.1531E+03	1.3040E+03	2.1954E+03	8.0426E+02	9.4400E+02	1.3927E+03
	Rank	3	2	1	6	5	4
F22	Best	3.0688E+03	3.0165E+03	2.9311E+03	3.0711E+03	3.0468E+03	3.0746E+03
	Avg	3.1764E+03	3.1404E+03	3.0365E+03	3.1847E+03	3.2301E+03	3.2644E+03
	Std	9.3939E+01	9.6981E+01	6.4226E+01	5.6424E+01	7.9354E+01	8.8665E+01
	Rank	3	2	1	4	5	6
F23	Best	3.1152E+03	3.1493E+03	3.0572E+03	3.2403E+03	3.2102E+03	3.3183E+03
	Avg	3.2956E+03	3.2853E+03	3.3328E+03	3.3968E+03	3.3820E+03	3.5209E+03
	Std	1.4672E+02	1.0366E+02	3.0120E+02	1.7779E+02	1.1968E+02	2.3534E+02
	Rank	2	1	3	5	4	6
F24	Best	3.1664E+03	3.1706E+03	3.1268E+03	3.2322E+03	3.2895E+03	3.3186E+03
	Avg	3.7473E+03	3.4845E+03	3.4524E+03	3.6424E+03	3.6854E+03	3.5727E+03
	Std	9.9627E+02	3.3974E+02	1.9600E+02	8.0083E+02	9.6101E+02	1.9396E+02
	Rank	6	2	1	4	5	3
F25	Best	5.0901E+03	4.8387E+03	4.8268E+03	4.5970E+03	6.7452E+03	6.5360E+03
	Avg	7.6361E+03	7.6369E+03	7.6120E+03	7.6965E+03	8.5982E+03	8.6682E+03
	Std	2.0392E+03	1.7365E+03	1.8050E+03	1.4762E+03	1.7689E+03	1.1779E+03
	Rank	2	3	1	4	5	6
F26	Best	3.5313E+03	3.3750E+03	3.5478E+03	3.5080E+03	3.5229E+03	3.6488E+03
	Avg	3.7563E+03	3.7340E+03	3.8769E+03	3.6937E+03	3.8525E+03	3.9255E+03
	Std	2.2633E+02	2.5745E+02	2.4764E+02	1.4404E+02	2.3954E+02	2.1873E+02
	Rank	3	2	5	1	4	6
F27	Best	3.4811E+03	3.6121E+03	3.7378E+03	3.6171E+03	3.5609E+03	3.7847E+03
	Avg	3.9157E+03	4.0797E+03	4.3366E+03	4.2002E+03	4.2898E+03	4.4358E+03
	Std	4.4185E+02	5.7788E+02	9.4325E+02	7.3072E+02	5.8283E+02	4.6252E+02
	Rank	1	2	5	3	4	6
F28	Best	4.3194E+03	4.0313E+03	3.7405E+03	4.5073E+03	4.4327E+03	4.8778E+03
	Avg	4.9161E+03	4.4880E+03	4.4059E+03	5.0906E+03	5.0526E+03	5.3316E+03
	Std	2.7640E+02	3.8673E+02	5.4027E+02	3.2549E+02	3.8052E+02	4.0370E+02
	Rank	3	2	1	5	4	6
F29	Best	1.2056E+07	4.6847E+06	3.7770E+06	1.9966E+07	2.0389E+07	2.8353E+07
	Avg	1.6003E+07	1.3920E+07	1.2476E+07	2.8806E+07	3.4954E+07	5.7312E+07
	Std	4.7722E+06	8.5395E+06	5.1979E+06	6.9741E+06	1.0557E+07	1.6429E+07
	Rank	3	2	1	4	5	6

**Table A4:** Experiments comparing EECO with different population sizes (100D).

No.	Index	N = 5D	N = 10D	N = 15D	N = 20D	N = 25D	N = 30D
F1	Best	2.7424E+10	3.0968E+10	2.7495E+10	2.3895E+10	2.4130E+10	3.1645E+10
	Avg	4.4130E+10	5.8942E+10	4.1016E+10	6.0823E+10	4.2234E+10	4.7684E+10
	Std	1.4819E+10	2.9826E+10	9.9627E+09	3.5880E+10	1.3624E+10	1.1848E+10
	Rank	3	5	1	6	2	4
F2	Best	6.7138E+04	8.6366E+04	1.3358E+05	8.0065E+04	1.2236E+05	1.0682E+05
	Avg	1.1729E+05	1.1374E+05	1.6201E+05	1.7541E+05	2.2434E+05	1.9414E+05
	Std	2.3725E+04	1.3889E+04	1.5676E+04	4.2043E+04	5.0457E+04	6.0866E+04
	Rank	2	1	3	4	6	5
F3	Best	2.8108E+03	3.0741E+03	3.0844E+03	3.4000E+03	2.9105E+03	2.9078E+03
	Avg	5.4631E+03	5.5275E+03	4.6179E+03	5.3604E+03	5.0295E+03	5.3793E+03
	Std	3.5381E+03	4.9257E+03	1.3133E+03	2.0167E+03	2.4151E+03	1.4562E+03
	Rank	5	6	1	3	2	4
F4	Best	1.3154E+03	1.1481E+03	1.0068E+03	1.2614E+03	1.1603E+03	9.4633E+02
	Avg	1.4464E+03	1.4168E+03	1.1181E+03	1.5260E+03	1.4750E+03	1.4546E+03
	Std	6.7499E+01	1.3672E+02	9.0432E+01	1.0989E+02	1.2540E+02	2.0673E+02
	Rank	3	2	1	6	5	4
F5	Best	6.2727E+02	6.3656E+02	6.3700E+02	6.3902E+02	6.4173E+02	6.3622E+02
	Avg	6.4468E+02	6.4664E+02	6.4812E+02	6.5393E+02	6.5107E+02	6.5735E+02
	Std	1.2014E+01	6.6575E+00	9.1135E+00	1.0088E+01	5.8974E+00	1.0451E+01
	Rank	1	2	3	5	4	6
F6	Best	1.8813E+03	1.8761E+03	2.1588E+03	1.8276E+03	1.7677E+03	1.8381E+03
	Avg	2.0754E+03	2.3754E+03	2.4122E+03	2.2432E+03	2.1310E+03	2.1230E+03
	Std	1.3942E+02	4.5267E+02	1.6876E+02	4.1488E+02	1.9126E+02	2.0851E+02
	Rank	1	5	6	4	3	2
F7	Best	1.5475E+03	1.6299E+03	1.4184E+03	1.7225E+03	1.5943E+03	1.3741E+03
	Avg	1.7774E+03	1.7349E+03	1.6069E+03	1.8639E+03	1.8652E+03	1.8136E+03
	Std	1.1006E+02	7.6319E+01	1.4096E+02	1.2829E+02	1.1927E+02	1.7620E+02
	Rank	3	2	1	5	6	4
F8	Best	1.1040E+04	1.3008E+04	2.0610E+04	1.4648E+04	1.7776E+04	1.5389E+04
	Avg	1.8543E+04	2.1323E+04	3.2922E+04	2.0647E+04	2.3444E+04	2.7835E+04
	Std	8.9386E+03	5.9535E+03	9.9816E+03	3.0739E+03	4.3438E+03	1.0002E+04
	Rank	1	3	6	2	4	5
F9	Best	2.7277E+04	2.4461E+04	1.9896E+04	2.6308E+04	2.0836E+04	2.3208E+04
	Avg	2.9595E+04	2.8976E+04	2.6372E+04	3.0256E+04	2.9536E+04	2.9939E+04
	Std	1.0632E+03	1.5836E+03	3.2734E+03	1.2940E+03	2.8161E+03	2.2931E+03
	Rank	4	2	1	6	3	5
F10	Best	3.8263E+03	4.7783E+03	9.0616E+03	7.7991E+03	7.3580E+03	9.9601E+03
	Avg	5.5642E+03	7.2523E+03	1.3501E+04	1.0857E+04	1.2398E+04	1.7791E+04
	Std	1.2877E+03	2.3123E+03	3.6198E+03	2.3240E+03	3.1906E+03	5.1209E+03
	Rank	1	2	5	3	4	6
F11	Best	3.5322E+08	3.0289E+08	9.9015E+08	1.3562E+09	1.3291E+09	1.8839E+09
	Avg	1.7146E+09	2.0433E+09	2.0248E+09	6.0440E+09	2.8868E+09	8.5157E+09
	Std	7.5608E+08	1.8903E+09	7.5303E+08	1.0686E+10	1.2851E+09	1.0470E+10
	Rank	1	3	2	5	4	6
F12	Best	1.7646E+05	4.4467E+04	4.7572E+05	3.3105E+05	2.2444E+06	6.0832E+06
	Avg	6.2341E+07	8.9911E+05	5.5354E+06	1.2441E+08	3.7394E+07	2.5383E+08
	Std	2.2817E+08	2.8723E+06	7.2221E+06	2.0676E+08	3.8052E+07	5.9431E+08
	Rank	4	1	2	5	3	6
F13	Best	6.4071E+03	1.9937E+03	2.0940E+03	7.0230E+04	8.2505E+04	1.6272E+05
	Avg	2.9614E+05	9.0557E+03	3.5874E+04	3.1959E+05	5.4612E+05	7.6278E+05
	Std	9.1873E+05	1.8196E+04	1.0303E+05	3.4296E+05	4.0678E+05	5.5985E+05
	Rank	3	1	2	4	5	6
F14	Best	3.0459E+04	2.3567E+04	1.6075E+04	5.4906E+04	1.5094E+05	4.4770E+04
	Avg	9.5486E+06	4.3846E+04	1.5619E+05	8.7603E+06	8.2069E+06	7.5711E+07
	Std	3.4679E+07	1.3152E+04	2.5901E+05	7.4531E+06	1.3201E+07	2.2572E+08
	Rank	5	1	2	4	3	6
F15	Best	7.4498E+03	5.8129E+03	4.0486E+03	8.4723E+03	7.1670E+03	7.2910E+03
	Avg	8.7536E+03	7.3314E+03	4.9125E+03	9.4798E+03	9.1767E+03	9.1983E+03
	Std	6.2581E+02	9.5695E+02	1.1495E+03	6.4022E+02	9.4374E+02	1.0226E+03
	Rank	3	2	1	6	4	5
F16	Best	5.8807E+03	4.5149E+03	2.9141E+03	6.3810E+03	5.2269E+03	5.2125E+03
	Avg	6.6045E+03	5.8587E+03	3.7514E+03	6.8512E+03	6.9234E+03	6.9761E+03
	Std	4.2444E+02	6.0854E+02	4.2169E+02	3.7580E+02	5.9571E+02	1.0512E+03
	Rank	3	2	1	4	5	6
F17	Best	7.0872E+04	2.3135E+04	4.5388E+04	6.3515E+04	2.5016E+05	3.8803E+05
	Avg	1.2727E+05	2.0645E+05	3.3767E+05	1.6586E+06	5.1464E+05	9.9208E+05
	Std	5.6413E+04	5.0653E+05	7.6165E+05	4.3100E+06	2.0929E+05	6.1496E+05
	Rank	1	2	3	6	4	5
F18	Best	4.6670E+05	4.1251E+04	4.9532E+04	3.5587E+06	6.5170E+05	3.0289E+06
	Avg	2.2325E+06	3.6478E+05	4.7194E+05	8.5298E+07	1.5612E+07	3.2480E+07
	Std	2.5526E+06	3.8973E+05	4.9518E+05	2.8740E+08	1.7119E+07	2.1318E+07
	Rank	3	1	2	6	4	5
F19	Best	4.7946E+03	4.9490E+03	3.5711E+03	6.1426E+03	5.5446E+03	5.1262E+03
	Avg	6.1535E+03	6.3453E+03	5.6038E+03	6.8657E+03	6.5441E+03	6.5858E+03
	Std	7.8239E+02	6.1734E+02	8.0650E+02	4.1745E+02	5.8776E+02	8.3253E+02
	Rank	2	3	1	6	4	5
F20	Best	3.0644E+03	2.9418E+03	2.9295E+03	3.2837E+03	3.1794E+03	3.1473E+03
	Avg	3.2476E+03	3.2312E+03	3.0014E+03	3.3643E+03	3.3496E+03	3.3877E+03
	Std	8.7357E+01	1.0565E+02	6.1309E+01	4.3471E+01	8.5855E+01	1.1763E+02
	Rank	3	2	1	5	4	6
F21	Best	2.9361E+04	2.8697E+04	2.4900E+04	3.2012E+04	2.9016E+04	2.5937E+04
	Avg	3.2480E+04	3.1661E+04	2.9452E+04	3.3402E+04	3.3101E+04	3.1812E+04
	Std	1.2865E+03	1.6274E+03	2.7142E+03	9.0638E+02	1.3543E+03	2.2313E+03
	Rank	4	2	1	6	5	3
F22	Best	3.4921E+03	3.5301E+03	3.4506E+03	3.8355E+03	3.8621E+03	3.7367E+03
	Avg	3.9566E+03	4.0000E+03	3.8616E+03	4.0799E+03	4.1848E+03	4.2368E+03
	Std	1.7212E+02	3.3333E+02	3.9590E+02	1.6790E+02	1.5746E+02	3.3100E+02
	Rank	2	3	1	4	5	6
F23	Best	4.2560E+03	4.2306E+03	3.9076E+03	4.3594E+03	4.3743E+03	4.4600E+03
	Avg	5.1306E+03	4.7821E+03	4.5290E+03	4.7587E+03	4.9304E+03	5.3968E+03
	Std	7.6657E+02	5.9035E+02	3.5939E+02	3.0000E+02	3.6679E+02	7.5880E+02
	Rank	5	3	1	2	4	6
F24	Best	4.1692E+03	5.2789E+03	5.3113E+03	4.9554E+03	4.8405E+03	5.1824E+03
	Avg	5.6078E+03	5.8588E+03	6.0214E+03	6.7563E+03	5.7237E+03	6.1900E+03
	Std	1.8595E+03	5.0606E+02	4.7341E+02	2.7107E+03	5.8016E+02	7.0538E+02
	Rank	1	3	4	6	2	5
F25	Best	1.3059E+04	1.3291E+04	1.0277E+04	9.2088E+03	1.6261E+04	1.5577E+04
	Avg	2.0002E+04	1.9832E+04	1.6042E+04	1.9631E+04	2.2143E+04	2.1181E+04
	Std	5.2108E+03	5.7504E+03	2.2874E+03	6.3450E+03	6.7575E+03	5.0131E+03
	Rank	4	3	1	2	6	5
F26	Best	3.7274E+03	3.7020E+03	3.9083E+03	4.1078E+03	4.2458E+03	3.9833E+03
	Avg	4.2223E+03	4.1909E+03	4.1651E+03	4.5435E+03	4.7845E+03	4.6682E+03
	Std	4.0082E+02	3.6374E+02	2.6607E+02	4.7865E+02	4.7883E+02	3.4158E+02
	Rank	3	2	1	4	6	5
F27	Best	4.5786E+03	5.9173E+03	5.9007E+03	6.3944E+03	5.5418E+03	7.2794E+03
	Avg	8.1156E+03	8.7979E+03	7.4144E+03	8.7920E+03	9.2086E+03	1.0376E+04
	Std	2.5880E+03	3.0476E+03	8.6071E+02	1.6567E+03	4.1532E+03	3.0861E+03
	Rank	2	4	1	3	5	6
F28	Best	7.9974E+03	7.0598E+03	6.6154E+03	8.9851E+03	8.9387E+03	9.8518E+03
	Avg	9.0509E+03	8.6145E+03	7.2790E+03	1.0078E+04	1.0372E+04	1.1322E+04
	Std	7.6525E+02	8.3251E+02	4.3452E+02	6.4818E+02	6.9294E+02	7.9124E+02
	Rank	3	2	1	4	5	6
F29	Best	1.1347E+07	3.8130E+06	5.3980E+06	6.7692E+07	1.3742E+08	1.2327E+08
	Avg	4.6708E+07	6.3340E+07	2.4786E+07	3.4457E+08	2.5812E+08	2.6304E+08
	Std	2.7969E+07	1.7160E+08	1.8235E+07	5.6307E+08	1.3436E+08	1.1693E+08
	Rank	2	3	1	6	4	5

**Table A5:** Experiments comparing EECO with different parameter as (10D).

No.	Index	ECO	a = 0.5	a = 0.6	a = 0.7	a = 0.8	a = 0.9
F1	Best	6.3568E+06	1.0000E+02	1.0000E+02	1.0000E+02	1.0000E+02	1.0000E+02
	Avg	1.5632E+08	1.0004E+02	1.0000E+02	1.0015E+02	1.0000E+02	1.0000E+02
	Std	3.1359E+08	1.3750E-01	2.3854E-07	5.8681E-01	2.1836E-07	2.0234E-07
	Rank	6	4	1	5	3	2
F2	Best	7.2490E+02	3.0000E+02	3.0000E+02	3.0000E+02	3.0000E+02	3.0000E+02
	Avg	3.1268E+03	3.0000E+02	3.0000E+02	3.0000E+02	3.0000E+02	3.0000E+02
	Std	2.1573E+03	1.8695E-11	1.0756E-11	2.4836E-11	7.4740E-12	2.7136E-11
	Rank	6	3	2	4	1	5
F3	Best	4.0663E+02	4.0000E+02	4.0000E+02	4.0000E+02	4.0000E+02	4.0000E+02
	Avg	4.2652E+02	4.0000E+02	4.0000E+02	4.0000E+02	4.0000E+02	4.0000E+02
	Std	2.3682E+01	1.4492E-13	8.9440E-13	4.5829E-13	1.2855E-12	2.4948E-12
	Rank	6	1	4	2	3	5
F4	Best	5.1504E+02	5.1492E+02	5.0895E+02	5.0696E+02	5.0696E+02	5.0696E+02
	Avg	5.4079E+02	5.3098E+02	5.2972E+02	5.2978E+02	5.2912E+02	5.2680E+02
	Std	1.4548E+01	1.9987E+01	1.7686E+01	1.8452E+01	1.8853E+01	1.8017E+01
	Rank	6	5	3	4	2	1
F5	Best	6.0731E+02	6.0995E+02	6.0694E+02	6.0643E+02	6.0516E+02	6.0578E+02
	Avg	6.2141E+02	6.1782E+02	6.1501E+02	6.1395E+02	6.1352E+02	6.1254E+02
	Std	9.6906E+00	5.9686E+00	5.8454E+00	4.7690E+00	5.9790E+00	5.8255E+00
	Rank	6	5	4	3	2	1
F6	Best	7.2950E+02	7.1403E+02	7.1403E+02	7.1789E+02	7.1789E+02	7.1789E+02
	Avg	7.6287E+02	7.5129E+02	7.4851E+02	7.4684E+02	7.4524E+02	7.4411E+02
	Std	1.8573E+01	1.7442E+01	1.6104E+01	1.4205E+01	1.3200E+01	1.3109E+01
	Rank	6	5	4	3	2	1
F7	Best	8.1459E+02	8.0995E+02	8.0995E+02	8.0995E+02	8.0995E+02	8.0995E+02
	Avg	8.3687E+02	8.2229E+02	8.2295E+02	8.2235E+02	8.2235E+02	8.2235E+02
	Std	9.3431E+00	8.6803E+00	9.0993E+00	9.1874E+00	9.1874E+00	9.1874E+00
	Rank	6	1	5	2	2	4
F8	Best	9.2354E+02	9.1561E+02	9.1540E+02	9.0486E+02	9.1734E+02	9.0961E+02
	Avg	1.0787E+03	1.1089E+03	1.0707E+03	1.0544E+03	1.0789E+03	1.0680E+03
	Std	1.2133E+02	1.6866E+02	1.1160E+02	1.1246E+02	1.4738E+02	1.4369E+02
	Rank	4	6	3	1	5	2
F9	Best	1.6251E+03	1.5237E+03	1.2540E+03	1.2540E+03	1.2540E+03	1.2540E+03
	Avg	2.0675E+03	1.9178E+03	1.7884E+03	1.7925E+03	1.7952E+03	1.8071E+03
	Std	3.3508E+02	2.1044E+02	2.8604E+02	2.9603E+02	2.6213E+02	2.6405E+02
	Rank	6	5	1	2	3	4
F10	Best	1.1602E+03	1.1216E+03	1.1216E+03	1.1209E+03	1.1209E+03	1.1216E+03
	Avg	1.2608E+03	1.1836E+03	1.1737E+03	1.1757E+03	1.1761E+03	1.1746E+03
	Std	9.8558E+01	4.9742E+01	4.5508E+01	4.6263E+01	4.5928E+01	4.0861E+01
	Rank	6	5	1	3	4	2
F11	Best	9.6759E+04	1.3460E+03	1.2112E+03	1.3585E+03	1.3186E+03	1.3186E+03
	Avg	4.9010E+06	1.7013E+03	1.5941E+03	1.6350E+03	2.1391E+03	2.0787E+03
	Std	6.3820E+06	1.8958E+02	2.2521E+02	1.0398E+02	1.5224E+03	6.7431E+02
	Rank	6	3	1	2	5	4
F12	Best	1.9964E+03	1.3853E+03	1.3248E+03	1.5295E+03	1.4370E+03	1.5049E+03
	Avg	1.5972E+04	1.8783E+03	1.7995E+03	1.8864E+03	1.8507E+03	1.9453E+03
	Std	1.8296E+04	3.0653E+02	2.9808E+02	3.0359E+02	3.1223E+02	2.3976E+02
	Rank	6	3	1	4	2	5
F13	Best	1.4708E+03	1.4339E+03	1.4315E+03	1.4348E+03	1.4341E+03	1.4352E+03
	Avg	1.5422E+03	1.4802E+03	1.5067E+03	1.4912E+03	1.4893E+03	1.4892E+03
	Std	8.5245E+01	3.2990E+01	8.1397E+01	5.2430E+01	3.9156E+01	3.4019E+01
	Rank	6	1	5	4	3	2
F14	Best	1.6616E+03	1.5022E+03	1.5015E+03	1.5035E+03	1.5035E+03	1.5052E+03
	Avg	2.5911E+03	1.5958E+03	1.6046E+03	1.5968E+03	1.6003E+03	1.6116E+03
	Std	1.0180E+03	9.5218E+01	9.8870E+01	9.0882E+01	6.0997E+01	6.6745E+01
	Rank	6	1	4	2	3	5
F15	Best	1.6103E+03	1.6012E+03	1.6012E+03	1.6014E+03	1.6014E+03	1.6014E+03
	Avg	1.7834E+03	1.6853E+03	1.6906E+03	1.6918E+03	1.6999E+03	1.6927E+03
	Std	1.0502E+02	8.2294E+01	8.5838E+01	7.6911E+01	8.5837E+01	7.7187E+01
	Rank	6	1	2	3	5	4
F16	Best	1.7553E+03	1.7301E+03	1.7286E+03	1.7286E+03	1.7308E+03	1.7286E+03
	Avg	1.7989E+03	1.7643E+03	1.7635E+03	1.7612E+03	1.7631E+03	1.7633E+03
	Std	4.2116E+01	4.1763E+01	3.0504E+01	2.9553E+01	3.0365E+01	3.0083E+01
	Rank	6	5	4	1	2	3
F17	Best	2.8667E+03	1.8346E+03	1.8263E+03	1.8231E+03	1.8265E+03	1.8265E+03
	Avg	2.0116E+04	1.8813E+03	1.8956E+03	1.8755E+03	1.8945E+03	1.8899E+03
	Std	1.5720E+04	3.8931E+01	5.4760E+01	5.3272E+01	7.5361E+01	8.2343E+01
	Rank	6	2	5	1	4	3
F18	Best	1.9546E+03	1.9086E+03	1.9173E+03	1.9179E+03	1.9150E+03	1.9132E+03
	Avg	3.0823E+03	1.9760E+03	1.9733E+03	1.9860E+03	1.9786E+03	1.9762E+03
	Std	1.8128E+03	4.7324E+01	4.0777E+01	4.5941E+01	4.0846E+01	5.2675E+01
	Rank	6	2	1	5	4	3
F19	Best	2.0621E+03	2.0375E+03	2.0379E+03	2.0393E+03	2.0390E+03	2.0395E+03
	Avg	2.1270E+03	2.1068E+03	2.0896E+03	2.0858E+03	2.0841E+03	2.0808E+03
	Std	4.6113E+01	4.5178E+01	3.3625E+01	3.1574E+01	3.6869E+01	3.2887E+01
	Rank	6	5	4	3	2	1
F20	Best	2.2086E+03	2.2000E+03	2.2000E+03	2.2000E+03	2.2024E+03	2.2000E+03
	Avg	2.2536E+03	2.2602E+03	2.2618E+03	2.2597E+03	2.2599E+03	2.2598E+03
	Std	5.2251E+01	6.4014E+01	6.6419E+01	6.3548E+01	6.3311E+01	6.3435E+01
	Rank	1	5	6	2	4	3
F21	Best	2.2577E+03	2.2261E+03	2.2261E+03	2.2116E+03	2.2116E+03	2.2261E+03
	Avg	2.3184E+03	2.2991E+03	2.2948E+03	2.2956E+03	2.2966E+03	2.2928E+03
	Std	2.8828E+01	2.4555E+01	3.2214E+01	3.2918E+01	3.6065E+01	2.7818E+01
	Rank	6	5	2	3	4	1
F22	Best	2.6198E+03	2.6149E+03	2.6121E+03	2.6115E+03	2.6115E+03	2.6115E+03
	Avg	2.6346E+03	2.6264E+03	2.6248E+03	2.6245E+03	2.6239E+03	2.6239E+03
	Std	1.9097E+01	9.6911E+00	9.7082E+00	1.0282E+01	9.8880E+00	9.8901E+00
	Rank	6	5	4	3	1	2
F23	Best	2.5475E+03	2.5000E+03	2.5000E+03	2.5000E+03	2.5000E+03	2.5000E+03
	Avg	2.7375E+03	2.7221E+03	2.7186E+03	2.7204E+03	2.7204E+03	2.7204E+03
	Std	6.7204E+01	9.2320E+01	8.9605E+01	9.1787E+01	9.1787E+01	9.1787E+01
	Rank	6	5	1	4	3	2
F24	Best	2.9133E+03	2.8980E+03	2.8980E+03	2.8977E+03	2.8978E+03	2.8980E+03
	Avg	2.9530E+03	2.9352E+03	2.9358E+03	2.9364E+03	2.9363E+03	2.9363E+03
	Std	2.3641E+01	2.2379E+01	2.2636E+01	2.1658E+01	2.3095E+01	2.3348E+01
	Rank	6	1	2	5	4	3
F25	Best	2.9206E+03	2.6000E+03	2.6000E+03	2.6000E+03	2.6000E+03	2.6000E+03
	Avg	3.1026E+03	3.0497E+03	3.0374E+03	3.0345E+03	3.0110E+03	3.0121E+03
	Std	1.0755E+02	1.8396E+02	1.8157E+02	1.9442E+02	1.8356E+02	1.8422E+02
	Rank	6	5	4	3	1	2
F26	Best	3.0914E+03	3.0889E+03	3.0838E+03	3.0880E+03	3.0884E+03	3.0841E+03
	Avg	3.1013E+03	3.0980E+03	3.0962E+03	3.0949E+03	3.0945E+03	3.0928E+03
	Std	1.1701E+01	1.8074E+01	1.0668E+01	4.7820E+00	4.9097E+00	4.8664E+00
	Rank	6	5	4	3	2	1
F27	Best	3.1801E+03	3.1000E+03	3.1000E+03	3.1000E+03	3.1000E+03	3.1000E+03
	Avg	3.4163E+03	3.2662E+03	3.2643E+03	3.2566E+03	3.2530E+03	3.2588E+03
	Std	1.3588E+02	5.4372E+01	5.3608E+01	5.6318E+01	5.4080E+01	5.0822E+01
	Rank	6	5	4	2	1	3
F28	Best	3.1753E+03	3.1555E+03	3.1477E+03	3.1472E+03	3.1488E+03	3.1488E+03
	Avg	3.2381E+03	3.2308E+03	3.2357E+03	3.2287E+03	3.2309E+03	3.2265E+03
	Std	4.2469E+01	7.0588E+01	7.1072E+01	6.1748E+01	7.1347E+01	6.2751E+01
	Rank	6	3	5	2	4	1
F29	Best	1.9416E+04	3.3158E+03	3.2318E+03	3.2658E+03	3.2322E+03	3.2737E+03
	Avg	8.0401E+05	3.7135E+03	3.4197E+03	3.3965E+03	3.4133E+03	3.3686E+03
	Std	7.4313E+05	1.0255E+03	2.2074E+02	1.4140E+02	1.3867E+02	8.6409E+01
	Rank	6	5	4	2	3	1

**Table A6:** Experiments comparing EECO with different parameter as (30D).

No.	Index	ECO	a = 0.5	a = 0.6	a = 0.7	a = 0.8	a = 0.9
F1	Best	1.4202E+09	1.0000E+02	1.0000E+02	1.0000E+02	1.0000E+02	1.0000E+02
	Avg	5.2751E+09	1.0000E+02	1.0000E+02	1.0023E+02	1.0000E+02	1.0000E+02
	Std	3.8240E+09	4.0702E-07	5.4950E-06	7.4310E-01	6.4302E-07	1.8528E-05
	Rank	6	2	3	5	1	4
F2	Best	3.8187E+04	3.0000E+02	3.0000E+02	3.0000E+02	3.0000E+02	3.0000E+02
	Avg	5.3594E+04	3.0000E+02	3.0000E+02	3.0000E+02	3.0000E+02	3.0000E+02
	Std	1.1290E+04	8.6284E-07	5.3625E-07	9.9798E-07	1.0239E-06	1.3597E-06
	Rank	6	4	1	5	3	2
F3	Best	9.1908E+02	4.0000E+02	4.0000E+02	4.0000E+02	4.0000E+02	4.0000E+02
	Avg	1.2338E+03	4.0239E+02	4.0319E+02	4.0279E+02	4.0279E+02	4.0319E+02
	Std	2.7164E+02	2.0587E+00	1.6809E+00	1.9257E+00	1.9257E+00	1.6809E+00
	Rank	6	1	5	3	2	4
F4	Best	6.7284E+02	5.6766E+02	5.7562E+02	5.7462E+02	5.7263E+02	5.7562E+02
	Avg	7.4774E+02	6.6954E+02	6.6825E+02	6.6994E+02	6.6775E+02	6.6894E+02
	Std	4.4218E+01	6.2440E+01	6.1067E+01	6.0381E+01	6.1221E+01	6.0235E+01
	Rank	6	4	2	5	1	3
F5	Best	6.3203E+02	6.3027E+02	6.3028E+02	6.2677E+02	6.1978E+02	6.1952E+02
	Avg	6.5026E+02	6.4077E+02	6.4029E+02	6.3998E+02	6.3838E+02	6.3744E+02
	Std	1.1109E+01	6.9300E+00	6.7559E+00	7.1047E+00	8.0791E+00	8.3091E+00
	Rank	6	5	4	3	2	1
F6	Best	1.0318E+03	8.9463E+02	8.9993E+02	9.0147E+02	8.9855E+02	8.4193E+02
	Avg	1.1395E+03	1.0182E+03	9.9774E+02	9.9754E+02	9.9667E+02	9.8756E+02
	Std	6.9933E+01	1.2117E+02	1.2158E+02	1.2146E+02	1.2113E+02	1.2815E+02
	Rank	6	5	4	3	2	1
F7	Best	9.4569E+02	8.9950E+02	8.9751E+02	8.9751E+02	8.9054E+02	8.9154E+02
	Avg	1.0338E+03	9.4994E+02	9.4715E+02	9.4745E+02	9.4556E+02	9.4616E+02
	Std	4.1974E+01	3.3172E+01	3.0989E+01	3.1090E+01	3.2468E+01	3.2364E+01
	Rank	6	5	3	4	1	2
F8	Best	4.0687E+03	1.8912E+03	1.9147E+03	1.8923E+03	1.8841E+03	1.8940E+03
	Avg	7.1373E+03	3.7637E+03	3.7640E+03	3.7625E+03	3.7536E+03	3.7692E+03
	Std	1.7726E+03	1.2705E+03	1.2663E+03	1.2667E+03	1.2724E+03	1.2570E+03
	Rank	6	3	4	2	1	5
F9	Best	7.0669E+03	3.9684E+03	3.9684E+03	3.7607E+03	3.7607E+03	3.6619E+03
	Avg	7.6756E+03	5.1046E+03	5.1429E+03	4.9384E+03	5.0141E+03	4.9202E+03
	Std	4.9056E+02	5.4236E+02	7.2399E+02	7.9556E+02	8.6859E+02	7.3501E+02
	Rank	6	4	5	2	3	1
F10	Best	1.5595E+03	1.1716E+03	1.1677E+03	1.1677E+03	1.1677E+03	1.1677E+03
	Avg	2.7350E+03	1.2293E+03	1.2296E+03	1.2193E+03	1.2307E+03	1.2279E+03
	Std	1.0929E+03	4.2976E+01	5.3487E+01	3.7497E+01	4.9668E+01	4.4332E+01
	Rank	6	3	4	1	5	2
F11	Best	4.4269E+07	1.8633E+03	1.6415E+03	1.6425E+03	2.3723E+03	2.0319E+03
	Avg	2.6305E+08	2.5424E+03	2.5355E+03	2.6446E+03	2.9599E+03	3.8274E+03
	Std	3.3804E+08	4.0659E+02	7.4483E+02	6.2040E+02	6.9366E+02	3.7652E+03
	Rank	6	2	1	3	4	5
F12	Best	1.4244E+05	1.7569E+03	2.0335E+03	2.3438E+03	2.1797E+03	2.5039E+03
	Avg	4.3495E+06	2.8663E+03	2.9544E+03	3.5312E+03	3.6218E+03	3.1982E+03
	Std	5.1856E+06	6.1828E+02	8.4343E+02	8.6897E+02	9.9961E+02	6.1612E+02
	Rank	6	1	2	4	5	3
F13	Best	9.6618E+03	1.5030E+03	1.5357E+03	1.5073E+03	1.4920E+03	1.5603E+03
	Avg	2.0121E+05	1.6108E+03	1.5946E+03	1.6030E+03	1.6636E+03	1.6369E+03
	Std	4.8201E+05	8.3032E+01	5.8757E+01	6.9870E+01	7.5948E+01	5.1682E+01
	Rank	6	3	1	2	5	4
F14	Best	2.5911E+04	1.6496E+03	1.5781E+03	1.6266E+03	1.6336E+03	1.5711E+03
	Avg	1.2174E+06	1.7182E+03	1.8883E+03	1.7263E+03	1.7574E+03	1.8400E+03
	Std	1.9415E+06	5.3115E+01	3.0372E+02	1.1643E+02	1.1380E+02	2.1117E+02
	Rank	6	1	5	2	3	4
F15	Best	2.8693E+03	2.2821E+03	2.1648E+03	2.1815E+03	2.0428E+03	2.0428E+03
	Avg	3.4525E+03	2.7600E+03	2.6854E+03	2.6362E+03	2.6322E+03	2.6366E+03
	Std	3.6857E+02	2.9890E+02	3.7439E+02	2.9228E+02	3.1478E+02	3.1501E+02
	Rank	6	5	4	2	1	3
F16	Best	2.1880E+03	1.8460E+03	1.7884E+03	1.7877E+03	1.7872E+03	1.7833E+03
	Avg	2.3653E+03	2.1876E+03	2.1556E+03	2.1624E+03	2.0865E+03	2.0799E+03
	Std	1.6112E+02	3.6427E+02	3.4803E+02	3.5899E+02	2.4206E+02	2.4010E+02
	Rank	6	5	3	4	2	1
F17	Best	8.3824E+04	1.8749E+03	1.8352E+03	1.9304E+03	1.8740E+03	1.8847E+03
	Avg	8.5969E+05	1.9670E+03	1.9808E+03	1.9890E+03	1.9645E+03	2.8078E+03
	Std	1.1545E+06	7.8074E+01	1.0036E+02	6.0686E+01	7.7551E+01	1.3783E+03
	Rank	6	2	3	4	1	5
F18	Best	1.0096E+04	2.0982E+03	2.0194E+03	2.1319E+03	2.0294E+03	2.1536E+03
	Avg	8.0005E+05	2.2583E+03	2.2231E+03	2.3355E+03	2.2744E+03	3.2714E+03
	Std	1.5036E+06	1.1587E+02	1.4100E+02	2.1651E+02	2.2885E+02	1.7664E+03
	Rank	6	2	1	4	3	5
F19	Best	2.4491E+03	2.3036E+03	2.3046E+03	2.4117E+03	2.3846E+03	2.4027E+03
	Avg	2.6652E+03	2.5553E+03	2.5454E+03	2.5620E+03	2.5587E+03	2.5562E+03
	Std	1.7671E+02	1.4506E+02	1.2500E+02	1.0097E+02	1.0561E+02	9.6848E+01
	Rank	6	2	1	5	4	3
F20	Best	2.4608E+03	2.3552E+03	2.3552E+03	2.3424E+03	2.3459E+03	2.3444E+03
	Avg	2.5290E+03	2.4273E+03	2.4197E+03	2.4149E+03	2.4159E+03	2.4155E+03
	Std	4.7807E+01	5.3818E+01	4.8297E+01	4.9075E+01	4.7729E+01	4.8298E+01
	Rank	6	5	4	1	3	2
F21	Best	2.5992E+03	2.3000E+03	2.3000E+03	2.3000E+03	2.3000E+03	2.3000E+03
	Avg	5.5979E+03	5.1563E+03	5.2025E+03	5.2412E+03	5.1948E+03	5.1970E+03
	Std	2.6890E+03	2.5159E+03	2.5502E+03	2.5864E+03	2.5404E+03	2.5433E+03
	Rank	6	1	4	5	2	3
F22	Best	2.8342E+03	2.7769E+03	2.7669E+03	2.7771E+03	2.7771E+03	2.7771E+03
	Avg	2.9360E+03	2.8413E+03	2.8270E+03	2.8285E+03	2.8223E+03	2.8221E+03
	Std	6.8458E+01	4.2575E+01	4.2612E+01	3.8588E+01	4.1651E+01	4.1427E+01
	Rank	6	5	3	4	2	1
F23	Best	2.9924E+03	2.9348E+03	2.9260E+03	2.9147E+03	2.9123E+03	2.9109E+03
	Avg	3.0454E+03	3.0422E+03	3.0241E+03	3.0179E+03	3.0077E+03	3.0060E+03
	Std	3.9001E+01	6.2653E+01	6.4212E+01	6.5875E+01	5.9665E+01	6.0296E+01
	Rank	6	5	4	3	2	1
F24	Best	3.0607E+03	2.8752E+03	2.8753E+03	2.8752E+03	2.8752E+03	2.8751E+03
	Avg	3.2809E+03	2.8780E+03	2.8778E+03	2.8785E+03	2.8778E+03	2.8867E+03
	Std	1.3704E+02	3.8366E+00	2.8270E+00	3.1429E+00	2.9136E+00	1.9711E+01
	Rank	6	3	1	4	2	5
F25	Best	4.7183E+03	2.8000E+03	2.8000E+03	2.8000E+03	2.8000E+03	2.8000E+03
	Avg	6.4322E+03	5.0041E+03	5.0160E+03	5.0279E+03	5.0357E+03	5.0439E+03
	Std	8.4653E+02	8.2847E+02	8.7033E+02	8.6496E+02	8.7242E+02	8.7179E+02
	Rank	6	1	2	3	4	5
F26	Best	3.2573E+03	3.2000E+03	3.2000E+03	3.1685E+03	3.1644E+03	3.1652E+03
	Avg	3.3100E+03	3.2000E+03	3.2000E+03	3.1969E+03	3.1932E+03	3.1935E+03
	Std	3.0583E+01	8.3370E-05	1.2162E-04	9.9486E+00	1.4287E+01	1.3797E+01
	Rank	6	5	4	3	1	2
F27	Best	3.7060E+03	3.1000E+03	3.1000E+03	3.1000E+03	3.1000E+03	3.1000E+03
	Avg	3.9841E+03	3.1964E+03	3.1813E+03	3.1207E+03	3.1303E+03	3.1309E+03
	Std	2.2522E+02	8.9891E+01	7.5969E+01	4.3549E+01	6.7882E+01	6.8614E+01
	Rank	6	5	4	1	2	3
F28	Best	4.0254E+03	3.7613E+03	3.8203E+03	3.7581E+03	3.7938E+03	3.8035E+03
	Avg	4.6093E+03	4.2202E+03	4.1344E+03	4.0753E+03	4.0308E+03	4.0468E+03
	Std	3.0880E+02	2.4769E+02	2.6737E+02	2.7452E+02	2.1534E+02	2.0096E+02
	Rank	6	5	4	3	1	2
F29	Best	1.8822E+06	3.3487E+03	3.5009E+03	3.4737E+03	3.5120E+03	3.4586E+03
	Avg	2.0801E+07	3.5896E+03	3.7655E+03	3.9714E+03	5.0017E+03	3.8130E+03
	Std	2.2612E+07	1.6874E+02	4.2749E+02	8.5721E+02	3.4195E+03	3.5703E+02
	Rank	6	1	2	4	5	3

**Table A7:** Experiments comparing EECO with different parameter as (50D).

No.	Index	ECO	a = 0.5	a = 0.6	a = 0.7	a = 0.8	a = 0.9
F1	Best	1.3982E+10	1.0000E+02	1.0000E+02	1.0000E+02	1.0000E+02	1.0000E+02
	Avg	2.7507E+10	1.0143E+02	1.0000E+02	1.0000E+02	1.0000E+02	1.0000E+02
	Std	1.2295E+10	3.3546E+00	3.5315E-04	1.1651E-06	4.1379E-06	8.7994E-06
	Rank	6	5	4	1	2	3
F2	Best	6.4697E+04	3.0000E+02	3.0000E+02	3.0000E+02	3.0000E+02	3.0000E+02
	Avg	1.0822E+05	3.0000E+02	3.0000E+02	3.0000E+02	3.0000E+02	3.0000E+02
	Std	3.8466E+04	2.6790E-05	2.2644E-05	2.4328E-05	2.4194E-05	3.3586E-03
	Rank	6	2	1	3	4	5
F3	Best	2.2968E+03	4.0000E+02	4.0000E+02	4.0000E+02	4.0000E+02	4.0000E+02
	Avg	4.6065E+03	4.0120E+02	4.0040E+02	4.0120E+02	4.0120E+02	4.0177E+02
	Std	2.4371E+03	1.9257E+00	1.2607E+00	1.9257E+00	1.9257E+00	1.9810E+00
	Rank	6	2	1	4	3	5
F4	Best	8.5796E+02	7.4277E+02	7.2287E+02	7.4078E+02	7.3779E+02	7.3083E+02
	Avg	9.9192E+02	8.0356E+02	7.9759E+02	7.9600E+02	7.9550E+02	7.9351E+02
	Std	9.4684E+01	4.3602E+01	5.1335E+01	4.5307E+01	4.4688E+01	4.7362E+01
	Rank	6	5	4	3	2	1
F5	Best	6.5862E+02	6.4217E+02	6.4148E+02	6.4148E+02	6.4132E+02	6.4074E+02
	Avg	6.6684E+02	6.5081E+02	6.5000E+02	6.5021E+02	6.4969E+02	6.4974E+02
	Std	5.5643E+00	5.1802E+00	5.5751E+00	5.6253E+00	5.8729E+00	5.7551E+00
	Rank	6	5	3	4	1	2
F6	Best	1.4736E+03	1.0846E+03	1.0827E+03	1.0745E+03	1.0835E+03	1.0835E+03
	Avg	1.5961E+03	1.3883E+03	1.3649E+03	1.3545E+03	1.3414E+03	1.3453E+03
	Std	7.0588E+01	1.6236E+02	1.4387E+02	1.4172E+02	1.3758E+02	1.3872E+02
	Rank	6	5	4	3	1	2
F7	Best	1.2522E+03	1.0189E+03	1.0179E+03	1.0179E+03	1.0179E+03	1.0149E+03
	Avg	1.3018E+03	1.0951E+03	1.0945E+03	1.0928E+03	1.0900E+03	1.0890E+03
	Std	4.1265E+01	5.1674E+01	5.2721E+01	5.0550E+01	4.9384E+01	5.0054E+01
	Rank	6	5	4	3	2	1
F8	Best	1.7076E+04	6.1237E+03	6.2360E+03	6.2498E+03	6.2687E+03	6.2408E+03
	Avg	2.3943E+04	9.2604E+03	9.1744E+03	9.1777E+03	8.8459E+03	8.8528E+03
	Std	4.7473E+03	1.7466E+03	1.7814E+03	1.7938E+03	1.9677E+03	1.9827E+03
	Rank	6	5	3	4	1	2
F9	Best	9.4499E+03	6.7913E+03	6.6107E+03	6.2585E+03	6.6329E+03	6.2913E+03
	Avg	1.2865E+04	8.2946E+03	8.2973E+03	8.2658E+03	8.1524E+03	8.0868E+03
	Std	1.4205E+03	7.3038E+02	9.3217E+02	1.0069E+03	9.0067E+02	9.7949E+02
	Rank	6	4	5	3	2	1
F10	Best	2.6975E+03	1.1915E+03	1.2005E+03	1.2075E+03	1.2293E+03	1.2383E+03
	Avg	7.5828E+03	1.2857E+03	1.2942E+03	1.2936E+03	1.3034E+03	1.3032E+03
	Std	4.1000E+03	5.8890E+01	6.5701E+01	6.4788E+01	4.8903E+01	5.4899E+01
	Rank	6	1	3	2	5	4
F11	Best	9.4040E+08	2.6603E+03	3.4740E+03	3.7836E+03	2.7355E+03	3.2915E+03
	Avg	3.1306E+09	4.5399E+03	4.5065E+03	5.0763E+03	6.4612E+03	8.1929E+03
	Std	1.6180E+09	1.0631E+03	8.1147E+02	7.7520E+02	6.1899E+03	7.0498E+03
	Rank	6	2	1	3	4	5
F12	Best	3.0077E+07	2.7646E+03	2.3687E+03	2.6882E+03	2.4087E+03	2.7124E+03
	Avg	2.9633E+08	3.8901E+03	3.4318E+03	4.4038E+03	3.8809E+03	5.7969E+03
	Std	3.3853E+08	7.9627E+02	7.1599E+02	2.0553E+03	1.0482E+03	3.8287E+03
	Rank	6	3	1	4	2	5
F13	Best	3.4667E+04	1.6472E+03	1.6149E+03	1.6205E+03	1.6794E+03	1.5158E+03
	Avg	7.6567E+05	1.7621E+03	1.7951E+03	1.7619E+03	1.8328E+03	2.2929E+03
	Std	9.8799E+05	1.1382E+02	1.2679E+02	1.0739E+02	1.1478E+02	8.5012E+02
	Rank	6	2	3	1	4	5
F14	Best	7.2450E+05	1.7850E+03	1.6544E+03	1.7091E+03	1.6747E+03	1.9018E+03
	Avg	4.9169E+07	1.9577E+03	2.0519E+03	2.0958E+03	2.2319E+03	2.6003E+03
	Std	8.5723E+07	1.9572E+02	4.8844E+02	4.7568E+02	4.1193E+02	1.1742E+03
	Rank	6	1	2	3	4	5
F15	Best	3.6363E+03	2.6617E+03	2.6616E+03	2.6616E+03	2.6617E+03	2.6616E+03
	Avg	4.4666E+03	3.6028E+03	3.5435E+03	3.4762E+03	3.4990E+03	3.5221E+03
	Std	6.9770E+02	5.3232E+02	5.7077E+02	5.7146E+02	5.7047E+02	5.6806E+02
	Rank	6	5	4	1	2	3
F16	Best	3.3004E+03	3.0542E+03	3.0266E+03	3.0247E+03	3.0066E+03	3.0525E+03
	Avg	3.9210E+03	3.4643E+03	3.4666E+03	3.4245E+03	3.4492E+03	3.4575E+03
	Std	5.2555E+02	2.8083E+02	2.8539E+02	2.2888E+02	2.6341E+02	2.4386E+02
	Rank	6	4	5	1	2	3
F17	Best	4.7151E+05	1.8750E+03	1.9655E+03	1.8931E+03	1.8912E+03	3.2855E+03
	Avg	6.7951E+06	2.0366E+03	2.0383E+03	1.9959E+03	2.3766E+03	6.9861E+03
	Std	6.2519E+06	1.3461E+02	5.3906E+01	7.2524E+01	3.9751E+02	3.9086E+03
	Rank	6	2	3	1	4	5
F18	Best	2.8826E+05	2.0910E+03	2.0533E+03	2.2315E+03	2.3956E+03	2.3188E+03
	Avg	2.3034E+07	2.3706E+03	2.4029E+03	2.7873E+03	3.6576E+03	8.0225E+03
	Std	5.1801E+07	2.7348E+02	2.6305E+02	6.1208E+02	1.6342E+03	5.9731E+03
	Rank	6	1	2	3	4	5
F19	Best	3.0192E+03	2.5548E+03	2.5459E+03	2.5669E+03	2.5479E+03	2.5469E+03
	Avg	3.5627E+03	3.1917E+03	3.1320E+03	3.1304E+03	3.1251E+03	3.1297E+03
	Std	3.1123E+02	4.2055E+02	4.0493E+02	4.2369E+02	4.2248E+02	4.1809E+02
	Rank	6	5	4	3	1	2
F20	Best	2.6066E+03	2.4536E+03	2.4540E+03	2.4566E+03	2.4566E+03	2.4585E+03
	Avg	2.7480E+03	2.5709E+03	2.5729E+03	2.5726E+03	2.5715E+03	2.5716E+03
	Std	9.5071E+01	6.2980E+01	6.3498E+01	6.2273E+01	6.3547E+01	6.2944E+01
	Rank	6	1	5	4	2	3
F21	Best	1.3152E+04	8.3074E+03	8.1761E+03	8.3607E+03	8.3586E+03	8.1970E+03
	Avg	1.4535E+04	9.4961E+03	9.4112E+03	9.3560E+03	9.4815E+03	9.6024E+03
	Std	8.9691E+02	7.6530E+02	7.4955E+02	6.5483E+02	8.0954E+02	8.7011E+02
	Rank	6	4	2	1	3	5
F22	Best	3.3001E+03	3.0622E+03	3.0162E+03	3.0471E+03	3.0050E+03	3.0087E+03
	Avg	3.3894E+03	3.1991E+03	3.1847E+03	3.1749E+03	3.1461E+03	3.1415E+03
	Std	8.4934E+01	1.0798E+02	1.1520E+02	1.1480E+02	1.2839E+02	1.3008E+02
	Rank	6	5	4	3	2	1
F23	Best	3.4416E+03	3.1810E+03	3.1482E+03	3.1462E+03	3.0585E+03	3.0920E+03
	Avg	3.5253E+03	3.3530E+03	3.2986E+03	3.2880E+03	3.2715E+03	3.2577E+03
	Std	7.0905E+01	1.2242E+02	1.2132E+02	1.2895E+02	1.4544E+02	1.4702E+02
	Rank	6	5	4	3	2	1
F24	Best	4.5928E+03	2.9312E+03	2.9313E+03	2.9313E+03	2.9313E+03	2.9313E+03
	Avg	6.1549E+03	2.9656E+03	2.9703E+03	2.9766E+03	2.9557E+03	2.9596E+03
	Std	1.1810E+03	3.1874E+01	1.9223E+01	3.0393E+01	2.8872E+01	1.9907E+01
	Rank	6	3	4	5	1	2
F25	Best	9.3476E+03	2.9000E+03	2.9000E+03	2.9000E+03	2.9000E+03	2.9000E+03
	Avg	1.1153E+04	8.0455E+03	8.2574E+03	8.1936E+03	7.7180E+03	7.9819E+03
	Std	1.2482E+03	2.0987E+03	2.1142E+03	2.1338E+03	2.6686E+03	2.2444E+03
	Rank	6	3	5	4	1	2
F26	Best	3.5181E+03	3.2000E+03	3.2000E+03	3.2000E+03	3.2000E+03	3.2000E+03
	Avg	3.8122E+03	3.2000E+03	3.2000E+03	3.2000E+03	3.2171E+03	3.2000E+03
	Std	2.4505E+02	1.5786E-04	2.4357E-04	1.6338E-04	5.4060E+01	1.2729E-04
	Rank	6	2	1	3	5	4
F27	Best	5.2264E+03	3.3000E+03	3.3000E+03	3.3000E+03	3.3000E+03	3.3000E+03
	Avg	6.7929E+03	3.3000E+03	3.3000E+03	3.3000E+03	3.3000E+03	3.3000E+03
	Std	1.5797E+03	3.2608E-04	4.0006E-04	3.2784E-04	2.5321E-04	2.7928E-04
	Rank	6	1	5	3	4	2
F28	Best	5.3623E+03	4.1821E+03	4.3256E+03	4.1128E+03	4.0205E+03	3.9143E+03
	Avg	6.6733E+03	4.7937E+03	4.7995E+03	4.7584E+03	4.6540E+03	4.6277E+03
	Std	8.6922E+02	3.3154E+02	3.0021E+02	3.5240E+02	3.6891E+02	4.5449E+02
	Rank	6	4	5	3	2	1
F29	Best	6.0193E+07	3.7396E+03	3.8566E+03	3.6930E+03	3.4871E+03	3.9225E+03
	Avg	1.8378E+08	5.7981E+03	6.9528E+03	4.6114E+03	4.6278E+03	8.5123E+03
	Std	1.7870E+08	4.4484E+03	4.8510E+03	1.0670E+03	1.0772E+03	6.5112E+03
	Rank	6	3	4	1	2	5

**Table A8:** Experiments comparing EECO with different parameter as (100D).

No.	Index	ECO	a = 0.5	a = 0.6	a = 0.7	a = 0.8	a = 0.9
F1	Best	1.0516E+11	2.7355E+03	2.0362E+03	2.0566E+03	1.2195E+02	1.9212E+02
	Avg	1.1103E+11	9.8305E+03	7.2179E+03	8.1565E+03	9.1249E+03	8.7906E+03
	Std	4.9348E+09	4.8534E+03	3.9671E+03	4.6948E+03	8.5787E+03	9.8954E+03
	Rank	6	5	1	2	4	3
F2	Best	2.5917E+05	3.9492E+04	5.1115E+04	3.1963E+04	3.7096E+04	4.9098E+04
	Avg	2.8050E+05	5.7541E+04	5.4596E+04	5.0453E+04	5.7097E+04	6.0791E+04
	Std	2.5113E+04	3.0564E+04	2.8414E+03	1.6305E+04	1.7974E+04	1.2574E+04
	Rank	6	4	2	1	3	5
F3	Best	1.1491E+04	5.8216E+02	5.6703E+02	5.7384E+02	5.7172E+02	5.7646E+02
	Avg	1.6719E+04	6.0899E+02	5.9268E+02	6.0577E+02	6.0567E+02	6.2239E+02
	Std	8.1866E+03	2.7027E+01	3.5782E+01	3.3935E+01	3.5256E+01	3.0835E+01
	Rank	6	4	1	3	2	5
F4	Best	1.5748E+03	1.1686E+03	1.1626E+03	1.1746E+03	1.1666E+03	1.1696E+03
	Avg	1.6415E+03	1.2064E+03	1.2069E+03	1.2134E+03	1.2034E+03	1.2044E+03
	Std	4.5651E+01	4.0152E+01	4.8535E+01	4.5135E+01	4.4628E+01	4.3754E+01
	Rank	6	3	4	5	1	2
F5	Best	6.7534E+02	6.5439E+02	6.5321E+02	6.5284E+02	6.5310E+02	6.5332E+02
	Avg	6.7727E+02	6.5835E+02	6.5808E+02	6.5779E+02	6.5799E+02	6.5814E+02
	Std	1.9568E+00	4.6156E+00	5.1737E+00	5.7479E+00	5.6611E+00	5.4908E+00
	Rank	6	5	3	1	2	4
F6	Best	3.1609E+03	2.5121E+03	2.4678E+03	2.5250E+03	2.4412E+03	2.4539E+03
	Avg	3.2687E+03	2.7763E+03	2.7505E+03	2.7037E+03	2.6838E+03	2.6903E+03
	Std	1.3907E+02	2.2560E+02	2.4892E+02	1.8362E+02	2.1687E+02	2.0897E+02
	Rank	6	5	4	3	1	2
F7	Best	1.8844E+03	1.4955E+03	1.5074E+03	1.5074E+03	1.5134E+03	1.5114E+03
	Avg	2.0902E+03	1.5517E+03	1.5402E+03	1.5502E+03	1.5502E+03	1.5502E+03
	Std	1.5760E+02	5.5990E+01	4.6310E+01	4.3130E+01	4.4397E+01	4.9924E+01
	Rank	6	5	1	3	4	2
F8	Best	4.8102E+04	1.6571E+04	1.6601E+04	1.6599E+04	1.6582E+04	1.6612E+04
	Avg	5.6594E+04	1.9584E+04	2.1672E+04	2.1686E+04	1.9625E+04	1.9724E+04
	Std	6.9367E+03	2.0606E+03	5.3329E+03	5.3668E+03	2.0735E+03	2.1294E+03
	Rank	6	1	4	5	2	3
F9	Best	2.5800E+04	1.5260E+04	1.5136E+04	1.5334E+04	1.5201E+04	1.5195E+04
	Avg	2.7131E+04	1.6361E+04	1.5928E+04	1.6271E+04	1.6417E+04	1.6592E+04
	Std	1.5785E+03	1.1549E+03	8.6530E+02	8.8180E+02	1.1921E+03	1.3406E+03
	Rank	6	3	1	2	4	5
F10	Best	5.0265E+04	2.2440E+03	2.2198E+03	2.1987E+03	2.1024E+03	2.1668E+03
	Avg	8.1274E+04	2.2856E+03	2.2876E+03	2.3552E+03	2.3643E+03	2.3560E+03
	Std	3.9368E+04	5.4685E+01	9.2419E+01	1.7630E+02	3.5223E+02	1.8517E+02
	Rank	6	1	2	3	5	4
F11	Best	1.9040E+10	6.1180E+05	6.0306E+05	6.6267E+05	3.7465E+05	6.2566E+05
	Avg	2.7304E+10	1.0551E+06	9.0999E+05	8.1703E+05	9.2509E+05	1.0787E+06
	Std	6.5837E+09	3.3217E+05	3.0758E+05	2.0875E+05	5.4639E+05	7.6097E+05
	Rank	6	4	2	1	3	5
F12	Best	4.4658E+08	6.6312E+03	6.4242E+03	6.6649E+03	6.4993E+03	6.7171E+03
	Avg	1.7600E+09	8.8200E+03	8.7041E+03	8.6476E+03	8.4896E+03	8.5395E+03
	Std	1.0977E+09	3.1820E+03	3.2192E+03	3.1393E+03	2.9186E+03	3.0369E+03
	Rank	6	5	4	3	1	2
F13	Best	2.4685E+06	2.5915E+04	2.7599E+04	3.4168E+04	2.7228E+04	2.4253E+04
	Avg	9.9605E+06	3.9322E+04	4.3286E+04	4.4772E+04	4.3829E+04	3.9653E+04
	Std	1.1653E+07	2.3415E+04	1.7869E+04	1.3445E+04	1.7946E+04	1.1935E+04
	Rank	6	1	3	5	4	2
F14	Best	8.5045E+07	2.5813E+03	2.3992E+03	2.2810E+03	2.4414E+03	2.3055E+03
	Avg	1.4398E+08	1.1474E+04	1.2266E+04	1.2489E+04	1.2269E+04	1.2220E+04
	Std	6.2725E+07	1.3058E+04	1.5074E+04	1.5458E+04	1.5243E+04	1.5061E+04
	Rank	6	1	3	5	4	2
F15	Best	8.9502E+03	5.0165E+03	4.9661E+03	5.0544E+03	5.0358E+03	4.9132E+03
	Avg	9.3818E+03	5.4519E+03	5.4298E+03	5.4858E+03	5.4801E+03	5.4861E+03
	Std	4.4861E+02	4.2214E+02	3.9786E+02	4.4746E+02	3.9855E+02	4.4726E+02
	Rank	6	2	1	4	3	5
F16	Best	6.3541E+03	5.3851E+03	5.3515E+03	5.5186E+03	5.3938E+03	5.5299E+03
	Avg	8.3109E+03	6.3625E+03	6.2110E+03	6.7242E+03	6.3913E+03	6.4490E+03
	Std	2.3441E+03	7.6691E+02	7.6225E+02	1.0471E+03	7.5598E+02	6.9518E+02
	Rank	6	2	1	5	3	4
F17	Best	1.8859E+06	3.5457E+04	1.7800E+04	4.4354E+04	4.2100E+04	3.9002E+04
	Avg	7.1368E+06	5.4331E+04	5.5221E+04	7.1847E+04	5.1266E+04	6.4633E+04
	Std	4.2842E+06	2.0149E+04	2.7475E+04	2.8583E+04	1.0155E+04	1.8453E+04
	Rank	6	2	3	5	1	4
F18	Best	1.6028E+08	3.9116E+03	3.2475E+03	3.2101E+03	3.1791E+03	2.8712E+03
	Avg	4.5686E+08	6.8990E+03	6.5667E+03	6.8000E+03	6.6747E+03	5.9845E+03
	Std	3.6604E+08	5.3093E+03	6.1591E+03	5.9971E+03	5.4749E+03	5.5463E+03
	Rank	6	5	2	4	3	1
F19	Best	4.7506E+03	4.5373E+03	4.5213E+03	4.5196E+03	4.5386E+03	4.5325E+03
	Avg	5.9860E+03	5.0542E+03	4.9895E+03	5.0466E+03	5.0555E+03	4.9888E+03
	Std	8.9336E+02	6.2926E+02	5.3692E+02	5.9563E+02	5.9846E+02	5.8615E+02
	Rank	6	4	2	3	5	1
F20	Best	3.5372E+03	3.0067E+03	2.9870E+03	2.9957E+03	3.0169E+03	2.9867E+03
	Avg	3.6555E+03	3.1283E+03	3.1281E+03	3.1226E+03	3.1317E+03	3.1161E+03
	Std	9.5124E+01	8.2571E+01	9.5133E+01	8.8523E+01	7.6772E+01	8.8214E+01
	Rank	6	4	3	2	5	1
F21	Best	2.8975E+04	1.7786E+04	1.8288E+04	1.7665E+04	1.7295E+04	1.6959E+04
	Avg	3.0308E+04	1.9765E+04	1.9873E+04	1.9803E+04	1.9320E+04	1.9476E+04
	Std	1.1579E+03	1.6795E+03	1.5984E+03	1.8522E+03	1.6695E+03	2.0595E+03
	Rank	6	3	5	4	1	2
F22	Best	4.0146E+03	3.8719E+03	3.8307E+03	3.8316E+03	3.8335E+03	3.8422E+03
	Avg	4.4003E+03	4.0090E+03	3.9492E+03	3.9820E+03	3.9667E+03	3.9331E+03
	Std	2.7683E+02	1.4931E+02	1.3990E+02	1.6874E+02	1.7394E+02	1.3387E+02
	Rank	6	5	2	4	3	1
F23	Best	4.8007E+03	4.4830E+03	4.3510E+03	4.4365E+03	4.3493E+03	4.3611E+03
	Avg	5.1692E+03	5.0090E+03	4.7597E+03	4.7822E+03	4.7365E+03	4.6987E+03
	Std	2.7420E+02	4.6767E+02	3.1209E+02	3.6610E+02	3.8043E+02	3.7742E+02
	Rank	6	5	3	4	2	1
F24	Best	9.5292E+03	3.1168E+03	3.0618E+03	3.2057E+03	3.1789E+03	3.1984E+03
	Avg	1.1884E+04	3.2610E+03	3.2253E+03	3.2862E+03	3.2670E+03	3.2563E+03
	Std	2.4724E+03	1.2330E+02	1.2207E+02	8.8634E+01	1.0082E+02	4.3313E+01
	Rank	6	3	1	5	4	2
F25	Best	2.0470E+04	1.4903E+04	1.4342E+04	1.4437E+04	1.4539E+04	1.4504E+04
	Avg	2.5238E+04	2.0051E+04	2.0261E+04	2.0388E+04	2.0108E+04	2.0188E+04
	Std	3.9562E+03	4.6741E+03	5.3808E+03	5.5320E+03	5.2042E+03	5.3760E+03
	Rank	6	1	4	5	2	3
F26	Best	4.0171E+03	3.2000E+03	3.2000E+03	3.2000E+03	3.2000E+03	3.3814E+03
	Avg	4.4848E+03	3.7430E+03	3.3981E+03	3.4600E+03	3.6156E+03	3.7416E+03
	Std	6.6520E+02	6.4311E+02	3.9622E+02	3.0224E+02	4.9520E+02	3.2470E+02
	Rank	6	5	1	2	3	4
F27	Best	1.0026E+04	3.2951E+03	3.2951E+03	3.2951E+03	3.3164E+03	3.3173E+03
	Avg	1.7443E+04	3.2990E+03	3.3028E+03	3.3297E+03	3.3379E+03	3.3287E+03
	Std	5.0251E+03	6.5512E+00	1.4984E+01	2.6586E+01	2.0147E+01	9.3743E+00
	Rank	6	1	2	4	5	3
F28	Best	1.2195E+04	6.3031E+03	6.2190E+03	6.2987E+03	5.9239E+03	5.6597E+03
	Avg	1.4170E+04	7.2658E+03	7.0021E+03	7.0854E+03	6.8564E+03	6.9274E+03
	Std	2.8708E+03	9.0276E+02	6.3775E+02	9.4837E+02	1.1374E+03	1.1567E+03
	Rank	6	5	3	4	1	2
F29	Best	5.5469E+08	4.3864E+03	4.6825E+03	4.7552E+03	5.5174E+03	5.0398E+03
	Avg	7.5685E+08	7.8931E+03	8.1504E+03	8.0196E+03	8.7649E+03	8.5681E+03
	Std	1.6456E+08	4.4293E+03	4.9147E+03	5.7282E+03	5.7765E+03	5.7726E+03
	Rank	6	1	3	2	5	4

**Table A9:** Experiments comparing EECO with different strategies (10D).

No.	Index	ECO	ECO-R	ECO-P	ECO-T	ECO-RP	ECO-RT	ECO-PT	EECO
F1	Best	6.3568E+06	3.4081E+02	9.4699E+02	6.6813E+04	1.0000E+02	1.0000E+02	1.0000E+02	1.0000E+02
	Avg	1.5632E+08	8.6949E+04	1.7517E+04	4.1678E+06	5.7070E+02	1.0000E+02	1.0019E+02	1.0000E+02
	Std	3.1359E+08	2.0888E+05	4.2886E+04	6.2632E+06	1.3967E+03	2.1836E-07	7.2283E-01	0.0000E+00
	Rank	8	6	5	7	4	2	3	1
F2	Best	7.2490E+02	3.0000E+02	3.0001E+02	3.7683E+02	3.0000E+02	3.0000E+02	3.0000E+02	3.0000E+02
	Avg	3.1268E+03	3.0342E+02	3.0347E+02	1.0111E+03	3.0000E+02	3.0000E+02	3.0000E+02	3.0000E+02
	Std	2.1573E+03	1.0140E+01	1.0456E+01	5.8115E+02	9.0236E-06	7.4740E-12	9.9336E-11	0.0000E+00
	Rank	8	5	6	7	4	2	3	1
F3	Best	4.0663E+02	4.0270E+02	4.0346E+02	4.0175E+02	4.0000E+02	4.0000E+02	4.0000E+02	4.0000E+02
	Avg	4.2652E+02	4.0478E+02	4.0503E+02	4.0841E+02	4.0300E+02	4.0000E+02	4.0000E+02	4.0000E+02
	Std	2.3682E+01	1.2658E+00	9.7697E-01	7.0677E+00	1.4796E+00	1.2855E-12	1.7717E-13	4.1355E-13
	Rank	8	5	6	7	4	3	1	1
F4	Best	5.1504E+02	5.0873E+02	5.1582E+02	5.1087E+02	5.0106E+02	5.0696E+02	5.0995E+02	5.0199E+02
	Avg	5.4079E+02	5.1957E+02	5.2575E+02	5.2398E+02	5.0561E+02	5.2912E+02	5.2136E+02	5.0451E+02
	Std	1.4548E+01	8.1612E+00	5.6800E+00	7.1122E+00	3.2460E+00	1.8853E+01	7.4253E+00	1.7983E+00
	Rank	8	3	6	5	2	7	4	1
F5	Best	6.0731E+02	6.0007E+02	6.0018E+02	6.0852E+02	6.0000E+02	6.0516E+02	6.0720E+02	6.0000E+02
	Avg	6.2141E+02	6.0133E+02	6.0043E+02	6.1743E+02	6.0003E+02	6.1352E+02	6.1785E+02	6.0001E+02
	Std	9.6906E+00	3.4964E+00	3.6358E-01	8.0170E+00	7.1318E-02	5.9790E+00	7.3533E+00	3.7882E-02
	Rank	8	4	3	6	2	5	7	1
F6	Best	7.2950E+02	7.1901E+02	7.2143E+02	7.3110E+02	7.1208E+02	7.1789E+02	7.2024E+02	7.1089E+02
	Avg	7.6287E+02	7.3210E+02	7.3799E+02	7.5131E+02	7.1841E+02	7.4524E+02	7.4477E+02	7.1422E+02
	Std	1.8573E+01	7.9956E+00	7.6167E+00	1.3791E+01	5.0688E+00	1.3200E+01	1.3906E+01	3.3408E+00
	Rank	8	3	4	7	2	6	5	1
F7	Best	8.1459E+02	8.1186E+02	8.1230E+02	8.1247E+02	8.0100E+02	8.0995E+02	8.0995E+02	8.0199E+02
	Avg	8.3687E+02	8.2398E+02	8.2517E+02	8.2672E+02	8.0576E+02	8.2235E+02	8.2341E+02	8.0829E+02
	Std	9.3431E+00	8.1097E+00	5.5161E+00	8.4988E+00	3.8371E+00	9.1874E+00	9.6601E+00	4.8839E+00
	Rank	8	5	6	7	1	3	4	2
F8	Best	9.2354E+02	9.0001E+02	9.0000E+02	9.2033E+02	9.0000E+02	9.1734E+02	9.2501E+02	9.0000E+02
	Avg	1.0787E+03	9.0008E+02	9.0073E+02	1.0804E+03	9.0000E+02	1.0789E+03	1.0750E+03	9.0000E+02
	Std	1.2133E+02	1.2950E-01	1.8366E+00	1.4999E+02	4.7727E-08	1.4738E+02	1.3488E+02	0.0000E+00
	Rank	6	3	4	8	2	7	5	1
F9	Best	1.6251E+03	1.9746E+03	1.7746E+03	1.5390E+03	1.1735E+03	1.2540E+03	1.4894E+03	1.3769E+03
	Avg	2.0675E+03	2.2216E+03	2.2668E+03	2.0639E+03	2.0169E+03	1.7952E+03	1.9113E+03	1.6773E+03
	Std	3.3508E+02	2.1401E+02	2.8930E+02	2.4145E+02	3.4517E+02	2.6213E+02	1.7724E+02	1.9887E+02
	Rank	6	7	8	5	4	2	3	1
F10	Best	1.1602E+03	1.1055E+03	1.1035E+03	1.1269E+03	1.1008E+03	1.1209E+03	1.1249E+03	1.1010E+03
	Avg	1.2608E+03	1.1149E+03	1.1155E+03	1.1905E+03	1.1074E+03	1.1761E+03	1.1517E+03	1.1069E+03
	Std	9.8558E+01	9.8691E+00	1.2798E+01	1.1368E+02	4.5889E+00	4.5928E+01	3.4474E+01	6.5809E+00
	Rank	8	3	4	7	2	6	5	1
F11	Best	9.6759E+04	1.5531E+03	1.5929E+03	7.8112E+03	1.3209E+03	1.3186E+03	1.3186E+03	1.3186E+03
	Avg	4.9010E+06	6.5012E+03	4.5646E+05	5.2419E+05	2.7239E+05	2.1391E+03	1.5788E+03	1.5028E+03
	Std	6.3820E+06	1.0932E+04	1.7416E+06	6.3214E+05	1.0482E+06	1.5224E+03	1.9574E+02	2.0539E+02
	Rank	8	4	6	7	5	3	2	1
F12	Best	1.9964E+03	1.3197E+03	1.3372E+03	1.9635E+03	1.3068E+03	1.4370E+03	1.3984E+03	1.3054E+03
	Avg	1.5972E+04	1.4547E+03	1.5285E+03	5.1567E+03	1.4662E+03	1.8507E+03	1.8035E+03	1.4125E+03
	Std	1.8296E+04	1.2786E+02	2.2942E+02	3.9474E+03	1.4450E+02	3.1223E+02	3.3130E+02	1.3790E+02
	Rank	8	2	4	7	3	6	5	1
F13	Best	1.4708E+03	1.4134E+03	1.4164E+03	1.4568E+03	1.4031E+03	1.4341E+03	1.4255E+03	1.4010E+03
	Avg	1.5422E+03	1.4266E+03	1.4281E+03	1.4852E+03	1.4174E+03	1.4893E+03	1.4674E+03	1.4181E+03
	Std	8.5245E+01	4.7795E+00	6.9769E+00	2.4760E+01	9.8792E+00	3.9156E+01	3.0306E+01	1.2380E+01
	Rank	8	3	4	6	1	7	5	2
F14	Best	1.6616E+03	1.5029E+03	1.5032E+03	1.6092E+03	1.5010E+03	1.5035E+03	1.5111E+03	1.5012E+03
	Avg	2.5911E+03	1.5243E+03	1.5204E+03	1.8519E+03	1.5188E+03	1.6003E+03	1.5924E+03	1.5070E+03
	Std	1.0180E+03	2.2785E+01	1.8269E+01	2.4737E+02	1.8296E+01	6.0997E+01	7.2889E+01	7.3589E+00
	Rank	8	4	3	7	2	6	5	1
F15	Best	1.6103E+03	1.6081E+03	1.6176E+03	1.6546E+03	1.6018E+03	1.6014E+03	1.6033E+03	1.6006E+03
	Avg	1.7834E+03	1.6557E+03	1.6565E+03	1.7715E+03	1.6153E+03	1.6999E+03	1.7159E+03	1.6043E+03
	Std	1.0502E+02	3.6519E+01	3.1351E+01	9.2316E+01	3.3264E+01	8.5837E+01	1.0326E+02	5.0260E+00
	Rank	8	3	4	7	2	5	6	1
F16	Best	1.7553E+03	1.7360E+03	1.7485E+03	1.7457E+03	1.7302E+03	1.7308E+03	1.7312E+03	1.7017E+03
	Avg	1.7989E+03	1.7768E+03	1.7701E+03	1.7671E+03	1.7400E+03	1.7631E+03	1.7566E+03	1.7299E+03
	Std	4.2116E+01	2.2765E+01	1.4271E+01	1.4216E+01	1.0717E+01	3.0365E+01	1.3362E+01	1.9279E+01
	Rank	8	7	6	5	2	4	3	1
F17	Best	2.8667E+03	1.8246E+03	1.8271E+03	2.5924E+03	1.8212E+03	1.8265E+03	1.8252E+03	1.8020E+03
	Avg	2.0116E+04	1.9457E+03	1.8840E+03	1.9664E+04	1.8541E+03	1.8945E+03	1.9137E+03	1.8248E+03
	Std	1.5720E+04	2.5294E+02	8.6589E+01	1.2765E+04	7.5336E+01	7.5361E+01	8.0518E+01	1.3687E+01
	Rank	8	6	3	7	2	4	5	1
F18	Best	1.9546E+03	1.9045E+03	1.9040E+03	1.9420E+03	1.9006E+03	1.9150E+03	1.9090E+03	1.9001E+03
	Avg	3.0823E+03	1.9068E+03	1.9061E+03	2.1495E+03	1.9032E+03	1.9786E+03	1.9722E+03	1.9023E+03
	Std	1.8128E+03	2.4554E+00	2.0609E+00	2.5198E+02	2.2375E+00	4.0846E+01	4.6082E+01	1.4866E+00
	Rank	8	4	3	7	2	6	5	1
F19	Best	2.0621E+03	2.0468E+03	2.0591E+03	2.0313E+03	2.0210E+03	2.0390E+03	2.0316E+03	2.0003E+03
	Avg	2.1270E+03	2.0950E+03	2.0955E+03	2.1223E+03	2.0391E+03	2.0841E+03	2.1185E+03	2.0217E+03
	Std	4.6113E+01	3.6929E+01	3.5544E+01	5.8736E+01	1.5298E+01	3.6869E+01	5.7480E+01	1.2913E+01
	Rank	8	4	5	7	2	3	6	1
F20	Best	2.2086E+03	2.2036E+03	2.2066E+03	2.2028E+03	2.2000E+03	2.2024E+03	2.2022E+03	2.2000E+03
	Avg	2.2536E+03	2.2950E+03	2.2943E+03	2.2578E+03	2.2782E+03	2.2599E+03	2.2468E+03	2.2711E+03
	Std	5.2251E+01	4.6092E+01	4.1245E+01	6.1546E+01	4.7641E+01	6.3311E+01	6.4381E+01	5.0488E+01
	Rank	2	8	7	3	6	4	1	5
F21	Best	2.2577E+03	2.2118E+03	2.2121E+03	2.2469E+03	2.2116E+03	2.2116E+03	2.2457E+03	2.2000E+03
	Avg	2.3184E+03	2.2907E+03	2.2846E+03	2.3068E+03	2.2882E+03	2.2966E+03	2.3037E+03	2.2882E+03
	Std	2.8828E+01	3.2387E+01	3.3724E+01	1.7493E+01	3.1126E+01	3.6065E+01	1.7765E+01	3.1550E+01
	Rank	8	4	1	7	2	5	6	3
F22	Best	2.6198E+03	2.6031E+03	2.6074E+03	2.6139E+03	2.6000E+03	2.6115E+03	2.6098E+03	2.6043E+03
	Avg	2.6346E+03	2.6188E+03	2.6225E+03	2.6262E+03	2.6104E+03	2.6239E+03	2.6200E+03	2.6083E+03
	Std	1.9097E+01	8.1275E+00	1.2546E+01	9.3875E+00	6.1417E+00	9.8880E+00	6.8405E+00	2.7440E+00
	Rank	8	3	5	7	2	6	4	1
F23	Best	2.5475E+03	2.6093E+03	2.6808E+03	2.5317E+03	2.5000E+03	2.5000E+03	2.5000E+03	2.5000E+03
	Avg	2.7375E+03	2.7361E+03	2.7449E+03	2.7127E+03	2.7120E+03	2.7204E+03	2.7009E+03	2.7058E+03
	Std	6.7204E+01	3.5875E+01	1.8709E+01	8.4827E+01	6.9021E+01	9.1787E+01	1.0415E+02	8.3745E+01
	Rank	7	6	8	4	3	5	1	2
F24	Best	2.9133E+03	2.8977E+03	2.8977E+03	2.8990E+03	2.8981E+03	2.8978E+03	2.8981E+03	2.8977E+03
	Avg	2.9530E+03	2.9247E+03	2.9286E+03	2.9479E+03	2.9487E+03	2.9363E+03	2.9407E+03	2.9366E+03
	Std	2.3641E+01	3.4790E+01	3.5185E+01	4.4075E+01	3.4622E+01	2.3095E+01	4.2636E+01	2.9542E+01
	Rank	8	1	2	6	7	3	5	4
F25	Best	2.9206E+03	2.9002E+03	2.9002E+03	2.9055E+03	2.8000E+03	2.6000E+03	2.8000E+03	2.9000E+03
	Avg	3.1026E+03	2.9436E+03	2.9230E+03	3.0276E+03	2.9265E+03	3.0110E+03	3.0082E+03	2.9230E+03
	Std	1.0755E+02	5.2072E+01	3.9557E+01	8.5862E+01	6.6434E+01	1.8356E+02	1.0894E+02	5.3723E+01
	Rank	8	4	1	7	3	6	5	2
F26	Best	3.0914E+03	3.0891E+03	3.0894E+03	3.0900E+03	3.0893E+03	3.0884E+03	3.0765E+03	3.0884E+03
	Avg	3.1013E+03	3.0927E+03	3.0991E+03	3.0950E+03	3.1013E+03	3.0945E+03	3.0911E+03	3.0959E+03
	Std	1.1701E+01	3.5382E+00	1.6515E+01	3.6588E+00	1.8993E+01	4.9097E+00	6.5488E+00	1.0550E+01
	Rank	7	2	6	4	8	3	1	5
F27	Best	3.1801E+03	3.1647E+03	3.1687E+03	3.1968E+03	3.2087E+03	3.1000E+03	3.1000E+03	3.1000E+03
	Avg	3.4163E+03	3.3593E+03	3.3706E+03	3.4279E+03	3.4421E+03	3.2530E+03	3.2478E+03	3.2558E+03
	Std	1.3588E+02	8.5758E+01	9.8072E+01	1.4533E+02	1.3197E+02	5.4080E+01	5.5797E+01	4.7561E+01
	Rank	6	4	5	7	8	2	1	3
F28	Best	3.1753E+03	3.1573E+03	3.1556E+03	3.1696E+03	3.1415E+03	3.1488E+03	3.1615E+03	3.1285E+03
	Avg	3.2381E+03	3.2076E+03	3.2025E+03	3.2541E+03	3.1693E+03	3.2309E+03	3.2427E+03	3.1616E+03
	Std	4.2469E+01	3.7233E+01	3.2331E+01	6.4757E+01	3.4182E+01	7.1347E+01	6.2455E+01	4.3286E+01
	Rank	6	4	3	8	2	5	7	1
F29	Best	1.9416E+04	3.6599E+03	3.7610E+03	7.4945E+03	3.5050E+03	3.2322E+03	3.2439E+03	3.2239E+03
	Avg	8.0401E+05	4.8655E+05	6.3152E+05	8.5133E+05	3.2142E+05	3.4133E+03	3.4298E+03	3.4510E+03
	Std	7.4313E+05	7.3926E+05	1.0372E+06	8.1339E+05	4.7485E+05	1.3867E+02	8.6260E+01	4.7789E+02
	Rank	7	5	6	8	4	1	2	3

**Table A10:** Experiments comparing EECO with different strategies (30D).

No.	Index	ECO	ECO-R	ECO-P	ECO-T	ECO-RP	ECO-RT	ECO-PT	EECO
F1	Best	1.5106E+09	1.2621E+07	2.8527E+07	1.9648E+08	4.3121E+05	1.0000E+02	1.0000E+02	1.0000E+02
	Avg	7.7062E+09	1.4281E+09	1.0271E+09	1.5388E+09	2.5736E+07	1.0003E+02	1.0000E+02	1.0000E+02
	Std	3.5527E+09	2.7110E+09	1.7790E+09	1.1116E+09	2.2270E+07	1.1957E-01	4.0902E-07	5.3627E-10
	Rank	8	6	5	7	4	3	2	1
F2	Best	3.1205E+04	8.7174E+02	1.9051E+03	1.2223E+04	1.0580E+03	3.0000E+02	3.0000E+02	3.0000E+02
	Avg	5.2052E+04	4.1547E+03	6.8564E+03	2.9623E+04	3.6491E+03	3.0000E+02	3.0000E+02	3.0000E+02
	Std	1.2534E+04	2.9907E+03	4.0857E+03	9.6754E+03	2.1574E+03	2.5582E-06	3.3100E-06	6.9834E-13
	Rank	8	5	6	7	4	2	3	1
F3	Best	7.6180E+02	5.1010E+02	4.9059E+02	5.6375E+02	4.8396E+02	4.0000E+02	4.0000E+02	4.0000E+02
	Avg	1.1569E+03	5.7615E+02	6.0134E+02	6.1614E+02	5.1568E+02	4.0292E+02	4.0186E+02	4.0186E+02
	Std	3.0717E+02	1.1645E+02	1.6387E+02	3.4472E+01	2.0865E+01	1.8248E+00	2.0587E+00	2.0587E+00
	Rank	8	5	6	7	4	3	2	1
F4	Best	6.5226E+02	5.7103E+02	6.4876E+02	6.1010E+02	5.3872E+02	5.6865E+02	5.9751E+02	5.1990E+02
	Avg	7.4363E+02	6.6984E+02	6.8004E+02	6.9766E+02	5.9287E+02	6.4029E+02	6.6788E+02	5.4245E+02
	Std	4.8611E+01	2.9971E+01	1.9930E+01	4.1417E+01	4.0202E+01	4.6932E+01	3.6561E+01	2.4699E+01
	Rank	8	5	6	7	2	3	4	1
F5	Best	6.2982E+02	6.0400E+02	6.0684E+02	6.3117E+02	6.0169E+02	6.2765E+02	6.2896E+02	6.0122E+02
	Avg	6.4819E+02	6.1194E+02	6.1264E+02	6.4854E+02	6.0472E+02	6.4103E+02	6.4104E+02	6.0340E+02
	Std	9.6799E+00	5.1610E+00	5.4919E+00	1.0872E+01	2.9547E+00	8.7933E+00	8.3689E+00	1.7467E+00
	Rank	7	3	4	8	2	5	6	1
F6	Best	9.4885E+02	8.6284E+02	8.8462E+02	9.3392E+02	8.0285E+02	8.7394E+02	8.7878E+02	7.4415E+02
	Avg	1.1363E+03	9.2356E+02	9.2254E+02	1.0385E+03	8.6304E+02	9.6458E+02	9.7754E+02	7.6706E+02
	Std	1.0150E+02	3.9180E+01	2.8961E+01	7.8808E+01	2.6589E+01	6.7735E+01	7.4369E+01	1.6126E+01
	Rank	8	4	3	7	2	5	6	1
F7	Best	9.6946E+02	9.3324E+02	9.3928E+02	9.0278E+02	8.2132E+02	9.0745E+02	8.7263E+02	8.1094E+02
	Avg	1.0281E+03	9.8026E+02	9.8227E+02	9.6252E+02	8.9738E+02	9.5535E+02	9.3193E+02	8.2633E+02
	Std	3.4838E+01	2.8684E+01	2.3951E+01	3.5315E+01	3.5235E+01	3.2893E+01	3.4829E+01	1.0768E+01
	Rank	8	6	7	5	2	4	3	1
F8	Best	3.7868E+03	9.5982E+02	9.7128E+02	3.1158E+03	9.1764E+02	2.2491E+03	2.3138E+03	9.0235E+02
	Avg	6.6297E+03	1.4558E+03	1.2863E+03	5.1900E+03	1.1957E+03	3.6962E+03	3.6514E+03	1.0753E+03
	Std	1.7678E+03	5.5181E+02	2.7043E+02	1.8027E+03	2.9774E+02	1.2067E+03	9.4916E+02	2.6070E+02
	Rank	8	4	3	7	2	6	5	1
F9	Best	6.5418E+03	6.3440E+03	7.0759E+03	5.4871E+03	4.8488E+03	4.0567E+03	4.0790E+03	4.0675E+03
	Avg	7.4701E+03	7.4236E+03	8.0889E+03	6.7432E+03	7.2895E+03	4.8989E+03	5.1966E+03	5.0357E+03
	Std	5.0869E+02	6.1233E+02	5.5973E+02	7.8718E+02	9.3952E+02	5.1170E+02	5.9389E+02	5.9698E+02
	Rank	7	6	8	4	5	1	3	2
F10	Best	1.4005E+03	1.1892E+03	1.2037E+03	1.2586E+03	1.1220E+03	1.1667E+03	1.1438E+03	1.1318E+03
	Avg	2.4572E+03	1.2613E+03	1.2827E+03	1.4142E+03	1.1802E+03	1.2338E+03	1.2346E+03	1.1611E+03
	Std	6.0466E+02	5.5683E+01	1.0807E+02	1.0508E+02	3.7790E+01	4.8871E+01	6.0679E+01	2.1430E+01
	Rank	8	5	6	7	2	3	4	1
F11	Best	4.0632E+07	1.0030E+05	3.5878E+05	6.5983E+06	5.0402E+03	1.6490E+03	1.7116E+03	1.6653E+03
	Avg	3.7988E+08	3.7370E+07	7.7752E+06	2.7562E+07	2.8178E+05	2.5964E+03	4.9496E+03	2.3615E+03
	Std	2.9301E+08	9.4521E+07	1.5554E+07	3.0189E+07	4.7958E+05	7.3408E+02	6.0188E+03	4.0135E+02
	Rank	8	7	5	6	4	2	3	1
F12	Best	1.5773E+05	1.5525E+04	2.4240E+04	3.5489E+04	3.6691E+03	2.1581E+03	2.0606E+03	1.5151E+03
	Avg	3.9887E+06	4.5509E+04	5.4911E+04	1.3226E+05	1.1289E+04	3.6466E+03	3.3834E+03	2.2556E+03
	Std	7.3126E+06	2.5748E+04	2.0264E+04	9.5507E+04	7.9113E+03	1.1719E+03	7.8453E+02	6.0513E+02
	Rank	8	5	6	7	4	3	2	1
F13	Best	1.9405E+04	1.5170E+03	1.5180E+03	1.9972E+03	1.4547E+03	1.5167E+03	1.5089E+03	1.4290E+03
	Avg	2.0366E+05	1.5933E+03	1.5740E+03	1.7909E+04	1.4818E+03	1.6571E+03	1.6318E+03	1.4809E+03
	Std	1.8579E+05	1.3154E+02	3.4393E+01	3.3564E+04	2.4028E+01	8.9175E+01	6.7059E+01	3.7896E+01
	Rank	8	4	3	7	2	6	5	1
F14	Best	1.4059E+04	2.1148E+03	2.1998E+03	7.5684E+03	1.7285E+03	1.5716E+03	1.5804E+03	1.5598E+03
	Avg	4.2983E+05	5.0697E+03	8.8400E+03	6.8526E+04	2.1135E+03	1.8774E+03	1.7819E+03	1.6608E+03
	Std	1.2268E+06	2.9651E+03	8.6126E+03	5.7556E+04	3.5867E+02	3.6014E+02	1.5244E+02	4.9470E+01
	Rank	8	5	6	7	4	3	2	1
F15	Best	2.6898E+03	2.3336E+03	2.4629E+03	2.5848E+03	1.7160E+03	1.9750E+03	2.3100E+03	1.6027E+03
	Avg	3.1720E+03	2.7933E+03	2.9451E+03	3.0091E+03	2.0782E+03	2.5766E+03	2.7050E+03	1.8519E+03
	Std	2.9296E+02	2.7696E+02	2.8682E+02	2.7534E+02	2.7633E+02	2.9623E+02	2.9820E+02	2.1228E+02
	Rank	8	5	6	7	2	3	4	1
F16	Best	2.1910E+03	1.8304E+03	1.8650E+03	1.9721E+03	1.7834E+03	1.8629E+03	1.9335E+03	1.7361E+03
	Avg	2.4576E+03	2.0697E+03	2.1045E+03	2.2878E+03	1.8700E+03	2.1524E+03	2.2462E+03	1.8085E+03
	Std	2.2819E+02	1.5677E+02	1.8707E+02	2.3365E+02	1.1447E+02	1.8401E+02	2.3419E+02	7.7009E+01
	Rank	8	3	4	7	2	5	6	1
F17	Best	8.0686E+04	2.2224E+03	2.3376E+03	5.7372E+04	1.9583E+03	1.8886E+03	1.8660E+03	1.8689E+03
	Avg	1.2248E+06	5.6642E+03	5.4309E+03	1.3309E+05	2.1134E+03	2.0484E+03	7.1229E+03	1.9368E+03
	Std	1.0334E+06	5.2587E+03	3.2327E+03	7.6701E+04	1.4917E+02	1.3175E+02	9.2773E+03	5.3946E+01
	Rank	8	5	4	7	3	2	6	1
F18	Best	2.3623E+04	2.0776E+03	2.2630E+03	2.0819E+04	1.9433E+03	2.0542E+03	2.1738E+03	1.9224E+03
	Avg	9.8455E+06	2.7398E+03	3.7883E+03	2.8909E+05	2.0020E+03	2.2486E+03	6.0659E+03	1.9971E+03
	Std	2.9716E+07	1.2678E+03	1.7637E+03	3.9419E+05	4.2065E+01	1.7149E+02	9.5810E+03	5.4650E+01
	Rank	8	4	5	7	2	3	6	1
F19	Best	2.3612E+03	2.2060E+03	2.3893E+03	2.3155E+03	2.1107E+03	2.2834E+03	2.2706E+03	2.0256E+03
	Avg	2.6477E+03	2.5218E+03	2.6138E+03	2.5671E+03	2.2396E+03	2.5382E+03	2.5171E+03	2.0490E+03
	Std	1.6299E+02	2.0224E+02	1.2031E+02	1.6249E+02	1.5026E+02	1.5133E+02	1.7044E+02	1.6675E+01
	Rank	8	4	7	6	2	5	3	1
F20	Best	2.4608E+03	2.3833E+03	2.3928E+03	2.4110E+03	2.3267E+03	2.3459E+03	2.3816E+03	2.3131E+03
	Avg	2.5363E+03	2.4466E+03	2.4557E+03	2.4627E+03	2.3686E+03	2.4229E+03	2.4155E+03	2.3354E+03
	Std	4.4288E+01	3.4518E+01	2.8925E+01	4.1230E+01	3.2884E+01	4.0401E+01	3.4676E+01	2.8757E+01
	Rank	8	5	6	7	2	4	3	1
F21	Best	2.9319E+03	2.4166E+03	2.4519E+03	2.4245E+03	2.3151E+03	2.3000E+03	2.3000E+03	2.3000E+03
	Avg	5.2722E+03	3.8404E+03	3.7365E+03	4.0317E+03	2.4357E+03	4.2838E+03	3.3425E+03	2.4892E+03
	Std	2.2308E+03	1.9855E+03	1.4409E+03	2.3439E+03	1.7241E+02	2.2360E+03	1.8245E+03	7.3176E+02
	Rank	8	5	4	6	1	7	3	2
F22	Best	2.8757E+03	2.7724E+03	2.7939E+03	2.8340E+03	2.6626E+03	2.7538E+03	2.7725E+03	2.6611E+03
	Avg	2.9241E+03	2.8348E+03	2.8377E+03	2.8858E+03	2.7156E+03	2.8427E+03	2.8402E+03	2.6858E+03
	Std	5.1046E+01	4.9513E+01	2.8745E+01	4.1342E+01	2.7234E+01	6.6398E+01	4.8614E+01	2.3121E+01
	Rank	8	3	4	7	2	6	5	1
F23	Best	2.9973E+03	2.9517E+03	2.9748E+03	2.9414E+03	2.8469E+03	2.8932E+03	2.9091E+03	2.8326E+03
	Avg	3.0785E+03	3.0189E+03	3.0171E+03	3.0169E+03	2.8805E+03	2.9699E+03	2.9674E+03	2.8743E+03
	Std	4.9058E+01	7.3977E+01	4.6858E+01	5.1718E+01	3.3433E+01	5.0838E+01	5.3107E+01	3.4835E+01
	Rank	8	7	6	5	2	4	3	1
F24	Best	3.1117E+03	2.8935E+03	2.9100E+03	2.9610E+03	2.8858E+03	2.8751E+03	2.8752E+03	2.8755E+03
	Avg	3.3040E+03	2.9302E+03	2.9345E+03	3.0151E+03	2.9057E+03	2.8774E+03	2.8771E+03	2.8831E+03
	Std	1.6689E+02	4.1794E+01	4.7959E+01	3.8322E+01	1.7430E+01	2.5243E+00	3.2926E+00	1.6523E+01
	Rank	8	5	6	7	4	2	1	3
F25	Best	5.3487E+03	3.1305E+03	3.3116E+03	5.3269E+03	3.2700E+03	2.9000E+03	4.6602E+03	3.5533E+03
	Avg	6.6579E+03	4.8493E+03	5.2204E+03	5.8677E+03	4.1412E+03	4.9630E+03	5.3942E+03	3.9352E+03
	Std	6.6267E+02	1.0060E+03	7.6924E+02	5.0783E+02	4.0722E+02	1.4750E+03	5.1564E+02	2.2284E+02
	Rank	8	3	5	7	2	4	6	1
F26	Best	3.2547E+03	3.2176E+03	3.2233E+03	3.2314E+03	3.2085E+03	3.1688E+03	3.1636E+03	3.1264E+03
	Avg	3.3182E+03	3.2536E+03	3.2488E+03	3.2872E+03	3.2505E+03	3.1979E+03	3.1936E+03	3.1792E+03
	Std	3.7827E+01	3.0498E+01	2.5436E+01	4.4111E+01	3.1063E+01	8.0688E+00	1.3284E+01	1.8144E+01
	Rank	8	6	4	7	5	3	2	1
F27	Best	3.4442E+03	3.2703E+03	3.2752E+03	3.3682E+03	3.2251E+03	3.1000E+03	3.1000E+03	3.1000E+03
	Avg	4.2056E+03	3.3369E+03	3.3592E+03	3.5233E+03	3.2808E+03	3.1507E+03	3.1227E+03	3.1320E+03
	Std	6.9418E+02	6.6577E+01	8.3898E+01	1.1054E+02	3.6798E+01	7.8381E+01	4.7573E+01	5.6754E+01
	Rank	8	5	6	7	4	3	1	2
F28	Best	4.1256E+03	3.6850E+03	3.6488E+03	3.7986E+03	3.4876E+03	3.4398E+03	3.5460E+03	3.3836E+03
	Avg	4.5468E+03	3.9772E+03	4.0779E+03	4.4144E+03	3.6942E+03	4.0674E+03	4.1826E+03	3.5263E+03
	Std	2.2051E+02	1.7627E+02	3.8359E+02	3.5171E+02	1.6609E+02	2.9184E+02	3.4894E+02	9.7478E+01
	Rank	8	3	5	7	2	4	6	1
F29	Best	1.5259E+06	1.4114E+04	3.0712E+04	2.2365E+05	8.9715E+03	3.3970E+03	3.4184E+03	3.4105E+03
	Avg	1.5448E+07	7.0342E+04	1.3357E+05	2.0004E+06	2.1761E+04	4.0381E+03	4.3288E+03	3.7782E+03
	Std	1.9800E+07	5.8297E+04	1.9060E+05	1.7810E+06	1.1941E+04	1.3977E+03	1.6741E+03	3.7754E+02
	Rank	8	5	6	7	4	2	3	1

**Table A11:** Experiments comparing EECO with different strategies (50D).

No.	Index	ECO	ECO-R	ECO-P	ECO-T	ECO-RP	ECO-RT	ECO-PT	EECO
F1	Best	1.4874E+10	1.7123E+09	1.6333E+09	3.8342E+09	4.6351E+08	1.0000E+02	1.0000E+02	1.0000E+02
	Avg	2.9614E+10	9.8341E+09	8.8323E+09	8.3151E+09	1.1693E+09	1.0000E+02	1.0000E+02	1.0000E+02
	Std	1.5809E+10	9.7645E+09	9.6953E+09	3.0841E+09	7.0789E+08	4.3406E-06	9.5542E-06	7.7591E-08
	Rank	8	7	6	5	4	2	3	1
F2	Best	8.6958E+04	1.3413E+04	1.7793E+04	4.0427E+04	1.1476E+04	3.0000E+02	3.0006E+02	3.0000E+02
	Avg	1.1643E+05	3.0235E+04	2.7543E+04	5.6692E+04	1.8150E+04	3.0000E+02	3.1669E+02	3.0000E+02
	Std	2.9299E+04	1.0143E+04	1.0143E+04	1.1521E+04	5.1606E+03	2.1733E-05	4.3195E+01	1.0301E-08
	Rank	8	6	5	7	4	2	3	1
F3	Best	2.7984E+03	8.2303E+02	7.2959E+02	9.1188E+02	6.0870E+02	4.0000E+02	4.0000E+02	4.0000E+02
	Avg	4.8313E+03	1.2752E+03	1.0869E+03	1.5008E+03	7.0146E+02	4.0100E+02	4.0335E+02	4.0199E+02
	Std	1.7254E+03	7.0589E+02	6.0638E+02	3.6545E+02	1.0995E+02	1.8454E+00	5.6149E+00	2.1309E+00
	Rank	8	6	5	7	4	1	3	2
F4	Best	8.1635E+02	8.2108E+02	8.2996E+02	7.9781E+02	7.3342E+02	6.9402E+02	7.0297E+02	5.4676E+02
	Avg	9.6664E+02	8.5429E+02	8.7278E+02	8.8026E+02	7.8505E+02	7.9612E+02	7.8916E+02	5.7935E+02
	Std	7.3984E+01	2.7265E+01	3.6789E+01	5.0990E+01	3.0231E+01	7.3018E+01	5.0677E+01	2.5971E+01
	Rank	8	5	6	7	2	4	3	1
F5	Best	6.5941E+02	6.2107E+02	6.1759E+02	6.5943E+02	6.1138E+02	6.4507E+02	6.4595E+02	6.0232E+02
	Avg	6.7160E+02	6.2904E+02	6.2871E+02	6.6862E+02	6.1788E+02	6.5061E+02	6.5496E+02	6.0987E+02
	Std	8.1593E+00	5.2394E+00	6.4558E+00	5.7197E+00	5.4146E+00	5.0228E+00	4.4183E+00	3.7701E+00
	Rank	8	4	3	7	2	5	6	1
F6	Best	1.3615E+03	1.0351E+03	1.0313E+03	1.3257E+03	9.8508E+02	1.1267E+03	1.1796E+03	8.0582E+02
	Avg	1.6051E+03	1.1570E+03	1.1891E+03	1.4802E+03	1.0949E+03	1.2739E+03	1.3760E+03	8.7598E+02
	Std	1.5625E+02	7.3118E+01	1.0628E+02	9.2225E+01	7.6483E+01	8.4439E+01	1.1379E+02	7.8800E+01
	Rank	8	3	4	7	2	5	6	1
F7	Best	1.2552E+03	1.1264E+03	1.0823E+03	1.0244E+03	1.0105E+03	1.0209E+03	9.8705E+02	8.3582E+02
	Avg	1.3222E+03	1.1789E+03	1.1675E+03	1.1122E+03	1.0671E+03	1.1046E+03	1.0423E+03	8.6753E+02
	Std	5.8474E+01	2.9432E+01	4.1376E+01	5.7318E+01	4.6133E+01	6.1297E+01	3.5181E+01	2.2648E+01
	Rank	8	7	6	5	3	4	2	1
F8	Best	1.6640E+04	1.7049E+03	2.5857E+03	9.2717E+03	2.5501E+03	7.5756E+03	5.8310E+03	1.0153E+03
	Avg	2.1625E+04	4.8220E+03	6.0812E+03	1.5193E+04	3.5753E+03	1.0058E+04	8.4329E+03	1.6855E+03
	Std	6.6129E+03	4.2760E+03	6.3383E+03	6.0349E+03	1.1918E+03	2.1674E+03	1.9709E+03	4.7646E+02
	Rank	8	3	4	7	2	6	5	1
F9	Best	1.0599E+04	1.1526E+04	1.2637E+04	1.0489E+04	1.0624E+04	6.2082E+03	6.8800E+03	6.4855E+03
	Avg	1.2343E+04	1.3299E+04	1.3972E+04	1.1267E+04	1.2558E+04	7.5381E+03	8.2826E+03	7.4470E+03
	Std	1.2418E+03	1.3735E+03	7.6840E+02	6.5535E+02	1.2915E+03	6.5971E+02	1.0427E+03	8.5769E+02
	Rank	5	7	8	4	6	2	3	1
F10	Best	2.6975E+03	1.3878E+03	1.3264E+03	1.8208E+03	1.2738E+03	1.2572E+03	1.2313E+03	1.1786E+03
	Avg	7.5659E+03	1.6097E+03	1.6201E+03	2.2931E+03	1.3425E+03	1.3175E+03	1.3173E+03	1.2298E+03
	Std	4.5208E+03	2.3855E+02	3.9884E+02	3.8392E+02	5.9890E+01	4.2936E+01	6.7230E+01	3.4289E+01
	Rank	8	5	6	7	4	3	2	1
F11	Best	1.9510E+09	8.3336E+06	3.7849E+06	1.5806E+08	1.7923E+06	2.8989E+03	4.6668E+03	2.3988E+03
	Avg	4.4511E+09	5.9950E+07	7.7960E+07	3.3635E+08	7.4344E+06	5.8561E+03	2.5439E+04	3.8381E+03
	Std	3.5347E+09	6.0746E+07	1.3398E+08	1.9894E+08	4.9164E+06	2.2055E+03	2.0190E+04	6.6394E+02
	Rank	8	5	6	7	4	2	3	1
F12	Best	4.7945E+07	4.7905E+04	4.9773E+04	7.3866E+04	1.0358E+04	3.0276E+03	4.9996E+03	2.5129E+03
	Avg	4.2634E+08	2.1170E+05	1.1106E+06	5.7909E+05	1.7891E+04	3.3130E+03	8.1830E+03	3.0418E+03
	Std	4.9571E+08	2.2248E+05	1.7696E+06	4.6714E+05	7.6117E+03	3.6544E+02	3.1187E+03	4.0548E+02
	Rank	8	5	7	6	4	2	3	1
F13	Best	1.1669E+05	1.6854E+03	1.7699E+03	2.2665E+03	1.4874E+03	1.6111E+03	1.9524E+03	1.4956E+03
	Avg	8.4291E+05	2.7260E+03	3.7072E+03	8.1511E+04	1.5945E+03	1.8441E+03	4.6066E+03	1.5629E+03
	Std	8.8990E+05	2.6112E+03	4.6656E+03	6.7338E+04	6.6744E+01	1.5945E+02	4.7739E+03	5.6328E+01
	Rank	8	4	5	7	2	3	6	1
F14	Best	9.5511E+04	5.6021E+03	1.0829E+04	2.5769E+04	2.3075E+03	1.6696E+03	1.8519E+03	1.6450E+03
	Avg	4.1685E+06	1.7395E+04	3.4837E+04	1.1045E+05	2.7369E+03	2.2358E+03	4.2873E+03	1.7827E+03
	Std	4.8105E+06	6.2062E+03	2.7374E+04	1.6313E+05	3.1011E+02	7.2906E+02	3.5472E+03	6.5285E+01
	Rank	8	5	6	7	3	2	4	1
F15	Best	3.2636E+03	2.8973E+03	3.0888E+03	3.3822E+03	2.1358E+03	3.0117E+03	2.7560E+03	1.7416E+03
	Avg	4.3534E+03	3.5177E+03	3.7788E+03	4.2558E+03	2.5847E+03	3.5861E+03	3.6697E+03	1.9631E+03
	Std	7.7063E+02	6.2110E+02	4.8045E+02	4.4748E+02	3.0049E+02	3.8581E+02	4.1430E+02	1.2251E+02
	Rank	8	3	6	7	2	4	5	1
F16	Best	3.1359E+03	2.7466E+03	3.0389E+03	2.8693E+03	2.1334E+03	2.8187E+03	2.8073E+03	1.9354E+03
	Avg	3.8276E+03	3.3290E+03	3.4925E+03	3.6193E+03	2.6443E+03	3.5093E+03	3.5675E+03	2.0508E+03
	Std	4.5072E+02	4.9808E+02	2.8293E+02	4.2762E+02	3.9745E+02	5.0125E+02	4.0066E+02	8.9304E+01
	Rank	8	3	4	7	2	5	6	1
F17	Best	3.5091E+06	1.0936E+04	2.1723E+04	1.1425E+05	2.1375E+03	1.9784E+03	2.3083E+03	1.9169E+03
	Avg	1.1216E+07	2.4590E+04	3.8602E+04	1.3543E+06	6.0302E+03	2.3890E+03	2.1475E+04	2.0283E+03
	Std	1.3490E+07	1.1247E+04	1.6974E+04	1.1766E+06	5.8596E+03	4.6908E+02	2.4402E+04	8.3522E+01
	Rank	8	5	6	7	3	2	4	1
F18	Best	4.1986E+05	8.0106E+03	9.3311E+03	2.2059E+04	2.0721E+03	2.2270E+03	5.2715E+03	2.0234E+03
	Avg	7.3520E+06	3.1345E+04	2.4770E+04	2.8562E+05	2.6649E+03	2.9938E+03	1.4985E+04	2.0971E+03
	Std	1.2712E+07	3.2994E+04	1.4089E+04	3.8331E+05	1.2428E+03	1.2753E+03	6.4999E+03	5.8685E+01
	Rank	8	6	5	7	2	3	4	1
F19	Best	3.3478E+03	2.8489E+03	2.8556E+03	2.9419E+03	2.3714E+03	2.7335E+03	2.8232E+03	2.0586E+03
	Avg	3.5609E+03	3.5067E+03	3.4662E+03	3.3297E+03	3.0750E+03	3.2558E+03	3.2011E+03	2.2240E+03
	Std	2.4162E+02	4.4595E+02	2.8919E+02	1.9881E+02	3.7567E+02	2.3997E+02	1.8083E+02	3.0035E+02
	Rank	8	7	6	5	2	4	3	1
F20	Best	2.6066E+03	2.5936E+03	2.6141E+03	2.5781E+03	2.4538E+03	2.4566E+03	2.5187E+03	2.3290E+03
	Avg	2.7280E+03	2.6639E+03	2.6577E+03	2.6625E+03	2.5206E+03	2.5676E+03	2.5910E+03	2.3535E+03
	Std	9.3109E+01	4.2170E+01	3.4769E+01	6.3651E+01	4.0789E+01	7.1380E+01	5.6310E+01	1.3533E+01
	Rank	8	7	5	6	2	3	4	1
F21	Best	1.3298E+04	1.2450E+04	1.3912E+04	1.0328E+04	1.1350E+04	8.5844E+03	7.6036E+03	5.0448E+03
	Avg	1.4634E+04	1.4875E+04	1.5332E+04	1.2361E+04	1.3203E+04	9.6616E+03	9.4985E+03	7.6899E+03
	Std	9.9210E+02	1.1235E+03	7.2527E+02	1.1897E+03	1.5122E+03	6.9669E+02	1.0203E+03	1.7365E+03
	Rank	6	7	8	4	5	3	2	1
F22	Best	3.3141E+03	2.9880E+03	3.0555E+03	3.0071E+03	2.8528E+03	3.0951E+03	2.9323E+03	2.8043E+03
	Avg	3.3923E+03	3.0999E+03	3.1313E+03	3.1753E+03	2.9410E+03	3.1631E+03	3.0839E+03	2.8293E+03
	Std	7.9087E+01	6.0151E+01	5.4227E+01	1.3935E+02	7.0170E+01	7.7899E+01	1.3450E+02	2.4471E+01
	Rank	8	4	5	7	2	6	3	1
F23	Best	3.3913E+03	3.2301E+03	3.1913E+03	3.1977E+03	3.0424E+03	3.1700E+03	3.1203E+03	2.9313E+03
	Avg	3.4727E+03	3.3223E+03	3.3158E+03	3.3363E+03	3.0782E+03	3.2764E+03	3.2253E+03	2.9771E+03
	Std	5.6544E+01	7.0301E+01	7.3889E+01	8.9988E+01	3.9342E+01	7.1199E+01	8.7769E+01	3.1910E+01
	Rank	8	6	5	7	2	4	3	1
F24	Best	4.0832E+03	3.1133E+03	3.1674E+03	3.4361E+03	3.0852E+03	2.9312E+03	2.9312E+03	2.9312E+03
	Avg	5.6884E+03	3.2933E+03	3.4664E+03	3.9312E+03	3.2246E+03	2.9484E+03	2.9510E+03	2.9704E+03
	Std	1.1749E+03	1.3212E+02	3.8706E+02	3.2416E+02	1.1465E+02	2.8327E+01	2.1151E+01	5.5206E+01
	Rank	8	5	6	7	4	1	2	3
F25	Best	9.4028E+03	4.6049E+03	4.9863E+03	8.1750E+03	4.6817E+03	2.9000E+03	6.8057E+03	2.9000E+03
	Avg	1.0666E+04	9.3013E+03	9.1580E+03	9.5167E+03	5.4805E+03	6.9772E+03	8.6172E+03	5.1016E+03
	Std	1.0610E+03	2.8115E+03	2.6140E+03	1.0717E+03	6.6492E+02	2.6369E+03	1.0896E+03	1.6157E+03
	Rank	8	6	5	7	2	3	4	1
F26	Best	3.7640E+03	3.5132E+03	3.5289E+03	3.5667E+03	3.4597E+03	3.2000E+03	3.2000E+03	3.2000E+03
	Avg	3.8678E+03	3.7966E+03	3.6723E+03	3.7331E+03	3.6828E+03	3.2000E+03	3.2877E+03	3.2832E+03
	Std	1.0946E+02	1.9632E+02	1.2993E+02	9.3677E+01	1.7884E+02	2.3037E-04	1.5233E+02	1.1593E+02
	Rank	8	7	4	6	5	1	3	2
F27	Best	5.7906E+03	3.5320E+03	3.5273E+03	3.9688E+03	3.4223E+03	3.3000E+03	3.3000E+03	3.3000E+03
	Avg	6.9434E+03	3.9976E+03	4.0915E+03	5.2151E+03	3.7022E+03	3.3000E+03	3.3000E+03	3.3000E+03
	Std	1.0679E+03	6.6630E+02	6.2240E+02	1.7850E+03	3.2882E+02	2.8917E-04	4.3785E-04	2.3095E-04
	Rank	8	5	6	7	4	2	1	3
F28	Best	4.8206E+03	4.0169E+03	4.6036E+03	4.8315E+03	3.7132E+03	4.0221E+03	3.8569E+03	3.2792E+03
	Avg	5.9201E+03	4.5784E+03	4.9916E+03	5.6036E+03	3.9604E+03	4.6594E+03	4.6112E+03	3.5197E+03
	Std	9.7895E+02	3.9747E+02	3.2822E+02	5.3689E+02	2.4007E+02	3.6165E+02	4.3303E+02	1.7277E+02
	Rank	8	3	6	7	2	5	4	1
F29	Best	7.6148E+07	5.9299E+06	5.7643E+06	9.7692E+06	4.1470E+06	3.5642E+03	4.2650E+03	3.7023E+03
	Avg	1.1956E+08	1.6112E+07	1.6586E+07	3.3596E+07	6.5852E+06	4.4847E+03	7.3233E+03	4.9940E+03
	Std	4.7460E+07	9.4964E+06	7.7069E+06	1.6651E+07	4.3394E+06	1.1864E+03	2.5869E+03	8.4520E+02
	Rank	8	5	6	7	4	1	3	2

**Table A12:** Experiments comparing EECO with different strategies (100D).

No.	Index	ECO	ECO-R	ECO-P	ECO-T	ECO-RP	ECO-RT	ECO-PT	EECO
F1	Best	1.0516E+11	3.0095E+10	3.1271E+10	4.1183E+10	1.5766E+10	1.0000E+02	1.0000E+02	1.0000E+02
	Avg	1.1103E+11	5.2045E+10	5.0924E+10	4.6461E+10	2.1746E+10	1.0003E+02	1.0001E+02	1.0000E+02
	Std	4.9348E+09	2.0849E+10	1.8951E+10	5.7708E+09	4.8158E+09	6.0942E-02	2.4349E-02	6.4717E-06
	Rank	8	7	6	5	4	3	2	1
F2	Best	2.5917E+05	7.4127E+04	8.5360E+04	1.7383E+05	6.1414E+04	3.0000E+02	3.1346E+03	3.0000E+02
	Avg	2.8050E+05	1.0376E+05	1.1663E+05	1.8919E+05	7.1423E+04	3.0007E+02	5.9298E+03	3.0000E+02
	Std	2.5113E+04	2.6599E+04	3.1513E+04	2.1177E+04	7.5450E+03	7.9467E-02	3.6469E+03	2.1971E-06
	Rank	8	5	6	7	4	2	3	1
F3	Best	1.1491E+04	2.1286E+03	3.0027E+03	2.8217E+03	1.9486E+03	4.0002E+02	5.0524E+02	4.0000E+02
	Avg	1.6719E+04	3.2376E+03	5.2968E+03	5.7124E+03	3.0397E+03	4.0201E+02	5.3509E+02	4.0100E+02
	Std	8.1866E+03	7.4036E+02	4.0948E+03	2.8925E+03	8.4657E+02	2.2839E+00	2.6686E+01	1.9933E+00
	Rank	8	5	6	7	4	2	3	1
F4	Best	1.5748E+03	1.2840E+03	1.3118E+03	1.3241E+03	1.2387E+03	1.1666E+03	1.1039E+03	7.2685E+02
	Avg	1.6415E+03	1.3965E+03	1.4168E+03	1.4724E+03	1.3059E+03	1.2034E+03	1.2412E+03	7.7287E+02
	Std	4.5651E+01	9.8467E+01	7.1990E+01	1.3526E+02	6.6340E+01	4.4628E+01	1.2893E+02	3.9702E+01
	Rank	8	5	6	7	4	2	3	1
F5	Best	6.7534E+02	6.3345E+02	6.4480E+02	6.7409E+02	6.2185E+02	6.5284E+02	6.5792E+02	6.1417E+02
	Avg	6.7727E+02	6.4381E+02	6.5320E+02	6.8050E+02	6.2960E+02	6.5748E+02	6.6026E+02	6.1620E+02
	Std	1.9568E+00	8.6019E+00	7.1155E+00	4.7358E+00	6.5724E+00	5.4894E+00	2.7109E+00	1.4088E+00
	Rank	7	3	4	8	2	5	6	1
F6	Best	3.1609E+03	2.0400E+03	1.8295E+03	2.5417E+03	1.7047E+03	2.4412E+03	2.2132E+03	1.1324E+03
	Avg	3.2687E+03	2.0882E+03	2.0493E+03	2.9158E+03	1.9189E+03	2.6823E+03	2.5437E+03	1.4027E+03
	Std	1.3907E+02	3.9495E+01	1.4911E+02	2.8265E+02	1.6912E+02	2.1764E+02	2.7580E+02	2.5504E+02
	Rank	8	4	3	7	2	6	5	1
F7	Best	1.8844E+03	1.6015E+03	1.6520E+03	1.6237E+03	1.3535E+03	1.5184E+03	1.3850E+03	9.4725E+02
	Avg	2.0902E+03	1.6806E+03	1.7266E+03	1.8038E+03	1.5402E+03	1.5522E+03	1.5417E+03	1.0709E+03
	Std	1.5760E+02	5.9198E+01	8.7412E+01	1.5709E+02	1.5571E+02	4.2327E+01	1.5035E+02	9.9907E+01
	Rank	8	5	6	7	2	4	3	1
F8	Best	4.8102E+04	1.4406E+04	1.4183E+04	2.6799E+04	1.0715E+04	1.6519E+04	1.5908E+04	4.3083E+03
	Avg	5.6594E+04	1.8187E+04	1.8627E+04	3.8813E+04	1.3069E+04	1.9516E+04	1.8307E+04	5.1535E+03
	Std	6.9367E+03	4.5297E+03	3.1164E+03	9.8444E+03	3.2253E+03	2.0485E+03	3.0703E+03	7.2934E+02
	Rank	8	3	5	7	2	6	4	1
F9	Best	2.5800E+04	2.7285E+04	2.7926E+04	2.0376E+04	1.9588E+04	1.5219E+04	1.5109E+04	1.4432E+04
	Avg	2.7131E+04	2.9396E+04	2.9808E+04	2.2490E+04	2.5476E+04	1.6132E+04	1.5729E+04	1.6239E+04
	Std	1.5785E+03	1.4523E+03	1.9246E+03	1.4374E+03	3.9830E+03	8.9264E+02	5.0605E+02	1.2876E+03
	Rank	6	7	8	4	5	2	1	3
F10	Best	5.0265E+04	3.3547E+03	4.5879E+03	1.1749E+04	3.2895E+03	2.0611E+03	2.0025E+03	1.9029E+03
	Avg	8.1274E+04	4.7507E+03	6.6031E+03	1.9530E+04	4.0586E+03	2.3093E+03	2.4283E+03	2.1138E+03
	Std	3.9368E+04	1.2202E+03	1.6881E+03	6.0838E+03	6.9905E+02	3.5187E+02	3.9237E+02	1.9880E+02
	Rank	8	5	6	7	4	2	3	1
F11	Best	1.9040E+10	6.2793E+08	6.2023E+08	1.5924E+09	1.5848E+08	6.8913E+03	7.9901E+04	5.6050E+03
	Avg	2.7304E+10	4.2740E+09	1.0329E+09	3.0342E+09	2.7863E+08	1.3951E+04	4.8239E+05	6.2549E+03
	Std	6.5837E+09	7.0913E+09	3.7283E+08	2.3360E+09	8.0207E+07	1.0682E+04	4.3477E+05	6.7961E+02
	Rank	8	7	5	6	4	2	3	1
F12	Best	4.4658E+08	9.9737E+04	5.8450E+05	2.9253E+06	3.3295E+04	5.2264E+03	8.6177E+03	4.4995E+03
	Avg	1.7600E+09	6.8901E+05	5.0099E+06	1.5838E+07	4.9695E+04	5.7215E+03	1.2482E+04	5.4561E+03
	Std	1.0977E+09	7.3069E+05	3.7033E+06	9.2656E+06	1.6833E+04	3.3285E+02	6.7082E+03	7.5747E+02
	Rank	8	5	6	7	4	2	3	1
F13	Best	2.4685E+06	4.3975E+03	1.0048E+04	7.5838E+05	1.9051E+03	1.8910E+03	8.0619E+04	1.6668E+03
	Avg	9.9605E+06	4.3691E+04	2.7520E+04	1.4399E+06	1.9514E+03	1.9718E+03	1.2106E+05	1.7312E+03
	Std	1.1653E+07	4.1511E+04	1.8330E+04	5.1181E+05	4.1764E+01	8.2902E+01	5.6113E+04	5.7043E+01
	Rank	8	5	4	7	2	3	6	1
F14	Best	8.5045E+07	2.3734E+04	7.7999E+04	2.3842E+05	4.3368E+03	1.7363E+03	3.6125E+03	1.7283E+03
	Avg	1.4398E+08	1.3860E+05	6.3094E+05	5.9576E+05	1.4861E+04	2.1163E+03	8.6434E+03	1.8265E+03
	Std	6.2725E+07	1.5569E+05	1.0637E+06	5.3556E+05	7.5104E+03	3.6610E+02	6.6372E+03	1.2393E+02
	Rank	8	5	7	6	4	2	3	1
F15	Best	8.9502E+03	6.5134E+03	8.8078E+03	7.3823E+03	5.8130E+03	5.1252E+03	5.4745E+03	2.3725E+03
	Avg	9.3818E+03	8.2152E+03	8.9009E+03	7.8220E+03	6.2840E+03	5.4526E+03	6.2043E+03	2.6452E+03
	Std	4.4861E+02	1.1734E+03	1.5465E+02	3.5841E+02	5.7924E+02	3.5077E+02	5.1416E+02	2.7448E+02
	Rank	8	6	7	5	4	2	3	1
F16	Best	6.3541E+03	4.6568E+03	5.2042E+03	4.8201E+03	5.0372E+03	5.3604E+03	4.8585E+03	2.4919E+03
	Avg	8.3109E+03	5.9415E+03	6.1256E+03	5.9874E+03	5.3528E+03	6.2674E+03	5.8825E+03	2.6102E+03
	Std	2.3441E+03	1.2160E+03	6.7674E+02	1.0010E+03	3.9766E+02	7.1808E+02	9.3651E+02	1.0639E+02
	Rank	8	4	6	5	2	7	3	1
F17	Best	1.8859E+06	4.3167E+04	1.0863E+05	7.8303E+05	3.5819E+03	2.0733E+03	5.5668E+04	1.9816E+03
	Avg	7.1368E+06	8.0853E+04	1.4492E+05	1.5538E+06	9.7584E+03	2.3476E+03	8.1009E+04	2.0734E+03
	Std	4.2842E+06	3.9796E+04	3.8905E+04	1.3845E+06	6.9019E+03	3.4047E+02	1.8989E+04	8.7551E+01
	Rank	8	4	6	7	3	2	5	1
F18	Best	1.6028E+08	4.4227E+05	9.9918E+05	2.3379E+06	4.9476E+03	2.4134E+03	3.7153E+03	2.1091E+03
	Avg	4.5686E+08	2.0186E+06	4.8011E+06	4.7988E+06	1.0695E+05	4.3452E+03	1.1482E+04	2.1934E+03
	Std	3.6604E+08	1.7077E+06	5.9593E+06	2.5721E+06	1.9393E+05	1.7139E+03	7.2586E+03	7.9347E+01
	Rank	8	5	7	6	4	2	3	1
F19	Best	4.7506E+03	5.1288E+03	6.4699E+03	5.6490E+03	5.1299E+03	4.5121E+03	5.0852E+03	2.4849E+03
	Avg	5.9860E+03	6.3524E+03	6.6515E+03	6.0029E+03	5.3720E+03	5.0431E+03	5.5031E+03	3.5464E+03
	Std	8.9336E+02	8.9209E+02	1.9080E+02	3.2796E+02	2.9674E+02	6.0944E+02	4.8821E+02	1.2969E+03
	Rank	5	7	8	6	3	2	4	1
F20	Best	3.5372E+03	3.1177E+03	3.1813E+03	3.3691E+03	2.9001E+03	3.0169E+03	3.1617E+03	2.5136E+03
	Avg	3.6555E+03	3.2395E+03	3.2885E+03	3.5420E+03	3.0209E+03	3.1326E+03	3.3213E+03	2.5938E+03
	Std	9.5124E+01	9.9611E+01	7.2293E+01	1.7597E+02	1.1162E+02	7.7493E+01	1.4275E+02	8.3624E+01
	Rank	8	4	5	7	2	3	6	1
F21	Best	2.8975E+04	2.7443E+04	3.1073E+04	2.5257E+04	2.6838E+04	1.7014E+04	1.6261E+04	7.2030E+03
	Avg	3.0308E+04	3.0554E+04	3.2512E+04	2.7318E+04	2.9010E+04	1.9066E+04	1.8500E+04	1.3637E+04
	Std	1.1579E+03	2.2007E+03	9.6970E+02	1.5657E+03	1.7959E+03	1.6758E+03	2.3146E+03	5.5275E+03
	Rank	6	7	8	4	5	3	2	1
F22	Best	4.0146E+03	3.7717E+03	3.7810E+03	3.8955E+03	3.6365E+03	3.8335E+03	3.6315E+03	3.1159E+03
	Avg	4.4003E+03	3.8149E+03	3.9304E+03	4.1166E+03	3.7299E+03	3.9677E+03	3.8219E+03	3.1517E+03
	Std	2.7683E+02	4.6466E+01	1.1741E+02	1.9481E+02	1.0615E+02	1.7590E+02	1.7212E+02	3.2483E+01
	Rank	8	3	5	7	2	6	4	1
F23	Best	4.8007E+03	4.3135E+03	4.2658E+03	4.9132E+03	4.3763E+03	4.3493E+03	4.5250E+03	4.0159E+03
	Avg	5.1692E+03	4.7310E+03	4.6800E+03	4.9646E+03	4.6214E+03	4.7365E+03	4.5705E+03	4.3151E+03
	Std	2.7420E+02	5.8390E+02	4.9537E+02	5.8790E+01	2.6404E+02	3.8043E+02	4.6508E+01	3.2444E+02
	Rank	8	5	4	7	3	6	2	1
F24	Best	9.5292E+03	4.6765E+03	4.6027E+03	6.4767E+03	4.1720E+03	3.1786E+03	3.1528E+03	3.1422E+03
	Avg	1.1884E+04	5.7583E+03	5.5506E+03	6.6788E+03	4.6597E+03	3.2697E+03	3.2063E+03	3.2219E+03
	Std	2.4724E+03	7.7302E+02	8.7766E+02	2.1784E+02	4.6581E+02	1.0042E+02	4.6358E+01	5.4176E+01
	Rank	8	6	5	7	4	3	1	2
F25	Best	2.0470E+04	1.5487E+04	1.6172E+04	1.8679E+04	1.2736E+04	1.4539E+04	1.6263E+04	9.1279E+03
	Avg	2.5238E+04	1.8591E+04	1.8351E+04	2.0598E+04	1.3736E+04	1.9982E+04	1.7532E+04	9.3409E+03
	Std	3.9562E+03	4.8713E+03	3.5792E+03	1.5874E+03	1.0458E+03	5.0807E+03	1.4224E+03	2.2093E+02
	Rank	8	5	4	7	2	6	3	1
F26	Best	4.0171E+03	3.7632E+03	3.9673E+03	3.7508E+03	3.8525E+03	3.2000E+03	3.1738E+03	3.2000E+03
	Avg	4.4848E+03	4.0831E+03	4.1337E+03	4.1520E+03	3.9812E+03	3.5778E+03	3.4184E+03	3.4063E+03
	Std	6.6520E+02	2.9851E+02	1.1852E+02	3.3224E+02	1.6569E+02	3.2713E+02	3.5035E+02	2.5661E+02
	Rank	8	5	6	7	4	3	2	1
F27	Best	1.0026E+04	5.8808E+03	5.7947E+03	6.1947E+03	4.7823E+03	3.2874E+03	3.2898E+03	3.2874E+03
	Avg	1.7443E+04	9.4214E+03	7.4928E+03	8.5150E+03	5.5494E+03	3.2953E+03	3.3133E+03	3.3058E+03
	Std	5.0251E+03	4.7589E+03	1.4923E+03	1.6129E+03	1.3058E+03	8.8779E+00	1.6900E+01	1.7743E+01
	Rank	8	7	5	6	4	1	3	2
F28	Best	1.2195E+04	7.9610E+03	8.4826E+03	9.7899E+03	6.6392E+03	5.8516E+03	6.9908E+03	4.0686E+03
	Avg	1.4170E+04	8.9221E+03	9.2074E+03	1.0293E+04	7.3185E+03	6.7316E+03	7.3267E+03	4.4266E+03
	Std	2.8708E+03	6.6130E+02	6.0040E+02	3.9135E+02	6.9860E+02	9.6459E+02	3.6017E+02	4.6202E+02
	Rank	8	5	6	7	3	2	4	1
F29	Best	5.5469E+08	1.6549E+07	9.1512E+06	6.3844E+07	1.2371E+06	4.5489E+03	5.4792E+03	4.1986E+03
	Avg	7.5685E+08	2.5576E+07	1.4285E+08	9.1258E+07	7.4923E+06	4.8361E+03	1.0472E+04	4.3872E+03
	Std	1.6456E+08	9.2373E+06	2.3804E+08	2.7638E+07	1.0097E+07	2.0753E+02	4.4159E+03	1.9108E+02
	Rank	8	5	7	6	4	2	3	1

**Table A13:** Experiments comparing EECO with other competing algorithms (10D).

No.	Index	EECO	EDECO	ISGTOA	APSM-jSO	LSHADE-SPACMA	EOSMA	GLSRIME	EPSCA	ESLPSO
F1	Best	1.0000E+02	3.6458E+05	1.2219E+04	4.3728E+02	2.1982E+04	6.6664E+06	1.4381E+04	1.2102E+02	9.5769E+03
	Avg	1.0000E+02	4.6495E+06	6.8504E+04	3.1539E+03	4.0940E+04	1.8883E+07	7.2018E+05	1.5336E+03	4.1541E+04
	Std	0.0000E+00	3.8003E+06	3.5297E+04	2.3017E+03	1.3861E+04	1.5049E+07	1.1249E+06	1.3630E+03	2.7714E+04
	Rank	1	8	6	3	4	9	7	2	5
F2	Best	3.0000E+02	3.3388E+02	1.1758E+03	3.0003E+02	3.0000E+02	1.3322E+03	3.2315E+02	3.1696E+02	1.1840E+03
	Avg	3.0000E+02	7.8825E+02	3.2470E+03	3.0046E+02	3.0032E+02	2.7750E+03	4.8869E+02	1.0581E+03	2.1846E+03
	Std	0.0000E+00	4.7027E+02	1.4896E+03	8.4502E-01	4.9203E-01	1.0708E+03	1.3797E+02	1.2131E+03	7.0420E+02
	Rank	1	5	9	3	2	8	4	6	7
F3	Best	4.0000E+02	4.0058E+02	4.0376E+02	4.0345E+02	4.0300E+02	4.0660E+02	4.0109E+02	4.0554E+02	4.0407E+02
	Avg	4.0000E+02	4.0632E+02	4.1345E+02	4.0440E+02	4.0387E+02	4.0868E+02	4.0636E+02	4.1103E+02	4.0594E+02
	Std	4.1355E-13	3.0294E+00	2.2317E+01	6.7479E-01	4.3237E-01	1.4331E+00	2.1195E+00	1.6160E+01	7.6909E-01
	Rank	1	5	9	3	2	7	6	8	4
F4	Best	5.0199E+02	5.1145E+02	5.2820E+02	5.2013E+02	5.1273E+02	5.2306E+02	5.0732E+02	5.0398E+02	5.2666E+02
	Avg	5.0451E+02	5.2516E+02	5.3830E+02	5.3318E+02	5.2649E+02	5.3236E+02	5.1956E+02	5.1131E+02	5.3437E+02
	Std	1.7983E+00	1.0200E+01	6.8853E+00	7.9570E+00	6.0967E+00	4.3687E+00	8.4660E+00	6.7448E+00	4.3431E+00
	Rank	1	4	9	7	5	6	3	2	8
F5	Best	6.0000E+02	6.0201E+02	6.0035E+02	6.0008E+02	6.0041E+02	6.0312E+02	6.0045E+02	6.0000E+02	6.0011E+02
	Avg	6.0001E+02	6.0704E+02	6.0129E+02	6.0024E+02	6.0096E+02	6.0625E+02	6.0124E+02	6.0001E+02	6.0017E+02
	Std	3.7882E-02	5.9222E+00	1.4108E+00	1.2920E-01	2.7884E-01	2.1039E+00	1.0194E+00	1.1405E-02	5.0284E-02
	Rank	1	9	7	4	5	8	6	2	3
F6	Best	7.1089E+02	7.3103E+02	7.3385E+02	7.3895E+02	7.3565E+02	7.3668E+02	7.2249E+02	7.1345E+02	7.3729E+02
	Avg	7.1422E+02	7.4554E+02	7.4796E+02	7.4543E+02	7.4457E+02	7.5149E+02	7.3151E+02	7.2013E+02	7.4561E+02
	Std	3.3408E+00	9.9181E+00	7.4978E+00	4.6171E+00	5.7293E+00	7.1197E+00	5.3489E+00	4.6793E+00	5.0158E+00
	Rank	1	6	8	5	4	9	3	2	7
F7	Best	8.0199E+02	8.1484E+02	8.1718E+02	8.2414E+02	8.2062E+02	8.1715E+02	8.0500E+02	8.0398E+02	8.2633E+02
	Avg	8.0829E+02	8.3283E+02	8.3223E+02	8.3274E+02	8.3068E+02	8.3053E+02	8.1646E+02	8.0842E+02	8.3372E+02
	Std	4.8839E+00	1.0619E+01	9.4331E+00	6.7384E+00	4.4348E+00	7.4438E+00	6.9962E+00	3.3185E+00	4.2053E+00
	Rank	1	8	6	7	5	4	3	2	9
F8	Best	9.0000E+02	9.0197E+02	9.0004E+02	9.0000E+02	9.0021E+02	9.0564E+02	9.0028E+02	9.0000E+02	9.0001E+02
	Avg	9.0000E+02	9.2277E+02	9.0075E+02	9.0005E+02	9.0049E+02	9.2748E+02	9.0333E+02	9.0021E+02	9.0003E+02
	Std	0.0000E+00	2.0248E+01	1.5514E+00	5.9482E-02	2.4618E-01	1.7200E+01	5.5906E+00	4.5959E-01	1.6631E-02
	Rank	1	8	6	3	5	9	7	4	2
F9	Best	1.3769E+03	1.6057E+03	1.7620E+03	2.1993E+03	1.8103E+03	1.7452E+03	1.3686E+03	1.0424E+03	2.2841E+03
	Avg	1.6773E+03	2.1169E+03	2.2447E+03	2.5286E+03	2.2331E+03	2.2384E+03	1.6846E+03	1.5869E+03	2.6105E+03
	Std	1.9887E+02	3.5449E+02	2.7255E+02	1.7634E+02	2.4051E+02	1.8060E+02	2.7235E+02	3.1790E+02	1.9291E+02
	Rank	2	4	7	8	5	6	3	1	9
F10	Best	1.1010E+03	1.1074E+03	1.1140E+03	1.1053E+03	1.1083E+03	1.1300E+03	1.1073E+03	1.1020E+03	1.1064E+03
	Avg	1.1069E+03	1.1340E+03	1.1299E+03	1.1102E+03	1.1115E+03	1.1394E+03	1.1305E+03	1.1065E+03	1.1091E+03
	Std	6.5809E+00	1.4243E+01	8.5493E+00	3.5789E+00	2.9111E+00	8.5438E+00	4.8719E+01	4.2456E+00	2.0175E+00
	Rank	2	8	6	4	5	9	7	1	3
F11	Best	1.3186E+03	6.6787E+03	4.5962E+04	1.4754E+03	3.3895E+03	2.4878E+05	6.4074E+04	3.3699E+03	2.2412E+04
	Avg	1.5028E+03	4.2079E+05	1.1059E+06	3.1059E+03	6.3668E+03	1.3828E+06	2.2778E+06	2.2763E+04	1.9604E+05
	Std	2.0539E+02	8.0888E+05	1.2438E+06	2.0520E+03	2.4259E+03	1.1314E+06	2.2053E+06	2.2717E+04	1.4326E+05
	Rank	1	6	7	2	3	8	9	4	5
F12	Best	1.3054E+03	1.4321E+03	2.1589E+03	1.3156E+03	1.3333E+03	1.9172E+03	1.5638E+03	1.3323E+03	1.4027E+03
	Avg	1.4125E+03	2.3128E+03	7.3130E+03	1.3294E+03	1.3670E+03	4.1934E+03	1.2210E+04	8.3471E+03	1.6581E+03
	Std	1.3790E+02	6.4454E+02	5.0782E+03	7.3498E+00	2.8269E+01	1.7121E+03	1.0244E+04	8.1296E+03	1.6882E+02
	Rank	3	5	7	1	2	6	9	8	4
F13	Best	1.4010E+03	1.4353E+03	1.4545E+03	1.4225E+03	1.4229E+03	1.4705E+03	1.4368E+03	1.4392E+03	1.4297E+03
	Avg	1.4181E+03	1.4464E+03	1.5066E+03	1.4287E+03	1.4302E+03	1.5018E+03	3.5312E+03	3.5011E+03	1.4380E+03
	Std	1.2380E+01	1.0283E+01	3.2996E+01	3.7559E+00	3.3408E+00	2.4954E+01	3.6441E+03	2.5436E+03	6.3816E+00
	Rank	1	5	7	2	3	6	9	8	4
F14	Best	1.5012E+03	1.5213E+03	1.6320E+03	1.5036E+03	1.5039E+03	1.6968E+03	1.5405E+03	1.5063E+03	1.5128E+03
	Avg	1.5070E+03	1.5734E+03	2.3545E+03	1.5090E+03	1.5100E+03	1.9809E+03	5.5340E+03	5.7982E+03	1.5214E+03
	Std	7.3589E+00	3.3017E+01	8.2288E+02	3.1159E+00	3.5437E+00	1.9851E+02	5.5043E+03	5.7461E+03	7.3901E+00
	Rank	1	5	7	2	3	6	8	9	4
F15	Best	1.6006E+03	1.6471E+03	1.6436E+03	1.6113E+03	1.6302E+03	1.6231E+03	1.6030E+03	1.6008E+03	1.6197E+03
	Avg	1.6043E+03	1.7534E+03	1.7322E+03	1.6602E+03	1.6760E+03	1.6914E+03	1.7153E+03	1.6830E+03	1.6520E+03
	Std	5.0260E+00	8.5112E+01	6.0279E+01	4.3422E+01	3.5946E+01	5.6376E+01	1.0285E+02	5.9611E+01	2.8193E+01
	Rank	1	9	8	3	4	6	7	5	2
F16	Best	1.7017E+03	1.7517E+03	1.7503E+03	1.7465E+03	1.7478E+03	1.7511E+03	1.7233E+03	1.7020E+03	1.7488E+03
	Avg	1.7299E+03	1.7843E+03	1.7777E+03	1.7707E+03	1.7625E+03	1.7679E+03	1.7636E+03	1.7489E+03	1.7684E+03
	Std	1.9279E+01	2.5266E+01	1.8614E+01	1.5112E+01	8.6480E+00	1.1129E+01	5.2163E+01	3.3815E+01	1.1755E+01
	Rank	1	9	8	7	3	5	4	2	6
F17	Best	1.8020E+03	1.8977E+03	4.1121E+03	1.8262E+03	1.8429E+03	4.3540E+03	1.9697E+03	5.8071E+03	2.0069E+03
	Avg	1.8248E+03	2.6442E+03	1.6903E+04	1.8374E+03	1.8634E+03	9.7821E+03	1.5212E+04	1.7820E+04	2.4293E+03
	Std	1.3687E+01	1.5209E+03	1.3677E+04	2.1320E+01	1.1244E+01	4.9377E+03	1.0553E+04	1.4949E+04	4.2057E+02
	Rank	1	5	8	2	3	6	7	9	4
F18	Best	1.9001E+03	1.9076E+03	1.9427E+03	1.9031E+03	1.9056E+03	1.9591E+03	1.9893E+03	2.0423E+03	1.9070E+03
	Avg	1.9023E+03	1.9314E+03	2.9291E+03	1.9061E+03	1.9078E+03	2.1764E+03	1.2075E+04	1.2054E+04	1.9171E+03
	Std	1.4866E+00	2.6537E+01	1.3349E+03	1.4331E+00	1.2419E+00	1.9583E+02	9.1351E+03	9.8731E+03	1.6817E+01
	Rank	1	5	7	2	3	6	9	8	4
F19	Best	2.0003E+03	2.0572E+03	2.0407E+03	2.0288E+03	2.0420E+03	2.0510E+03	2.0083E+03	2.0043E+03	2.0365E+03
	Avg	2.0217E+03	2.1072E+03	2.0880E+03	2.0608E+03	2.0537E+03	2.0812E+03	2.0325E+03	2.0544E+03	2.0695E+03
	Std	1.2913E+01	3.2708E+01	4.8633E+01	1.6134E+01	8.2472E+00	1.4757E+01	1.5923E+01	5.1969E+01	2.4319E+01
	Rank	1	9	8	5	3	7	2	4	6
F20	Best	2.2000E+03	2.2045E+03	2.2028E+03	2.2011E+03	2.2051E+03	2.2045E+03	2.2028E+03	2.2026E+03	2.2011E+03
	Avg	2.2711E+03	2.2649E+03	2.2646E+03	2.2934E+03	2.2385E+03	2.2126E+03	2.2586E+03	2.2767E+03	2.2729E+03
	Std	5.0488E+01	5.9235E+01	6.3947E+01	5.9428E+01	4.0454E+01	6.3692E+00	6.1027E+01	5.2795E+01	6.8836E+01
	Rank	6	5	4	9	2	1	3	8	7
F21	Best	2.2000E+03	2.2223E+03	2.2121E+03	2.3005E+03	2.3030E+03	2.2347E+03	2.2420E+03	2.3004E+03	2.2004E+03
	Avg	2.2882E+03	2.2853E+03	2.3010E+03	2.3040E+03	2.3058E+03	2.2929E+03	2.3037E+03	2.3013E+03	2.2968E+03
	Std	3.1550E+01	3.5266E+01	2.4684E+01	2.0324E+00	1.1045E+00	2.5099E+01	1.7180E+01	6.9255E-01	2.6695E+01
	Rank	2	1	5	8	9	3	7	6	4
F22	Best	2.6043E+03	2.6119E+03	2.6158E+03	2.6173E+03	2.6226E+03	2.6252E+03	2.6100E+03	2.6049E+03	2.6205E+03
	Avg	2.6083E+03	2.6306E+03	2.6318E+03	2.6267E+03	2.6270E+03	2.6339E+03	2.6186E+03	2.6152E+03	2.6316E+03
	Std	2.7440E+00	1.0612E+01	8.8355E+00	7.7301E+00	3.8182E+00	6.2436E+00	6.1957E+00	6.1566E+00	5.6734E+00
	Rank	1	6	8	4	5	9	3	2	7
F23	Best	2.5000E+03	2.5650E+03	2.5292E+03	2.5278E+03	2.6075E+03	2.5519E+03	2.5015E+03	2.7336E+03	2.5017E+03
	Avg	2.7058E+03	2.7235E+03	2.7319E+03	2.7439E+03	2.7436E+03	2.6264E+03	2.7192E+03	2.7445E+03	2.7467E+03
	Std	8.3745E+01	6.4933E+01	7.6833E+01	6.0187E+01	3.9309E+01	6.9795E+01	8.8035E+01	7.5621E+00	6.7877E+01
	Rank	2	4	5	7	6	1	3	8	9
F24	Best	2.8977E+03	2.8982E+03	2.8997E+03	2.8983E+03	2.8983E+03	2.9095E+03	2.8993E+03	2.8979E+03	2.8978E+03
	Avg	2.9366E+03	2.9305E+03	2.9262E+03	2.9232E+03	2.9200E+03	2.9289E+03	2.9164E+03	2.9226E+03	2.9172E+03
	Std	2.9542E+01	2.1289E+01	2.3028E+01	2.3966E+01	2.3323E+01	1.5984E+01	2.3498E+01	2.2861E+01	2.3638E+01
	Rank	9	8	6	5	3	7	1	4	2
F25	Best	2.9000E+03	2.9475E+03	2.8323E+03	2.9000E+03	2.9004E+03	2.9359E+03	2.6252E+03	2.9065E+03	2.9002E+03
	Avg	2.9230E+03	3.0115E+03	2.9276E+03	2.9013E+03	2.9007E+03	2.9690E+03	2.9135E+03	2.9981E+03	2.9128E+03
	Std	5.3723E+01	3.6039E+01	4.4321E+01	4.5516E+00	1.4898E-01	2.0999E+01	8.7845E+01	2.1104E+02	2.1433E+01
	Rank	5	9	6	2	1	7	4	8	3
F26	Best	3.0884E+03	3.0911E+03	3.0899E+03	3.0890E+03	3.0902E+03	3.0917E+03	3.0886E+03	3.0890E+03	3.0890E+03
	Avg	3.0959E+03	3.0955E+03	3.0932E+03	3.0919E+03	3.0919E+03	3.0957E+03	3.0960E+03	3.0915E+03	3.0904E+03
	Std	1.0550E+01	2.7736E+00	1.8301E+00	2.8135E+00	1.8393E+00	2.6493E+00	3.2250E+00	2.8328E+00	1.8616E+00
	Rank	8	6	5	4	3	7	9	2	1
F27	Best	3.1000E+03	3.1769E+03	3.1708E+03	3.1004E+03	3.1015E+03	3.1737E+03	3.1162E+03	3.1709E+03	3.1059E+03
	Avg	3.2558E+03	3.3553E+03	3.2898E+03	3.2492E+03	3.1384E+03	3.1987E+03	3.3037E+03	3.3113E+03	3.2841E+03
	Std	4.7561E+01	7.9883E+01	9.3613E+01	1.3976E+02	7.7983E+01	2.0953E+01	1.2008E+02	1.0871E+02	1.2466E+02
	Rank	4	9	6	3	1	2	7	8	5
F28	Best	3.1285E+03	3.1834E+03	3.1613E+03	3.1930E+03	3.1784E+03	3.1833E+03	3.1528E+03	3.1428E+03	3.1789E+03
	Avg	3.1616E+03	3.2401E+03	3.2344E+03	3.2142E+03	3.2072E+03	3.2271E+03	3.2070E+03	3.1984E+03	3.2182E+03
	Std	4.3286E+01	3.8778E+01	4.3355E+01	1.2911E+01	2.1182E+01	2.2485E+01	4.2151E+01	5.6873E+01	2.9768E+01
	Rank	1	9	8	5	4	7	3	2	6
F29	Best	3.2239E+03	8.8689E+03	9.2557E+04	3.5261E+03	3.6195E+03	1.4603E+04	3.1605E+04	8.1467E+03	8.6256E+03
	Avg	3.4510E+03	3.2007E+05	5.6833E+05	5.8737E+04	4.7080E+03	2.1623E+05	4.7384E+05	5.1229E+05	7.5443E+04
	Std	4.7789E+02	2.9692E+05	1.9678E+05	2.1093E+05	7.9064E+02	2.1599E+05	5.4640E+05	4.1178E+05	2.2195E+05
	Rank	1	6	9	3	2	5	7	8	4

**Table A14:** Experiments comparing EECO with other competing algorithms (30D).

No.	Index	EECO	EDECO	ISGTOA	APSM-jSO	LSHADE-SPACMA	EOSMA	GLSRIME	EPSCA	ESLPSO
F1	Best	1.0000E+02	5.3039E+08	1.4421E+07	1.2706E+05	1.6057E+06	8.7951E+08	3.7752E+07	1.5624E+06	5.6446E+06
	Avg	1.0000E+02	1.5110E+09	5.1761E+07	4.8320E+05	2.2121E+06	1.6162E+09	9.8047E+07	6.5408E+06	8.8206E+06
	Std	5.3627E-10	7.3613E+08	3.6068E+07	3.5801E+05	4.4854E+05	3.6773E+08	4.8338E+07	4.6111E+06	1.8781E+06
	Rank	1	8	6	2	3	9	7	4	5
F2	Best	3.0000E+02	1.2279E+04	3.5890E+04	1.7257E+03	1.9999E+03	3.8464E+04	2.5898E+04	4.0182E+04	4.0100E+04
	Avg	3.0000E+02	2.3419E+04	5.4283E+04	3.2838E+03	4.5539E+03	7.0305E+04	4.3016E+04	6.2530E+04	5.5441E+04
	Std	6.9834E-13	8.0982E+03	9.5103E+03	1.3420E+03	1.9824E+03	1.7803E+04	1.1870E+04	1.3120E+04	1.1653E+04
	Rank	1	4	6	2	3	9	5	8	7
F3	Best	4.0000E+02	5.9254E+02	5.1367E+02	4.8860E+02	4.9001E+02	5.9540E+02	5.0785E+02	5.0512E+02	4.8920E+02
	Avg	4.0186E+02	6.9028E+02	5.5668E+02	4.9975E+02	4.9337E+02	7.0082E+02	5.6136E+02	5.3020E+02	5.0397E+02
	Std	2.0587E+00	7.6803E+01	2.3905E+01	1.0684E+01	5.3051E+00	5.8867E+01	4.8030E+01	1.3863E+01	1.3118E+01
	Rank	1	8	6	3	2	9	7	5	4
F4	Best	5.1990E+02	6.4144E+02	6.6666E+02	6.5917E+02	6.7267E+02	6.6119E+02	5.6884E+02	5.3682E+02	6.7528E+02
	Avg	5.4245E+02	7.1821E+02	7.2051E+02	6.8949E+02	6.9178E+02	6.9167E+02	6.2717E+02	5.5117E+02	6.9092E+02
	Std	2.4699E+01	3.1051E+01	1.8445E+01	1.5287E+01	1.1377E+01	2.1582E+01	3.3323E+01	1.5438E+01	7.9229E+00
	Rank	1	8	9	4	7	6	3	2	5
F5	Best	6.0122E+02	6.1833E+02	6.0770E+02	6.0096E+02	6.0228E+02	6.1529E+02	6.0775E+02	6.0065E+02	6.0219E+02
	Avg	6.0340E+02	6.3018E+02	6.1341E+02	6.0126E+02	6.0263E+02	6.2206E+02	6.1388E+02	6.0116E+02	6.0274E+02
	Std	1.7467E+00	7.1418E+00	4.4624E+00	2.5342E-01	3.3298E-01	4.3494E+00	3.8657E+00	3.8720E-01	2.9200E-01
	Rank	5	9	6	2	3	8	7	1	4
F6	Best	7.4415E+02	9.3163E+02	9.3913E+02	8.9475E+02	9.1084E+02	9.3761E+02	8.6369E+02	7.6922E+02	9.0261E+02
	Avg	7.6706E+02	1.0130E+03	9.6859E+02	9.2613E+02	9.3231E+02	9.7383E+02	9.2750E+02	7.9502E+02	9.2460E+02
	Std	1.6126E+01	4.9454E+01	1.5065E+01	1.3596E+01	1.3158E+01	2.7940E+01	3.8331E+01	1.5905E+01	1.2106E+01
	Rank	1	9	7	4	6	8	5	2	3
F7	Best	8.1094E+02	9.0226E+02	9.9055E+02	9.6383E+02	9.6640E+02	9.5808E+02	8.7527E+02	8.3155E+02	9.6139E+02
	Avg	8.2633E+02	9.8250E+02	1.0134E+03	9.8336E+02	9.9102E+02	9.9176E+02	9.0371E+02	8.5138E+02	9.9165E+02
	Std	1.0768E+01	3.4335E+01	1.8101E+01	1.2602E+01	1.2600E+01	1.9137E+01	1.8473E+01	1.7674E+01	1.0220E+01
	Rank	1	4	9	5	6	8	3	2	7
F8	Best	9.0235E+02	1.8289E+03	1.1045E+03	9.0124E+02	9.0917E+02	1.5291E+03	1.3816E+03	9.1027E+02	9.0839E+02
	Avg	1.0753E+03	3.7534E+03	1.5542E+03	9.0458E+02	9.1409E+02	2.1229E+03	2.7262E+03	9.3821E+02	9.1271E+02
	Std	2.6070E+02	1.4570E+03	2.9598E+02	2.9538E+00	3.7457E+00	5.9383E+02	1.5440E+03	2.3179E+01	2.9297E+00
	Rank	5	9	6	1	3	7	8	4	2
F9	Best	4.0675E+03	6.5101E+03	7.6171E+03	7.4353E+03	7.6053E+03	7.1897E+03	3.5790E+03	3.4376E+03	7.8424E+03
	Avg	5.0357E+03	7.4975E+03	8.5601E+03	8.5088E+03	8.4969E+03	8.1087E+03	4.7628E+03	4.3311E+03	8.5848E+03
	Std	5.9698E+02	6.6318E+02	3.7719E+02	4.5679E+02	3.6219E+02	4.0558E+02	7.5565E+02	6.1071E+02	3.4561E+02
	Rank	3	4	8	7	6	5	2	1	9
F10	Best	1.1318E+03	1.2920E+03	1.6673E+03	1.1848E+03	1.1983E+03	1.5687E+03	1.3084E+03	1.1905E+03	1.2305E+03
	Avg	1.1611E+03	1.4184E+03	2.0473E+03	1.2381E+03	1.2363E+03	1.8812E+03	1.4176E+03	1.2341E+03	1.2940E+03
	Std	2.1430E+01	8.9162E+01	2.3458E+02	2.6578E+01	2.4724E+01	1.9991E+02	5.7595E+01	2.3916E+01	3.8506E+01
	Rank	1	7	9	4	3	8	6	2	5
F11	Best	1.6653E+03	7.8723E+06	1.8725E+06	7.4811E+04	4.1210E+05	2.2139E+07	5.1398E+06	2.2234E+05	1.1290E+06
	Avg	2.3615E+03	3.5060E+07	7.2653E+06	3.1591E+05	6.3099E+05	5.2438E+07	2.9002E+07	9.6314E+05	3.4038E+06
	Std	4.0135E+02	2.4954E+07	5.2816E+06	1.9125E+05	1.9221E+05	1.8848E+07	2.8836E+07	6.9246E+05	1.3689E+06
	Rank	1	8	6	2	3	9	7	4	5
F12	Best	1.5151E+03	6.8309E+04	5.1006E+04	1.3354E+04	7.2208E+04	1.2932E+06	1.3033E+05	2.3028E+03	3.1282E+04
	Avg	2.2556E+03	1.7408E+05	1.0671E+05	2.4361E+04	1.1064E+05	5.9791E+06	6.4118E+05	1.6490E+04	3.5679E+05
	Std	6.0513E+02	6.6446E+04	4.0863E+04	8.1766E+03	3.3247E+04	3.9361E+06	6.7998E+05	1.5310E+04	2.2516E+05
	Rank	1	6	4	3	5	9	8	2	7
F13	Best	1.4290E+03	1.6480E+03	6.2522E+03	1.5233E+03	1.7046E+03	8.8463E+03	1.7184E+04	1.0452E+04	1.9220E+03
	Avg	1.4809E+03	2.1678E+03	5.7616E+04	1.5550E+03	1.8528E+03	4.4053E+04	6.7324E+04	6.0331E+04	2.2141E+03
	Std	3.7896E+01	1.0257E+03	5.1036E+04	1.8277E+01	1.1568E+02	1.6550E+04	9.2942E+04	5.4731E+04	4.0052E+02
	Rank	1	4	7	2	3	6	9	8	5
F14	Best	1.5598E+03	1.1776E+04	2.1726E+04	2.2031E+03	8.3951E+03	1.7641E+05	1.1149E+04	1.8018E+03	2.8439E+04
	Avg	1.6608E+03	2.9877E+04	5.0011E+04	2.6417E+03	1.5391E+04	6.9094E+05	1.0579E+05	6.7769E+03	6.4259E+04
	Std	4.9470E+01	1.2842E+04	2.9789E+04	3.0961E+02	3.7403E+03	3.5046E+05	7.5788E+04	7.5208E+03	3.5590E+04
	Rank	1	5	6	2	4	9	8	3	7
F15	Best	1.6027E+03	2.3269E+03	3.0603E+03	2.9586E+03	3.0544E+03	2.6997E+03	2.1477E+03	1.8036E+03	2.9860E+03
	Avg	1.8519E+03	3.1450E+03	3.4625E+03	3.3134E+03	3.3023E+03	3.2390E+03	2.6645E+03	2.2518E+03	3.2904E+03
	Std	2.1228E+02	2.9881E+02	2.5125E+02	1.8615E+02	1.6300E+02	2.2390E+02	2.4032E+02	3.0237E+02	1.9330E+02
	Rank	1	4	9	8	7	5	3	2	6
F16	Best	1.7361E+03	2.0277E+03	1.9647E+03	2.1222E+03	2.1678E+03	1.9465E+03	1.8512E+03	1.7472E+03	2.1890E+03
	Avg	1.8085E+03	2.2589E+03	2.2186E+03	2.3222E+03	2.3369E+03	2.1903E+03	2.1164E+03	1.9747E+03	2.3484E+03
	Std	7.7009E+01	1.5197E+02	1.8425E+02	1.3377E+02	6.5920E+01	1.2145E+02	1.8100E+02	1.8146E+02	9.5764E+01
	Rank	1	6	5	7	8	4	3	2	9
F17	Best	1.8689E+03	2.2311E+04	2.1272E+05	3.0625E+03	7.4247E+03	3.9751E+05	2.0559E+05	4.3165E+04	3.3972E+04
	Avg	1.9368E+03	8.2441E+04	6.6926E+05	4.6467E+03	2.1219E+04	9.9425E+05	1.3957E+06	1.6978E+06	1.0076E+05
	Std	5.3946E+01	4.2900E+04	5.5054E+05	1.8981E+03	8.5896E+03	3.9903E+05	1.6107E+06	1.8392E+06	4.6845E+04
	Rank	1	4	6	2	3	7	8	9	5
F18	Best	1.9224E+03	8.8543E+03	1.1727E+04	2.0927E+03	4.4990E+03	2.1800E+05	3.4797E+04	2.1061E+03	1.6010E+04
	Avg	1.9971E+03	1.1076E+05	5.0607E+04	2.4929E+03	7.3318E+03	9.0461E+05	4.2046E+05	1.1869E+04	4.6635E+04
	Std	5.4650E+01	1.1407E+05	2.4995E+04	8.3597E+02	2.4318E+03	5.1692E+05	2.6358E+05	1.0936E+04	2.0478E+04
	Rank	1	7	6	2	3	9	8	4	5
F19	Best	2.0256E+03	2.3527E+03	2.3649E+03	2.4896E+03	2.3920E+03	2.4831E+03	2.1343E+03	2.0434E+03	2.5506E+03
	Avg	2.0490E+03	2.6169E+03	2.7130E+03	2.7752E+03	2.7295E+03	2.6702E+03	2.3856E+03	2.3026E+03	2.7614E+03
	Std	1.6675E+01	1.4438E+02	1.8280E+02	1.3137E+02	1.2573E+02	1.1245E+02	1.6334E+02	1.5163E+02	1.2883E+02
	Rank	1	4	6	9	7	5	3	2	8
F20	Best	2.3131E+03	2.4240E+03	2.4295E+03	2.4519E+03	2.4579E+03	2.4561E+03	2.3778E+03	2.3329E+03	2.4697E+03
	Avg	2.3354E+03	2.4809E+03	2.4909E+03	2.4776E+03	2.4851E+03	2.4792E+03	2.4279E+03	2.3474E+03	2.4859E+03
	Std	2.8757E+01	2.9743E+01	2.6470E+01	1.4645E+01	1.2279E+01	1.3616E+01	2.7676E+01	1.1543E+01	7.8237E+00
	Rank	1	6	9	4	7	5	3	2	8
F21	Best	2.3000E+03	2.6673E+03	2.3304E+03	2.3114E+03	2.3171E+03	2.6017E+03	2.3303E+03	2.3172E+03	2.3190E+03
	Avg	2.4892E+03	6.1441E+03	2.9698E+03	2.3146E+03	2.3185E+03	2.6679E+03	4.6361E+03	5.2111E+03	2.3324E+03
	Std	7.3176E+02	3.0103E+03	2.0063E+03	1.8596E+00	9.4974E-01	4.2942E+01	2.1822E+03	1.6268E+03	2.9509E+01
	Rank	4	9	6	1	2	5	7	8	3
F22	Best	2.6611E+03	2.7771E+03	2.8080E+03	2.8176E+03	2.8092E+03	2.8073E+03	2.7374E+03	2.6827E+03	2.8209E+03
	Avg	2.6858E+03	2.8699E+03	2.8557E+03	2.8372E+03	2.8386E+03	2.8516E+03	2.7733E+03	2.6964E+03	2.8404E+03
	Std	2.3121E+01	3.3452E+01	2.0868E+01	1.3129E+01	1.2770E+01	2.4432E+01	2.1428E+01	1.0057E+01	9.0912E+00
	Rank	1	9	8	4	5	7	3	2	6
F23	Best	2.8326E+03	2.9641E+03	2.9789E+03	2.9556E+03	2.9881E+03	2.9586E+03	2.8866E+03	2.8426E+03	2.9910E+03
	Avg	2.8743E+03	3.0319E+03	3.0230E+03	3.0026E+03	3.0032E+03	3.0054E+03	2.9276E+03	2.8731E+03	3.0036E+03
	Std	3.4835E+01	3.7227E+01	2.7036E+01	1.7061E+01	8.9701E+00	2.2054E+01	2.0355E+01	1.8963E+01	8.5315E+00
	Rank	2	9	8	4	5	7	3	1	6
F24	Best	2.8755E+03	2.9742E+03	2.8980E+03	2.8876E+03	2.8878E+03	3.0226E+03	2.9078E+03	2.8944E+03	2.8882E+03
	Avg	2.8831E+03	3.0436E+03	2.9508E+03	2.8890E+03	2.8888E+03	3.0740E+03	2.9496E+03	2.9086E+03	2.8909E+03
	Std	1.6523E+01	4.8366E+01	2.4169E+01	2.1380E+00	7.2341E-01	4.5806E+01	2.9317E+01	1.0150E+01	2.7301E+00
	Rank	1	8	7	3	2	9	6	5	4
F25	Best	3.5533E+03	3.8738E+03	3.1771E+03	5.2000E+03	5.1425E+03	4.3885E+03	4.4044E+03	3.7982E+03	2.9376E+03
	Avg	3.9352E+03	5.4866E+03	5.1380E+03	5.3909E+03	5.3555E+03	5.5710E+03	4.9261E+03	4.0743E+03	5.0642E+03
	Std	2.2284E+02	6.8761E+02	8.4248E+02	1.0067E+02	1.1908E+02	3.6806E+02	3.1798E+02	1.3371E+02	8.7128E+02
	Rank	1	8	5	7	6	9	3	2	4
F26	Best	3.1264E+03	3.2424E+03	3.2228E+03	3.2167E+03	3.2197E+03	3.2827E+03	3.2199E+03	3.1986E+03	3.2203E+03
	Avg	3.1792E+03	3.2904E+03	3.2407E+03	3.2276E+03	3.2260E+03	3.3196E+03	3.2454E+03	3.2173E+03	3.2275E+03
	Std	1.8144E+01	4.4358E+01	1.2849E+01	7.9249E+00	4.9678E+00	2.7690E+01	1.6747E+01	1.0196E+01	7.7115E+00
	Rank	1	8	6	5	3	9	7	2	4
F27	Best	3.1000E+03	3.3173E+03	3.2940E+03	3.2298E+03	3.2246E+03	3.3488E+03	3.2542E+03	3.2694E+03	3.2303E+03
	Avg	3.1320E+03	3.4898E+03	3.3290E+03	3.2492E+03	3.2411E+03	3.4541E+03	3.3427E+03	3.2939E+03	3.2548E+03
	Std	5.6754E+01	9.1542E+01	2.4677E+01	1.5023E+01	1.4772E+01	7.4654E+01	5.3891E+01	1.4964E+01	2.3270E+01
	Rank	1	9	6	3	2	8	7	5	4
F28	Best	3.3836E+03	4.1280E+03	3.6505E+03	3.8266E+03	3.8807E+03	3.8818E+03	3.5660E+03	3.3980E+03	3.9226E+03
	Avg	3.5263E+03	4.4271E+03	4.1402E+03	4.0886E+03	4.0728E+03	4.1646E+03	3.9638E+03	3.6807E+03	4.0994E+03
	Std	9.7478E+01	1.9062E+02	2.1169E+02	1.1789E+02	1.0271E+02	1.4150E+02	2.3204E+02	1.9090E+02	1.3534E+02
	Rank	1	9	7	5	4	8	3	2	6
F29	Best	3.4105E+03	1.6772E+05	1.4034E+05	1.0620E+04	2.4450E+04	2.2141E+06	2.2512E+05	7.0122E+03	1.3130E+05
	Avg	3.7782E+03	1.4152E+06	7.0488E+05	2.0982E+04	4.3317E+04	5.2108E+06	3.3277E+06	1.3570E+04	2.9383E+05
	Std	3.7754E+02	1.2694E+06	5.1166E+05	7.3986E+03	1.1628E+04	2.5394E+06	2.1015E+06	5.9159E+03	1.4545E+05
	Rank	1	7	6	3	4	9	8	2	5

**Table A15:** Experiments comparing EECO with other competing algorithms (50D).

No.	Index	EECO	EDECO	ISGTOA	APSM-jSO	LSHADE-SPACMA	EOSMA	GLSRIME	EPSCA	ESLPSO
F1	Best	1.0000E+02	4.7925E+09	5.0198E+08	5.9074E+06	1.0837E+07	6.7255E+09	4.2879E+08	1.0000E+02	8.5347E+07
	Avg	1.0000E+02	9.5729E+09	1.0159E+09	1.3639E+07	1.3365E+07	1.0492E+10	1.1763E+09	8.3811E+08	1.0300E+08
	Std	7.6907E-07	3.5920E+09	4.1380E+08	5.3520E+06	2.2389E+06	2.0903E+09	4.8071E+08	8.4241E+08	1.1330E+07
	Rank	1	8	6	3	2	9	7	5	4
F2	Best	3.0000E+02	5.4017E+04	8.6516E+04	1.4551E+04	1.9793E+04	9.9104E+04	6.9286E+04	6.7978E+02	1.0575E+05
	Avg	3.0000E+02	7.0252E+04	1.2344E+05	2.2523E+04	5.2198E+04	1.5120E+05	1.2072E+05	1.6096E+05	1.5294E+05
	Std	1.9107E-08	1.3026E+04	1.8316E+04	5.0574E+03	3.1839E+04	2.9818E+04	2.4896E+04	9.2576E+04	2.1898E+04
	Rank	1	4	6	2	3	7	5	9	8
F3	Best	4.0000E+02	1.1518E+03	6.8489E+02	5.2103E+02	5.9828E+02	1.3513E+03	7.4444E+02	5.1495E+02	5.5752E+02
	Avg	4.0133E+02	1.7277E+03	7.7378E+02	6.1496E+02	6.2381E+02	1.7678E+03	8.3701E+02	7.0252E+02	6.2271E+02
	Std	1.9453E+00	5.2206E+02	6.6121E+01	3.1321E+01	8.9771E+00	3.9025E+02	6.3582E+01	1.0857E+02	3.0933E+01
	Rank	1	8	6	2	4	9	7	5	3
F4	Best	5.4079E+02	8.3901E+02	8.4461E+02	8.3498E+02	8.3647E+02	8.2578E+02	7.0069E+02	5.5373E+02	8.5124E+02
	Avg	5.6799E+02	9.3781E+02	9.3004E+02	8.6507E+02	8.7054E+02	8.9102E+02	7.9503E+02	6.4899E+02	8.7443E+02
	Std	2.3514E+01	5.1851E+01	4.1489E+01	1.3790E+01	1.4011E+01	3.7282E+01	5.5267E+01	5.0249E+01	1.4224E+01
	Rank	1	9	8	4	5	7	3	2	6
F5	Best	6.0322E+02	6.3535E+02	6.2021E+02	6.0167E+02	6.0249E+02	6.2630E+02	6.1959E+02	6.0367E+02	6.0427E+02
	Avg	6.0774E+02	6.4746E+02	6.2956E+02	6.0247E+02	6.0299E+02	6.3572E+02	6.3278E+02	6.0949E+02	6.0561E+02
	Std	3.3724E+00	7.6751E+00	5.5514E+00	3.8990E-01	2.2534E-01	6.1215E+00	1.1901E+01	3.5863E+00	5.0561E-01
	Rank	4	9	6	1	2	8	7	5	3
F6	Best	7.7267E+02	1.2171E+03	1.2151E+03	1.0815E+03	1.0811E+03	1.2471E+03	1.1150E+03	8.5295E+02	1.1103E+03
	Avg	8.4986E+02	1.4130E+03	1.2554E+03	1.1177E+03	1.1159E+03	1.3265E+03	1.2532E+03	9.8090E+02	1.1334E+03
	Std	4.2473E+01	1.0399E+02	2.4850E+01	1.6135E+01	1.9252E+01	5.0049E+01	7.8812E+01	5.4072E+01	1.5136E+01
	Rank	1	9	7	4	3	8	6	2	5
F7	Best	8.3582E+02	1.1011E+03	1.1710E+03	1.1249E+03	1.1405E+03	1.1430E+03	1.0207E+03	8.6467E+02	1.1534E+03
	Avg	8.7429E+02	1.2006E+03	1.2343E+03	1.1637E+03	1.1679E+03	1.1996E+03	1.1008E+03	9.5560E+02	1.1719E+03
	Std	1.6703E+01	5.3187E+01	3.1891E+01	1.5381E+01	1.5001E+01	3.3559E+01	4.0847E+01	7.3620E+01	1.0452E+01
	Rank	1	8	9	4	5	7	3	2	6
F8	Best	1.1323E+03	7.5727E+03	4.0409E+03	9.1082E+02	9.2766E+02	5.3319E+03	4.9679E+03	1.3227E+03	9.9687E+02
	Avg	1.4867E+03	1.3514E+04	7.3077E+03	9.3337E+02	9.3897E+02	8.3575E+03	9.9996E+03	1.8565E+03	1.0387E+03
	Std	2.6421E+02	3.4502E+03	1.7918E+03	1.3786E+01	7.1343E+00	2.1976E+03	3.9272E+03	5.1287E+02	2.4307E+01
	Rank	4	9	6	1	2	7	8	5	3
F9	Best	2.5378E+03	1.0920E+04	1.2725E+04	1.3719E+04	1.4265E+04	1.2941E+04	8.1320E+03	5.6998E+03	1.4106E+04
	Avg	7.0564E+03	1.2972E+04	1.4412E+04	1.5023E+04	1.4966E+04	1.4260E+04	9.7476E+03	7.9829E+03	1.4832E+04
	Std	1.7152E+03	9.5550E+02	8.1708E+02	4.5179E+02	3.6655E+02	4.8781E+02	9.4268E+02	1.3014E+03	2.8587E+02
	Rank	1	4	6	9	8	5	3	2	7
F10	Best	1.1726E+03	1.9963E+03	4.1271E+03	1.3163E+03	1.3650E+03	4.7506E+03	1.7751E+03	1.3326E+03	1.4419E+03
	Avg	1.2313E+03	2.4568E+03	6.1314E+03	1.3782E+03	1.4169E+03	5.4949E+03	2.0200E+03	2.2524E+03	1.5925E+03
	Std	3.7132E+01	3.0428E+02	1.2820E+03	4.8200E+01	3.4489E+01	6.4461E+02	1.6925E+02	9.9187E+02	8.0581E+01
	Rank	1	7	9	2	3	8	5	6	4
F11	Best	2.1612E+03	1.8362E+08	1.9031E+07	2.3705E+06	4.6020E+06	3.8819E+08	1.0328E+08	9.9226E+05	2.1062E+07
	Avg	3.6113E+03	4.2147E+08	6.3942E+07	7.7817E+06	6.9384E+06	6.3808E+08	2.9363E+08	3.1844E+07	3.2462E+07
	Std	7.8086E+02	1.4560E+08	2.2229E+07	4.7176E+06	1.9812E+06	2.5986E+08	1.8555E+08	2.8985E+07	9.3116E+06
	Rank	1	8	6	3	2	9	7	4	5
F12	Best	2.0979E+03	9.9271E+05	2.0001E+05	5.0219E+04	3.2898E+05	8.5759E+06	6.4451E+05	2.2504E+03	3.8586E+05
	Avg	3.1305E+03	3.9607E+06	5.7247E+05	7.4997E+04	5.8056E+05	4.3484E+07	5.0799E+06	3.9705E+04	9.6383E+05
	Std	6.7394E+02	2.2097E+06	3.6294E+05	1.7410E+04	1.8887E+05	2.1749E+07	2.9461E+06	3.2215E+04	5.2587E+05
	Rank	1	7	4	3	5	9	8	2	6
F13	Best	1.5016E+03	5.6567E+03	1.2889E+05	1.7467E+03	3.5658E+03	9.0907E+04	1.2677E+05	3.4101E+04	8.0263E+03
	Avg	1.6079E+03	3.7291E+04	5.0575E+05	1.8468E+03	5.3096E+03	3.7901E+05	5.6832E+05	6.1092E+05	1.9104E+04
	Std	1.0883E+02	4.1427E+04	3.4244E+05	6.7422E+01	1.0260E+03	1.7963E+05	4.1996E+05	8.0036E+05	1.6517E+04
	Rank	1	5	7	2	3	6	8	9	4
F14	Best	1.6537E+03	4.6546E+04	3.7992E+04	6.9041E+03	6.8470E+04	1.9335E+06	2.2232E+05	1.9914E+03	8.2942E+04
	Avg	1.8610E+03	1.3694E+05	6.6119E+04	1.6564E+04	1.2449E+05	5.0691E+06	1.1459E+06	7.3151E+03	2.3992E+05
	Std	1.0930E+02	6.4251E+04	2.0375E+04	4.9806E+03	4.3488E+04	3.1616E+06	7.3247E+05	7.1341E+03	1.5834E+05
	Rank	1	6	4	3	5	9	8	2	7
F15	Best	1.7283E+03	3.3842E+03	3.6473E+03	4.2071E+03	4.1188E+03	3.8098E+03	2.8629E+03	2.5407E+03	4.3911E+03
	Avg	2.0247E+03	4.2565E+03	4.7004E+03	4.6614E+03	4.6935E+03	4.4357E+03	3.4765E+03	3.0336E+03	4.8548E+03
	Std	1.9968E+02	4.1388E+02	4.9963E+02	2.3479E+02	2.6088E+02	3.4461E+02	4.4703E+02	4.1882E+02	2.2153E+02
	Rank	1	4	8	6	7	5	3	2	9
F16	Best	1.8389E+03	3.4535E+03	3.1547E+03	3.1146E+03	3.4285E+03	2.9547E+03	2.7750E+03	2.3718E+03	3.3046E+03
	Avg	2.0337E+03	3.7373E+03	3.6677E+03	3.7161E+03	3.7281E+03	3.4975E+03	3.3136E+03	2.9548E+03	3.7265E+03
	Std	1.1829E+02	1.8860E+02	3.5955E+02	2.1162E+02	1.5927E+02	2.5763E+02	2.8519E+02	2.6549E+02	2.1161E+02
	Rank	1	9	5	6	8	4	3	2	7
F17	Best	1.8903E+03	1.0738E+05	1.4278E+06	1.2394E+04	4.5180E+04	1.8088E+06	3.5447E+05	5.1708E+05	1.7988E+05
	Avg	2.0273E+03	4.5207E+05	3.9660E+06	2.4129E+04	7.0902E+04	3.6671E+06	5.6955E+06	3.0804E+06	3.0443E+05
	Std	9.8536E+01	2.8309E+05	2.0673E+06	7.6372E+03	1.4120E+04	1.7237E+06	6.2002E+06	3.4610E+06	1.3577E+05
	Rank	1	5	8	2	3	7	9	6	4
F18	Best	1.9845E+03	2.3113E+04	5.9957E+04	7.3891E+03	3.5872E+04	5.2394E+05	1.9849E+05	2.1223E+03	1.0013E+05
	Avg	2.0477E+03	1.8981E+05	1.7458E+05	1.2745E+04	5.4590E+04	3.0907E+06	3.5353E+06	1.4093E+04	1.9283E+05
	Std	5.2258E+01	1.5998E+05	1.2487E+05	4.2891E+03	1.3250E+04	1.8501E+06	4.0876E+06	1.0908E+04	1.2818E+05
	Rank	1	6	5	2	4	8	9	3	7
F19	Best	2.0485E+03	2.8216E+03	3.7080E+03	3.4971E+03	3.2913E+03	3.3173E+03	2.8268E+03	2.4750E+03	3.6671E+03
	Avg	2.1193E+03	3.5595E+03	3.9565E+03	3.9543E+03	3.8949E+03	3.6939E+03	3.0847E+03	3.0751E+03	3.9739E+03
	Std	5.4049E+01	2.8791E+02	1.7104E+02	2.0482E+02	2.3720E+02	2.1436E+02	2.2873E+02	3.5762E+02	1.8028E+02
	Rank	1	4	8	7	6	5	3	2	9
F20	Best	2.3290E+03	2.5843E+03	2.6638E+03	2.5943E+03	2.6313E+03	2.6240E+03	2.5407E+03	2.3668E+03	2.6548E+03
	Avg	2.3575E+03	2.7046E+03	2.7323E+03	2.6657E+03	2.6709E+03	2.6851E+03	2.5873E+03	2.4609E+03	2.6797E+03
	Std	1.4863E+01	5.7673E+01	2.4706E+01	2.2249E+01	1.8941E+01	2.9941E+01	3.3609E+01	6.4711E+01	1.2923E+01
	Rank	1	8	9	4	5	7	3	2	6
F21	Best	2.3046E+03	1.2373E+04	1.5197E+04	2.7784E+03	1.4386E+04	4.9149E+03	9.3960E+03	7.9801E+03	1.5814E+04
	Avg	7.8366E+03	1.4490E+04	1.6522E+04	1.5718E+04	1.5978E+04	1.3243E+04	1.0749E+04	9.6922E+03	1.6400E+04
	Std	2.9475E+03	9.3920E+02	5.2400E+02	3.5991E+03	7.6138E+02	4.5054E+03	8.3400E+02	1.0018E+03	2.9268E+02
	Rank	1	5	9	6	7	4	3	2	8
F22	Best	2.7553E+03	3.1573E+03	3.0897E+03	3.0833E+03	3.0528E+03	3.1440E+03	3.0060E+03	2.8151E+03	3.0752E+03
	Avg	2.8522E+03	3.2315E+03	3.1659E+03	3.1027E+03	3.0968E+03	3.1808E+03	3.0702E+03	2.9004E+03	3.1079E+03
	Std	6.4461E+01	6.2271E+01	4.3980E+01	1.2164E+01	1.7536E+01	2.8850E+01	5.6650E+01	4.1724E+01	1.4814E+01
	Rank	1	9	7	5	4	8	3	2	6
F23	Best	2.9611E+03	3.2354E+03	3.3081E+03	3.2334E+03	3.2251E+03	3.2558E+03	3.0903E+03	2.9768E+03	3.2238E+03
	Avg	3.0461E+03	3.3423E+03	3.3377E+03	3.2658E+03	3.2550E+03	3.3009E+03	3.2048E+03	3.0482E+03	3.2629E+03
	Std	6.4960E+01	6.7906E+01	1.9794E+01	1.7941E+01	1.6958E+01	2.9647E+01	5.3517E+01	3.7894E+01	1.9413E+01
	Rank	1	9	8	6	4	7	3	2	5
F24	Best	2.9312E+03	3.5671E+03	3.1888E+03	3.0350E+03	3.0418E+03	3.7865E+03	3.2044E+03	3.0687E+03	3.0678E+03
	Avg	2.9720E+03	3.8731E+03	3.3337E+03	3.0859E+03	3.0501E+03	4.1261E+03	3.4075E+03	3.1902E+03	3.1000E+03
	Std	2.2055E+01	2.2550E+02	9.8461E+01	2.4401E+01	4.3327E+00	2.2517E+02	2.0445E+02	9.3950E+01	2.0350E+01
	Rank	1	8	6	3	2	9	7	5	4
F25	Best	2.9000E+03	7.8482E+03	7.3357E+03	6.8484E+03	6.9900E+03	7.8161E+03	6.5302E+03	4.7513E+03	7.0961E+03
	Avg	4.9613E+03	8.8606E+03	8.1295E+03	7.2052E+03	7.2100E+03	8.2728E+03	7.2049E+03	5.3230E+03	7.3853E+03
	Std	7.5099E+02	7.6881E+02	4.5846E+02	1.8946E+02	1.4696E+02	3.1546E+02	6.4412E+02	4.1034E+02	1.3376E+02
	Rank	1	9	7	4	5	8	3	2	6
F26	Best	3.1532E+03	3.5674E+03	3.4093E+03	3.2732E+03	3.2575E+03	3.7077E+03	3.4227E+03	3.3322E+03	3.2747E+03
	Avg	3.2543E+03	3.7453E+03	3.5588E+03	3.3294E+03	3.2890E+03	3.8844E+03	3.5860E+03	3.4475E+03	3.3690E+03
	Std	1.3207E+02	1.6783E+02	1.0584E+02	4.0700E+01	2.4961E+01	8.7655E+01	8.7932E+01	7.6648E+01	5.0872E+01
	Rank	1	8	6	3	2	9	7	5	4
F27	Best	3.2635E+03	3.8428E+03	3.5801E+03	3.3234E+03	3.2985E+03	4.3417E+03	3.4905E+03	3.3152E+03	3.3572E+03
	Avg	3.2976E+03	4.7429E+03	3.7356E+03	3.3514E+03	3.3233E+03	4.8269E+03	3.8602E+03	3.6566E+03	3.3873E+03
	Std	9.4362E+00	4.8091E+02	1.0505E+02	1.8705E+01	1.3083E+01	3.0065E+02	3.3498E+02	2.9360E+02	2.8164E+01
	Rank	1	8	6	3	2	9	7	5	4
F28	Best	3.2211E+03	4.9510E+03	4.2507E+03	4.7490E+03	4.7655E+03	4.8872E+03	4.6196E+03	3.8950E+03	4.6079E+03
	Avg	3.4463E+03	5.9246E+03	5.1588E+03	4.9660E+03	4.9498E+03	5.4116E+03	4.9670E+03	4.2825E+03	5.0258E+03
	Std	1.4832E+02	6.0421E+02	5.5983E+02	1.4004E+02	9.5998E+01	2.5365E+02	3.1217E+02	3.6965E+02	2.2539E+02
	Rank	1	9	7	4	3	8	5	2	6
F29	Best	3.4159E+03	2.6903E+07	2.0703E+07	3.0284E+06	3.6764E+06	9.8686E+07	5.2163E+07	8.1890E+05	1.1798E+07
	Avg	4.3805E+03	4.7821E+07	3.0285E+07	4.3502E+06	4.4486E+06	1.4598E+08	9.8333E+07	2.6875E+06	1.8853E+07
	Std	9.9589E+02	1.7872E+07	7.1760E+06	1.1703E+06	5.8131E+05	3.5820E+07	3.8102E+07	1.1125E+06	6.8320E+06
	Rank	1	7	6	3	4	9	8	2	5

**Table A16:** Experiments comparing EECO with other competing algorithms (100D).

No.	Index	EECO	EDECO	ISGTOA	APSM-jSO	LSHADE-SPACMA	EOSMA	GLSRIME	EPSCA	ESLPSO
F1	Best	1.0000E+02	4.8379E+10	1.3587E+10	3.1413E+08	1.1784E+08	4.8564E+10	1.9190E+10	7.1108E+05	1.3047E+09
	Avg	2.0403E+02	6.1811E+10	1.8451E+10	5.3099E+08	1.2473E+08	5.7195E+10	2.4351E+10	7.4247E+09	1.5611E+09
	Std	2.3260E+02	1.8831E+10	4.5820E+09	1.9969E+08	9.6664E+06	5.9532E+09	3.7010E+09	1.0261E+10	2.7030E+08
	Rank	1	9	6	3	2	8	7	5	4
F2	Best	3.0018E+02	1.5782E+05	2.6064E+05	8.9129E+04	3.0264E+05	3.2370E+05	3.7860E+05	8.2013E+02	3.0151E+05
	Avg	3.0082E+02	1.8220E+05	2.9572E+05	9.9055E+04	3.5234E+05	3.9258E+05	4.4916E+05	2.9782E+05	3.8888E+05
	Std	4.5854E-01	3.1844E+04	2.5915E+04	1.0635E+04	4.5239E+04	6.4069E+04	6.3161E+04	2.9233E+05	5.7859E+04
	Rank	1	3	4	2	6	8	9	5	7
F3	Best	4.0000E+02	4.2224E+03	2.0489E+03	8.5845E+02	7.2758E+02	5.4389E+03	2.7096E+03	7.2996E+02	1.0277E+03
	Avg	4.0319E+02	6.9339E+03	2.3820E+03	9.0933E+02	7.4853E+02	7.3769E+03	3.1728E+03	1.2903E+03	1.0918E+03
	Std	1.7829E+00	2.6087E+03	2.0093E+02	3.7777E+01	1.7741E+01	1.7635E+03	3.4369E+02	7.5332E+02	5.7149E+01
	Rank	1	8	6	3	2	9	7	5	4
F4	Best	6.7511E+02	1.5015E+03	1.4864E+03	1.2944E+03	1.2912E+03	1.3964E+03	1.3612E+03	9.9549E+02	1.3178E+03
	Avg	7.1033E+02	1.6051E+03	1.6202E+03	1.3281E+03	1.3186E+03	1.4738E+03	1.4419E+03	1.1009E+03	1.3766E+03
	Std	3.4856E+01	6.6516E+01	7.9081E+01	1.9652E+01	2.3920E+01	6.8810E+01	6.3334E+01	1.0250E+02	3.5681E+01
	Rank	1	8	9	4	3	7	6	2	5
F5	Best	6.1293E+02	6.6694E+02	6.4787E+02	6.0590E+02	6.0413E+02	6.5171E+02	6.4725E+02	6.1792E+02	6.1095E+02
	Avg	6.1435E+02	6.7373E+02	6.5577E+02	6.0704E+02	6.0465E+02	6.5622E+02	6.7081E+02	6.2358E+02	6.1184E+02
	Std	1.1095E+00	7.4344E+00	4.9464E+00	8.6795E-01	3.5732E-01	2.6448E+00	1.6396E+01	5.4963E+00	5.9642E-01
	Rank	4	9	6	2	1	7	8	5	3
F6	Best	9.7962E+02	2.6367E+03	2.0705E+03	1.6167E+03	1.6070E+03	2.3544E+03	2.3087E+03	1.6258E+03	1.7068E+03
	Avg	1.1300E+03	2.8628E+03	2.2378E+03	1.6528E+03	1.6303E+03	2.5699E+03	2.4029E+03	1.8299E+03	1.7299E+03
	Std	1.8465E+02	1.5072E+02	1.1641E+02	2.5044E+01	2.1430E+01	1.4196E+02	8.8462E+01	1.5337E+02	2.2737E+01
	Rank	1	9	6	3	2	8	7	5	4
F7	Best	9.9203E+02	1.8884E+03	1.8376E+03	1.6226E+03	1.5913E+03	1.7625E+03	1.5804E+03	1.0066E+03	1.6344E+03
	Avg	1.0784E+03	1.9869E+03	1.9361E+03	1.6561E+03	1.6129E+03	1.8331E+03	1.7335E+03	1.1637E+03	1.6832E+03
	Std	6.6466E+01	9.0131E+01	5.8657E+01	2.2275E+01	1.8455E+01	6.6286E+01	9.8631E+01	1.8091E+02	3.2922E+01
	Rank	1	9	8	4	3	7	6	2	5
F8	Best	3.4202E+03	4.0886E+04	3.4414E+04	1.4879E+03	1.0764E+03	3.1307E+04	3.8552E+04	7.8049E+03	2.2476E+03
	Avg	3.8428E+03	4.4753E+04	3.8439E+04	1.7385E+03	1.1256E+03	3.5944E+04	4.9874E+04	1.1314E+04	2.8805E+03
	Std	4.2226E+02	3.2750E+03	4.6160E+03	2.2207E+02	4.6227E+01	3.4154E+03	1.1665E+04	3.2000E+03	5.2043E+02
	Rank	4	8	7	2	1	6	9	5	3
F9	Best	1.5353E+04	2.7140E+04	3.1479E+04	3.1038E+04	3.1784E+04	2.9967E+04	2.2370E+04	1.2564E+04	3.1010E+04
	Avg	1.6276E+04	2.8948E+04	3.1646E+04	3.1683E+04	3.2057E+04	3.0463E+04	2.3453E+04	1.8019E+04	3.2042E+04
	Std	9.8458E+02	1.1696E+03	2.0815E+02	5.2469E+02	2.5004E+02	6.6445E+02	6.7190E+02	4.0257E+03	6.4395E+02
	Rank	1	4	6	7	9	5	3	2	8
F10	Best	1.8790E+03	2.3193E+04	7.6848E+04	2.9050E+03	4.3742E+03	5.3742E+04	2.7795E+04	2.4983E+03	6.4047E+03
	Avg	2.0621E+03	2.7056E+04	8.8803E+04	3.4129E+03	5.1767E+03	9.2718E+04	4.1006E+04	2.0006E+04	9.3371E+03
	Std	1.8346E+02	6.5231E+03	1.1878E+04	4.1779E+02	8.0118E+02	2.5721E+04	1.3080E+04	1.8045E+04	2.6675E+03
	Rank	1	6	8	2	3	9	7	5	4
F11	Best	7.9353E+03	4.0450E+09	9.7195E+08	1.0456E+08	8.1819E+07	7.2926E+09	1.6969E+09	2.5597E+07	3.3007E+08
	Avg	1.5437E+04	5.5766E+09	1.1646E+09	1.2189E+08	1.0070E+08	8.5510E+09	2.9794E+09	5.1945E+08	4.2023E+08
	Std	6.0307E+03	1.6371E+09	2.3091E+08	1.8941E+07	1.7360E+07	1.8395E+09	8.4621E+08	6.7182E+08	8.4358E+07
	Rank	1	8	6	3	2	9	7	5	4
F12	Best	4.7620E+03	3.5249E+07	3.4480E+06	8.9341E+04	9.8458E+05	1.9712E+08	3.0338E+07	6.5771E+03	3.8267E+06
	Avg	5.5317E+03	1.1336E+08	4.3873E+06	1.0032E+05	1.2322E+06	3.0777E+08	6.4860E+07	1.2807E+06	4.8913E+06
	Std	8.7238E+02	5.8478E+07	5.5463E+05	1.1581E+04	2.0781E+05	8.2320E+07	2.0994E+07	1.2290E+06	8.6906E+05
	Rank	1	8	5	2	3	9	7	4	6
F13	Best	1.6439E+03	2.2525E+05	4.6082E+06	2.3156E+04	9.4020E+04	4.2325E+06	2.3796E+06	2.0997E+05	2.3300E+05
	Avg	1.7949E+03	3.4803E+05	7.2496E+06	3.0468E+04	1.3385E+05	6.0535E+06	8.3617E+06	2.2359E+06	3.7233E+05
	Std	1.3081E+02	1.1378E+05	3.0887E+06	7.2471E+03	2.4750E+04	1.7004E+06	5.8516E+06	1.8871E+06	9.9123E+04
	Rank	1	4	8	2	3	7	9	6	5
F14	Best	1.6862E+03	1.1581E+06	2.0658E+05	4.4076E+04	3.8872E+05	1.6547E+07	7.4203E+06	2.2706E+03	6.8248E+05
	Avg	1.8197E+03	3.3236E+06	3.5489E+05	5.0764E+04	4.9814E+05	3.3258E+07	9.4527E+06	2.2553E+04	8.3808E+05
	Std	1.3665E+02	1.8988E+06	1.7746E+05	6.3644E+03	1.1286E+05	1.4796E+07	1.6281E+06	1.9145E+04	1.5439E+05
	Rank	1	7	4	3	5	9	8	2	6
F15	Best	2.3393E+03	7.9519E+03	7.9858E+03	9.1881E+03	8.9628E+03	7.3275E+03	6.3181E+03	4.7992E+03	9.5017E+03
	Avg	3.0949E+03	8.8414E+03	9.5648E+03	9.6219E+03	9.5800E+03	8.7502E+03	7.2218E+03	5.3769E+03	9.6866E+03
	Std	8.1751E+02	7.5517E+02	1.2259E+03	2.5685E+02	4.0997E+02	1.0843E+03	6.8335E+02	5.0690E+02	2.0314E+02
	Rank	1	5	6	8	7	4	3	2	9
F16	Best	1.9698E+03	4.6715E+03	5.9715E+03	6.7170E+03	6.3172E+03	6.4655E+03	5.6248E+03	3.9034E+03	6.5312E+03
	Avg	2.4605E+03	6.2705E+03	7.0811E+03	7.0130E+03	6.7541E+03	6.6535E+03	5.9590E+03	4.7150E+03	7.0159E+03
	Std	4.3992E+02	9.3236E+02	6.6807E+02	1.7051E+02	2.4617E+02	1.3840E+02	4.2303E+02	1.2374E+03	3.0398E+02
	Rank	1	4	9	7	6	5	3	2	8
F17	Best	1.9604E+03	2.6923E+05	4.4372E+06	7.5469E+04	1.8963E+05	5.0657E+06	1.1563E+07	3.8964E+05	5.8576E+05
	Avg	2.0826E+03	7.8960E+05	7.1941E+06	9.8189E+04	2.7589E+05	8.6633E+06	1.4854E+07	3.1946E+06	8.2113E+05
	Std	7.2829E+01	4.0146E+05	2.4483E+06	1.5079E+04	7.4585E+04	2.2780E+06	2.6954E+06	4.1243E+06	2.0723E+05
	Rank	1	4	7	2	3	8	9	6	5
F18	Best	2.1820E+03	6.2172E+06	1.7909E+06	2.4678E+05	7.6834E+05	1.1497E+07	2.5653E+07	2.4024E+03	8.0797E+05
	Avg	2.2686E+03	1.7037E+07	3.2919E+06	2.9100E+05	8.5443E+05	4.0115E+07	3.7473E+07	1.1773E+05	1.2398E+06
	Std	1.4156E+02	9.9133E+06	1.5461E+06	5.9895E+04	9.6137E+04	1.7362E+07	1.2156E+07	1.3239E+05	3.3106E+05
	Rank	1	7	6	3	4	9	8	2	5
F19	Best	2.4218E+03	5.8890E+03	6.2083E+03	7.1130E+03	7.1780E+03	6.7053E+03	4.9963E+03	4.3177E+03	7.2462E+03
	Avg	2.9438E+03	6.3470E+03	7.1617E+03	7.3074E+03	7.3910E+03	7.0502E+03	5.6282E+03	4.6207E+03	7.3965E+03
	Std	9.2056E+02	3.7851E+02	5.3768E+02	2.2998E+02	1.4397E+02	2.5093E+02	4.8177E+02	3.2189E+02	1.7397E+02
	Rank	1	4	6	7	8	5	3	2	9
F20	Best	2.4965E+03	3.3679E+03	3.3347E+03	3.1707E+03	3.1223E+03	3.3071E+03	3.1379E+03	2.7885E+03	3.1600E+03
	Avg	2.5726E+03	3.4540E+03	3.4234E+03	3.1876E+03	3.1469E+03	3.3611E+03	3.2538E+03	2.8700E+03	3.1953E+03
	Std	5.2607E+01	6.0940E+01	7.9293E+01	1.2574E+01	1.7170E+01	4.6798E+01	1.0207E+02	5.5279E+01	3.1818E+01
	Rank	1	9	8	4	3	7	6	2	5
F21	Best	8.4600E+03	2.9367E+04	3.2994E+04	3.3590E+04	3.3766E+04	3.1976E+04	2.3169E+04	1.5031E+04	3.3065E+04
	Avg	1.2134E+04	2.9878E+04	3.3717E+04	3.4111E+04	3.4417E+04	3.2772E+04	2.5671E+04	1.9508E+04	3.3938E+04
	Std	4.7472E+03	4.4173E+02	5.9534E+02	5.7917E+02	4.7144E+02	5.3976E+02	1.8707E+03	3.4046E+03	7.3430E+02
	Rank	1	4	6	8	9	5	3	2	7
F22	Best	3.0687E+03	4.0653E+03	3.9400E+03	3.6925E+03	3.6493E+03	3.9033E+03	3.7359E+03	3.3139E+03	3.6922E+03
	Avg	3.2777E+03	4.1762E+03	3.9789E+03	3.7091E+03	3.6894E+03	3.9989E+03	3.8429E+03	3.3942E+03	3.7510E+03
	Std	2.3454E+02	8.0793E+01	4.5017E+01	2.2184E+01	2.3338E+01	7.3385E+01	7.4340E+01	1.0166E+02	4.6763E+01
	Rank	1	9	7	4	3	8	6	2	5
F23	Best	3.6738E+03	4.6981E+03	4.4260E+03	4.0759E+03	4.0977E+03	4.5645E+03	4.1381E+03	3.5711E+03	4.1660E+03
	Avg	4.0880E+03	4.8790E+03	4.5490E+03	4.1261E+03	4.1159E+03	4.7293E+03	4.3823E+03	3.7734E+03	4.1818E+03
	Std	3.1774E+02	1.4722E+02	8.3143E+01	3.6417E+01	1.4013E+01	1.5559E+02	1.7018E+02	2.0642E+02	1.7365E+01
	Rank	2	9	7	4	3	8	6	1	5
F24	Best	3.1967E+03	6.3159E+03	4.6170E+03	3.5538E+03	3.5144E+03	7.8948E+03	4.7439E+03	3.4938E+03	3.7366E+03
	Avg	3.2189E+03	7.7250E+03	4.9164E+03	3.6117E+03	3.5331E+03	8.6365E+03	5.6459E+03	4.5048E+03	3.8037E+03
	Std	2.2558E+01	1.1846E+03	3.7935E+02	5.1638E+01	1.5957E+01	9.6079E+02	5.3655E+02	9.0755E+02	6.1218E+01
	Rank	1	8	6	3	2	9	7	5	4
F25	Best	1.0890E+04	1.8456E+04	1.7562E+04	1.3904E+04	1.3626E+04	1.8606E+04	1.6670E+04	8.5862E+03	1.4546E+04
	Avg	1.3891E+04	2.1812E+04	1.8696E+04	1.4065E+04	1.4080E+04	1.9875E+04	1.8977E+04	1.0929E+04	1.4657E+04
	Std	2.1456E+03	3.7240E+03	7.2192E+02	1.6573E+02	2.9501E+02	1.4324E+03	2.0657E+03	1.9896E+03	8.6922E+01
	Rank	2	9	6	3	4	8	7	1	5
F26	Best	3.2000E+03	3.9018E+03	3.8074E+03	3.4116E+03	3.3994E+03	4.5302E+03	3.8809E+03	3.5287E+03	3.6936E+03
	Avg	3.6020E+03	4.1419E+03	3.9447E+03	3.4738E+03	3.4136E+03	4.6310E+03	3.9918E+03	3.6946E+03	3.7406E+03
	Std	2.9031E+02	1.8102E+02	1.0736E+02	5.6774E+01	1.2143E+01	9.4729E+01	1.0460E+02	1.6294E+02	4.3592E+01
	Rank	3	8	6	2	1	9	7	4	5
F27	Best	3.2718E+03	8.0033E+03	5.8814E+03	3.7345E+03	3.5343E+03	9.9385E+03	7.5866E+03	3.5610E+03	4.0074E+03
	Avg	3.2993E+03	8.9719E+03	6.3603E+03	3.7875E+03	3.5610E+03	1.0738E+04	9.0128E+03	5.0413E+03	4.1158E+03
	Std	2.6190E+01	7.3870E+02	4.2891E+02	5.6185E+01	1.9434E+01	5.5354E+02	1.5028E+03	1.9962E+03	7.5592E+01
	Rank	1	7	6	3	2	9	8	5	4
F28	Best	4.0991E+03	1.0137E+04	9.1411E+03	8.9116E+03	8.5558E+03	9.6930E+03	8.9844E+03	6.5009E+03	8.7843E+03
	Avg	4.7295E+03	1.1625E+04	9.8342E+03	9.1304E+03	8.8456E+03	1.0337E+04	9.8153E+03	6.9787E+03	9.3713E+03
	Std	4.2458E+02	1.1352E+03	6.0825E+02	1.6976E+02	2.4390E+02	4.7938E+02	6.6407E+02	4.5606E+02	5.2611E+02
	Rank	1	9	7	4	3	8	6	2	5
F29	Best	4.6070E+03	8.1812E+07	1.0910E+07	1.9050E+06	2.4676E+06	2.3263E+08	2.1026E+08	3.2257E+04	7.4875E+06
	Avg	7.1070E+03	1.5251E+08	4.4490E+07	3.0496E+06	3.4087E+06	3.1959E+08	3.0248E+08	3.0475E+06	8.4412E+06
	Std	2.4952E+03	4.4907E+07	2.8090E+07	1.3875E+06	6.1816E+05	9.9881E+07	7.4762E+07	4.0098E+06	6.7657E+05
	Rank	1	7	6	3	4	9	8	2	5
